# Temporal trends in use of tests in UK primary care, 2000-15: retrospective analysis of 250 million tests

**DOI:** 10.1136/bmj.k4666

**Published:** 2018-11-28

**Authors:** Jack W O’Sullivan, Sarah Stevens, F D Richard Hobbs, Chris Salisbury, Paul Little, Ben Goldacre, Clare Bankhead, Jeffrey K Aronson, Rafael Perera, Carl Heneghan

**Affiliations:** 1 Centre for Evidence-Based Medicine, Nuffield Department of Primary Care Health Sciences, University of Oxford, Oxford OX2 6GG, UK; 2 Nuffield Department of Primary Care Health Sciences, University of Oxford, Oxford, UK; 3 Center for Inherited Cardiovascular Disease, Stanford University, Stanford, CA, USA; 4 Meta-Research Innovation Center at Stanford (METRICS), Stanford University, Stanford, CA, USA; 5 Centre for Academic Primary Care, Department of Population Health Sciences, Bristol Medical School, University of Bristol, Bristol, UK; 6 Primary Care and Population Sciences, University of Southampton, Southampton, UK

## Abstract

**Objectives:**

To assess the temporal change in test use in UK primary care and to identify tests with the greatest increase in use.

**Design:**

Retrospective cohort study.

**Setting:**

UK primary care.

**Participants:**

All patients registered to UK General Practices in the Clinical Practice Research Datalink, 2000/1 to 2015/16.

**Main outcome measures:**

Temporal trends in test use, and crude and age and sex standardised rates of total test use and of 44 specific tests.

**Results:**

262 974 099 tests were analysed over 71 436 331 person years. Age and sex adjusted use increased by 8.5% annually (95% confidence interval 7.6% to 9.4%); from 14 869 tests per 10 000 person years in 2000/1 to 49 267 in 2015/16, a 3.3-fold increase. Patients in 2015/16 had on average five tests per year, compared with 1.5 in 2000/1. Test use also increased statistically significantly across all age groups, in both sexes, across all test types (laboratory, imaging, and miscellaneous), and 40 of the 44 tests that were studied specifically.

**Conclusion:**

Total test use has increased markedly over time, in both sexes, and across all age groups, test types (laboratory, imaging, and miscellaneous) and for 40 of 44 tests specifically studied. Of the patients who underwent at least one test annually, the proportion who had more than one test increased significantly over time.

## Introduction

The National Health Service is experiencing an unprecedented rise in spending.^1^ Since 2003, net expenditure has increased by more than £50bn ($65bn; €57bn), around an 80% rise.^1^ These increasing costs threaten the sustainability of the NHS.

Despite the substantial workload provided in primary care,^2^ few studies have explored resource use in this setting. No official statistics exist on one of the core services provided by general practitioners in the United Kingdom—the use of tests. One study examined the use of a few specific tests between 2005 and 2009 but in a restricted population sample.^3^

More than 70% of clinical decisions are influenced by test results,^4^ and tests contribute substantially to NHS expenditure. Lord Carter’s 2006 review found that laboratory tests in both primary and secondary care account for up to £3bn of the annual NHS budget (this estimate includes direct and indirect (staff) costs).^4^ General practice is the single largest contributor to test use, with almost 45% of requests for tests arising from primary care.^4^

We quantified the temporal changes in test use in UK general practice and identified specific tests with the greatest increase in use.

## Methods

### Study population

We obtained electronic health record data from patients registered with general practices contributing to the Clinical Practice Research Datalink (CPRD) from 1 April 2000 to 31 March 2016. The CPRD, a large database of anonymised electronic health records from UK primary care, contains patient level data covering about 7% of the UK population.^5^ The CPRD was chosen over other UK primary care databases because it has been validated extensively and is representative of the UK population for age, sex,^5^ and ethnicity.^6^ We included patients of any age if their records were acceptable for research purposes (a data quality indicator provided by CPRD) and they were registered at practices with continuous high quality data reporting (CPRD defined up to standard)^7^ at any time during the study period.

### Included tests

We studied all tests recorded in the CPRD primary care record during the study period. The CPRD contains primary care data, whereas secondary care data are captured in a separate database and were not included in this study. We also excluded physical examination findings, vital signs (including blood pressure), and body weights.

Tests were grouped into one of three categories: laboratory, imaging, or miscellaneous. The miscellaneous group included tests such as spirometry, upper endoscopy, colonoscopy, cervical smears, and electrocardiography. To avoid double counting, if the same code was recorded multiple times for the same patient on the same day, it was counted as one test. Similarly, codes that referred to the same test, or separate components of a test (eg, individual components of a full blood count), were grouped and counted as one test.

We also examined 44 specific tests (28 laboratory, 11 imaging, and five miscellaneous), chosen because they are commonly used tests and are included in guidelines or in the Quality and Outcomes Framework (see supplementary file). The selected tests were discussed with our dedicated patient and public involvement group and agreed on by the study authors.

### Statistical analysis

For each year we calculated the total number of tests recorded in general practice, stratified by age and sex. We calculated total person years of observation in each age and sex stratum for each year. Patients alive and registered for the entire year contributed one person year of observation to the total. Patients who were born, died, registered, or deregistered during the year were included, but we adjusted their contribution proportionately to the person year calculation (eg, a patient who was registered and alive for only six months contributed 0.5 person years).

We calculated crude rates of test use per 10 000 person years in each age and sex stratum for each year. For comparison across years, we standardised crude rates to the mid-2015 UK population.^8^ Rates were calculated for the total number of tests, each of the 44 specific tests, and the three groups of tests.

Joinpoint regression was used to model the temporal changes in age and sex standardised rates.^9^ We examined temporal changes from 2000/1 to 2015/16, and, to account for any potential coding inconsistencies early in our study period, we conducted a sensitivity analysis for the period after the introduction of the Quality and Outcomes Framework (2004/5 to 2015/16). The locations of significant changes in slope (joinpoints) were identified, as were the annual percentage changes between joinpoints. We also calculated the average annual percentage change^10^ across the entire study period (2000/1 to 2015/16) and for the period after the introduction of the Quality and Outcomes Framework (2004/5 to 2015/16). For the 44 tests, we calculated the median annual percentage change and absolute change in test rate for tests included and not included in the Quality and Outcomes Framework.

We also explored whether the temporal change in test use was related to a change in the number of tests ordered per patient or an increase in the number of patients who underwent tests. To do this, we calculated the percentage of test use that was ordered for patients who received one test or more than one test (up to 100 tests per patient) during the years 2000/1, 2004/5, and 2015/16. For example, we calculated the proportion of tests in 2000/1 that were ordered for patients who received only one test all year. We examined the differences between these proportions across years using the χ^2^ test and report the percentage differences, with 95% confidence intervals and P values. We used Stata (version 14.2) for data extraction and cleaning, and R (version 3.4.1) for statistical analyses.

### Patient and public involvement

The grant supporting this study has a dedicated patient group who were involved in refining the research question and protocol. A patient and public involvement focus group assisted with lay summaries. 

## Results

Overall, 262 974 099 tests were ordered for 11 082 628 patients over 71 436 331 patient years from 1 April 2000 to 31 March 2016.

### Total test use

**Fig 1:**
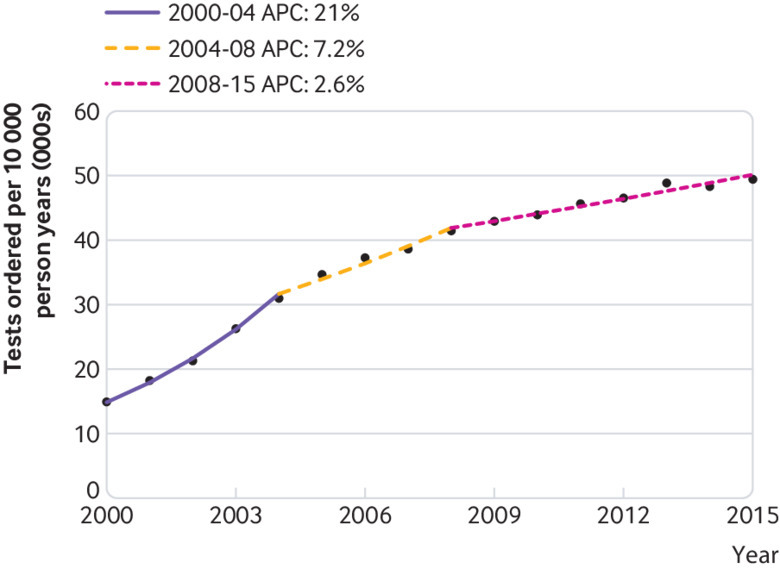
Temporal trends in total test use. APC=annual percentage change

The age and sex adjusted rate of total test use increased from 14 869 tests per 10 000 person years in 2000/1 to 49 267 in 2015/16 (table 1, fig 1), an annual increase of 8.5% (95% confidence interval 7.6% to 9.3%). The annual rate of test use also increased significantly after the introduction of the Quality and Outcomes Framework, although at a lower rate (4.3%, 3.4% to 5.1%). The slope of the trend line changed significantly at two points: 2004/5 (P<0.001) and 2008/9 (P=0.004) (fig 1).

**Table 1:** Age and sex adjusted rates of test use per 10 000 person years

Age group (years)	Adjusted test use rate/10 000 person years, by year															
	2000/1	2001/2	2002/3	20034	2004/5	2005/6	2006/7	2007/8	2008/9	2009/10	2010/11	2011/12	2012/13	2013/14	2014/15	2015/16
0-4	127.6	141.0	151.6	189.4	213.8	240.8	249.0	256.7	271.3	274.9	283.8	288.7	295.9	305.2	308.7	299.6
5-14	288.1	319.7	349.0	410.4	458.1	508.0	508.1	532.7	569.3	579.0	605.9	624.5	659.3	696.3	710.3	716.9
15-24	913.9	1049.0	1174.8	1397.6	1568.7	1723.6	1781.9	1856.1	2034.7	2091.4	2153.6	2281.4	2324.6	2424.1	2466.4	2530.5
25-44	2844.7	3394.5	3850.5	4641.4	5294.8	5976.8	6243.7	6503.4	7123.9	7318.6	7518.9	7957.2	8091.7	8464.1	8548.2	8720.2
45-64	4839.8	5842.8	6757.0	8306.7	9781.8	10 933.8	11 700.9	12 016.9	12 865.4	13 266.5	13 566.0	14 098.0	14 391.3	15 047.6	14 773.2	15 233.1
65-74	3055.9	3820.3	4523.4	5657.1	6775.7	7533.2	8171.3	8401.0	8847.5	9166.2	9288.8	9500.7	9585.0	10 039.2	9793.3	10 009.8
75-84	1973.5	2517.2	3015.8	3882.2	4727.6	5284.7	5812.9	6087.8	6493.9	6842.9	6999.7	7246.5	7408.2	7834.3	7736.0	7858.4
≥85	825.6	1077.1	1297.0	1684.2	2096.4	2392.2	2644.9	2825.3	3047.4	3262.8	3356.0	3528.1	3641.0	3902.7	3834.1	3898.5
Total	14 869.0	18 161.6	21 119.0	26 169.0	30 916.9	34 593.2	37 112.7	38 480.0	41 253.3	42 802.4	43 772.7	45 525.0	46 397.0	48 713.5	48 170.3	49 267.0

### Total test use by age group

Patients aged 45 to 64 had the highest rates of test use across all 16 study years, followed by those aged 65 to 74 and then 25 to 44 (table 1). The age and sex adjusted rates of test use increased significantly in all age groups over the study period, by at least 6.5% annually (table 2). The largest annual increase was in patients aged more than 85 (11.1%, 9.8% to 12%), followed by patients aged 75 to 84 (9.8%, 8.8% to 10.8%). The rate of test use also increased significantly in all age groups after the introduction of the Quality and Outcomes Framework (table 2). Patients aged more than 85 also had the greatest annual increase in test use after the introduction of the Quality and Outcomes Framework (5.7%, 4.6% to 6.8%), followed by those aged 25 to 44 (4.8%, 4.0% to 5.6%).

**Table 2:** Average annual percentage increase in rate of test ordering by age group

Age group (years)	Average annual percentage increase (95% CI)	
	2000/1-2015/16	2004/5-2015/16
0-4	6.5 (5.7 to 7.4)	3.6 (3.1 to 4.2)
5-14	6.5 (5.9 to 7.0)	4.6 (4.2 to 4.9)
15-24	7.2 (6.5 to 7.9)	4.5 (3.8 to 5.2)
25-44	7.6 (6.8 to 8.4)	4.8 (4.0 to 5.6)
45-64	7.8 (7.1 to 8.5)	4.3 (3.7 to 5.1)
65-74	8.3 (7.4 to 9.2)	3.4 (2.6 to 4.4)
75-84	9.8 (8.8 to 10.8)	4.7 (3.7 to 5.7)
≥85	11.1 (9.8 to 12.4)	5.7 (4.6 to 6.8)
Total	8.5 (7.6 to 9.3)	4.3 (3.4 to 5.1)

### Test types

**Fig 2:**
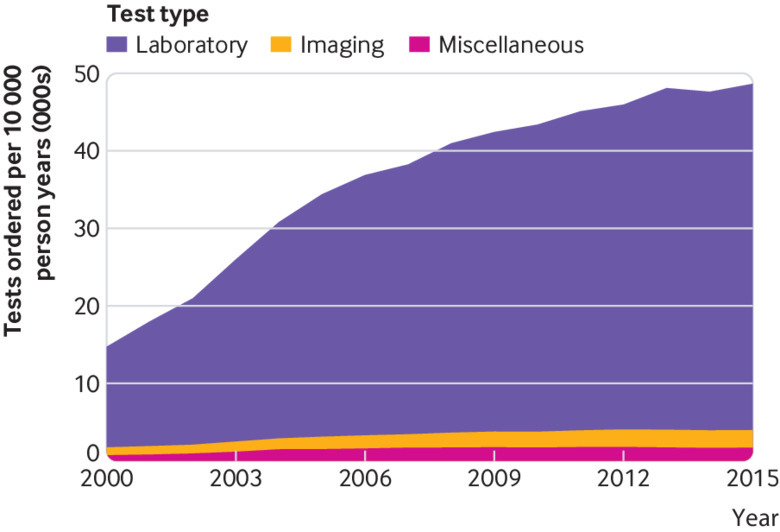
Temporal trends in total test use by test type

Laboratory tests were used substantially more often than imaging and miscellaneous tests across the entire study period (fig 2, also see supplementary file). The adjusted rates of laboratory, imaging, and miscellaneous tests all increased statistically significantly (table 3). Laboratory tests showed the greatest increase, from 13 091 tests per 10 000 person years in 2000/1 to 44 847 in 2015/16 (see supplementary file), an annual increase of 8.7% (7.8% to 9.6%). Over the same period, the use of imaging and miscellaneous tests increased annually by 5.5% (4.7% to 6.4%) and 6.3% (5.3% to 7.3%), respectively. The use of all test types increased statistically significantly after the introduction of the Quality and Outcomes Framework, albeit less so (table 3).

**Table 3:** Average annual percentage increase in rate of test use by category

Test type	Average annual percentage increase (95% CI)	
	2000/1-2015/16	2004/5-2015/16
Laboratory	8.7 (7.8 to 9.6)	4.3 (3.4 to 5.2)
Imaging	5.5 (4.7 to 6.4)	4.0 (3.0 to 5.0)
Miscellaneous	6.3 (5.3 to 7.3)	1.4 (0.5 to 2.4)

### Number of tests ordered per patient

**Fig 3:**
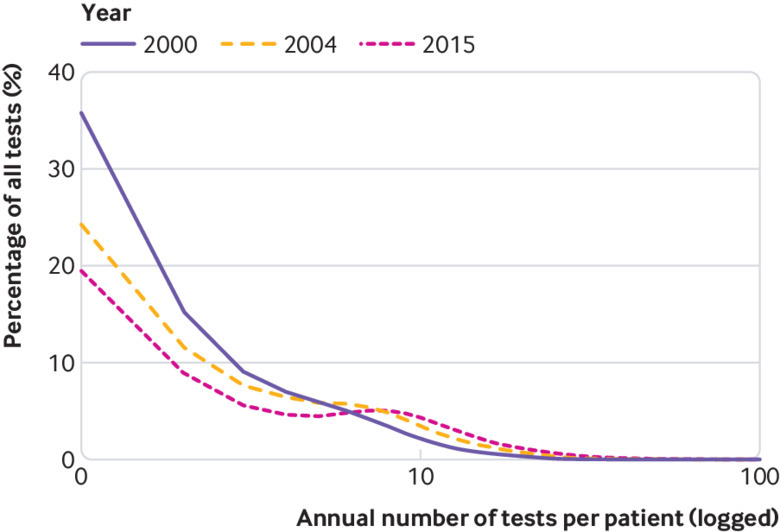
Percentage of all tests ordered by annual number of tests received per patient

A greater proportion of tests was ordered on fewer patients in 2015/16 than in 2000/1. In 2015/16, 32.7% of test use was ordered for patients who underwent more than 10 tests annually, compared with 9.5% in 2000/1 (absolute percentage change 23.2%, 95% confidence interval 23.1% to 23.3%; P<0.001). Similarly, the proportion of tests ordered for patients who received only one test was 16.4% less (95% confidence interval 16.3% to 16.5%, P<0.001) in 2015/16 than in 2000/1 (fig 3, and see supplementary table S6).

### Changes in use of specific tests

Renal function tests were the most commonly ordered test for most of the study period (2002/3 to 2015/16; see supplementary file). Full blood count was used most often in 2000/1 and 2001/2 and was the second most frequently ordered test during the remainder of the study (2002/3 to 2015/16). Liver function tests were the third most commonly ordered test from 2001/2 to 2015/16, with urine dipstick testing third in 2000/1. Pelvic computed tomography (CT) and knee magnetic resonance imaging (MRI) had the lowest adjusted rates of use (knee MRI from 2000/1 to 2006/7 and pelvic CT from 2007/8 to 2015/16).

Over the study period, knee MRI showed the highest average annual increase (69.0%, 95% confidence interval 3.4% to 106.5%), followed by vitamin D tests (53.7%, 50.2% to 57.3%) and brain MRI (47.3%, 39.5% to 55.5%) (see supplementary file). Vitamin D, knee MRI, and pelvic CT showed the greatest increases after the introduction of the Quality and Outcomes Framework (see supplementary file). Knee MRI, brain MRI, and pelvic CT, however, showed only modest increases in absolute use over the entire study period (from <1 test per 10 000 person years in 2000 to around 10 tests per 10 000 person years in 2016; see supplementary table S7 and fig S10).

Vaginal swabbing was the only test that showed a statistically significant annual decrease in use over the study period (5.2%, 95% confidence interval 4.1% to 6.3%). Over the same period, three tests (cervical smear, lumbar spine radiography, and urine testing for non-illicit drugs) had non-significant changes. The adjusted rates for four tests decreased statistically significantly after the introduction of the Quality and Outcomes Framework (creatine kinase, vaginal swabbing, smear test, and spirometry) (see supplementary file).

Five temporal patterns emerged in specific test use (also see supplementary file).

• A consistent linear increase for chest radiography and tests for vitamin B12, C reactive protein, iron, folate, clotting, and ferritin

• An initial rapid increase, followed by a less rapid increase for lumbar spine MRI, pelvic CT, knee MRI, dual energy x ray absorptiometry, brain MRI, female sex hormones, knee radiography, testosterone, renal function tests, urine albumin to creatinine ratio, bone profile, prostate specific antigen, full blood count, and liver function tests

• An initial increase followed by a plateau for echocardiography, thyroid function tests, erythrocyte sedimentation rate, upper endoscopy, urine microscopy and culture, progesterone, spirometry, vitamin D, and oestradiol

• An increase followed by a decrease (inverted U distribution) for glucose, lipids, troponin, urine dipstick, urine microalbumin, colonoscopy, smear test, creatine kinase, pelvic ultrasonography

• A decrease, an increase, and then a decrease for lumbar spine radiography and testing urine for non-illicit drugs. Two tests (glycated haemoglobin (HbA1c) and vaginal swabbing) did not adequately fit any category nor were similar to each other. Furthermore, tests included in the Quality and Outcomes Framework had a median average annual percentage increase of 9.8% (interquartile range 6.6-12.8%) compared with 7.4% (4.6-16.4%) for tests not included in the Quality and Outcomes Framework (see supplementary table S9). The median absolute increase in tests included in the Quality and Outcomes Framework (830.9, interquartile range 250.3-1516.3) was also larger than for tests not included in the Quality and Outcomes Framework (57.2, 17.4-192.2).

## Discussion

We analysed the temporal change in tests ordered from UK primary care. The total use of tests increased markedly over time, even after adjustment for population growth; patients in 2015/16 had on average five tests per year, compared with 1.5 in 2000/1. Test use increased in men and women (a 3.4-fold and 3.3-fold increase, respectively, see supplementary figure S9) and in all age groups; elderly patients had the greatest increase (a 4.6-fold increase for those aged more than 85). All types of tests (laboratory, imaging, and miscellaneous) increased statistically significantly, as did 40 of the 44 tests we studied specifically. Furthermore, there was a significant increase in the number of tests ordered per patient; of the patients who underwent at least one test annually, the proportion who had more than one test increased significantly over time. In light of unprecedented financial strains on the NHS, accurate data on core primary care services are essential. These results allow policymakers to assess trends in the use of tests, stratified by the type of test and the age and sex of patients, providing guidance for healthcare resource planning.

Many factors probably contribute to the increasing use of tests in UK primary care. Increased use could be a direct response to the increasing number and duration of consultations with general practitioners,^2^ with tests reportedly being used for “strategic, non-medical reasons,” such as reassuring patients and ending consultations.^11^ The increase in test use we report might also be due to changes to NHS services. Over the study period, general practitioners’ access to diagnostic tests expanded,^12^ many services were diverted from secondary to primary care,^13^ and the Quality and Outcomes Framework was introduced. The framework incentivises the monitoring of chronic diseases, which could partially explain the increase in laboratory tests. Additionally, increased use of testing by general practitioners might reflect a change in the patient-doctor relationship, including a greater expectation from patients that they should be tested.^14^ With the advent of shared decision making—aided by campaigns such as Choosing Wisely^15^—patients are increasingly informed and encouraged to discuss healthcare choices. Evidence suggests that both clinicians^16^ and patients^17^ overestimate the benefits and underestimate the harms of tests; this is likely to contribute to more testing. Lastly, increases in test usage might more broadly reflect the possible medicolegal consequences of undertesting^18^ and the lack of disincentives to over-investigate.

Changes in the use of specific tests give further insights into the factors that increase use. The four haematinic tests (iron, ferritin, vitamin B12, and folate) followed the same linear pattern, as did tests for C reactive protein. These tests are typically used for patients with non-specific symptoms^11^ and might reflect clinician’s increased need to rule out disease in these patients. Imaging largely followed a pattern of a rapid increase that then diminished. This pattern may relate to time points when general practitioners gain direct access to imaging.

### Implications

Whatever factors contribute to increased test use, our results are likely to have major implications for general practitioners’ workload. Greater test use is likely to lead to more consultations and an increase in non-consultation workload. No data are available on the time it takes UK general practitioners to review test results. However, a US study suggested that primary care physicians on average spend two or three minutes reviewing a test (70 minutes a day).^19^ Using this estimate and the approximate average number of patients (n=7000) and general practitioners (n=3) in each general practice (which have changed minimally since 2005),^20^ we estimate that the average general practitioner spent 1.5 to 2 hours reviewing test results each workday in 2015/16. This is a more than threefold increase from the estimated 2000/1 figure of 25 to 35 minutes daily. Our results support other evidence that suggests general practice workload in the UK is reaching saturation point.^2^

Similarly, our results indicate the burden of tests ordered by general practitioners on NHS expenditure. Using conservative estimates from the National Institute for Health and Care Excellence^21^ (£6 for a laboratory test, £29 for imaging, and £53 for miscellaneous tests), we estimate that general practice related test use cost £2.8bn in 2015/16 (£1.8bn for laboratory tests, £400 000 for imaging, and £600 000 for miscellaneous tests). Notably, these estimates are likely an underestimation of the true costs. Our estimates only account for the direct cost of tests and do not include the cost of general practitioners reviewing the result or the administration team processing the result.

### Strengths and weaknesses in relation to other studies

There are no official statistics on the use of tests in the UK. A 2013 study assessed temporal changes in 29 laboratory tests ordered from UK primary care during 2005-09 in a random sample of 100 000 patients.^3^ The authors also calculated an average temporal change for all 29 tests. Our study, in comparison, was not exclusive in its selection of tests or patients—we assessed the change in use of all tests, in all registered patients over a longer period. We also assessed more tests individually and all types of tests over time (laboratory, imaging, and miscellaneous). Nevertheless, our results over the same period are similar; we found that the annual number of all laboratory tests increased from around three tests per patient in 2005 (31 306 per 10 000 person years) to four tests per patient in 2009 (38 758 per 10 000 person years). Busby and colleagues reported that the ordering of 29 specific laboratory tests on average increased from 2.4 tests per patient (23 872 per 10 000 person years) to 3.0 tests per patient (29 644 per 10 000 person years) over the same period.^3^ Furthermore, our results are in line with data from other countries. An Australian study reported a 54% increase in laboratory test ordering from 2000/1 to 2007/8,^22^ whereas in the US from 1997 to 2006, CT and MRI increased by 14% and 26% annually.^23^

We have not examined the appropriateness of the changes in total and specific test use. It is plausible that a substantial proportion of the temporal changes we found represents over-testing. Although the definition remains contentious,^24^ over-testing occurs when a test is ordered unnecessarily; where the harms outweigh the benefits.^25^ Previous studies have used guidelines as a metric to define the appropriate use of tests,^25^ but evidence suggests a variable quality of guideline recommendations related to diagnostic tests.^26^ This limitation makes the methods for detecting unnecessary testing a challenge. Nevertheless, over-testing is thought to be common and increasing in healthcare systems, particularly in developed countries.^25^ ^27^ ^28^ Although our results do not explicitly quantify over-testing, we have identified tests with the greatest temporal change in use, and we graphically display the tests that have shown recent, exponential increases in use (see supplementary figure S10).

Although our data did not allow us to explicitly explore the appropriateness of changes in test use, some tests are more likely to have been inappropriately overused. Similarly, there are some tests where the changes we report are likely to be appropriate. Over the study period, testing for vitamin D increased exponentially; an average annual increase of more than 54% (the second most of any test). In absolute terms, 182 more vitamin D tests were ordered per 10 000 person years in 2015 compared with 2000. Similar increases have been noted in other high income countries, including Australia,^29^ Canada,^30^ ^31^ and the US.^32^ Although the appropriateness of vitamin D test use has not been explicitly examined in the UK, it has been in other high income countries that have experienced similar increases.^30^ ^31^ ^32^ In Canada, up to 40% of vitamin D tests are estimated to be incongruent with guidelines—that is, the test is a repeat test within three months of the original test or the test is ordered for a patient who has already had two tests that year.^30^ Similarly in the US, 44% of vitamin D tests were estimated to be “unnecessary screening.”^32^ In the UK, there is evidence that most vitamin D tests return a normal result.^33^ This might suggest that many of the vitamin D tests ordered are unnecessary screening.

The non-significant change in use of lumbar spine radiography is of concern. In early 2000 numerous randomised controlled trials investigated the effectiveness of imaging for non-red flag low back pain.^34^ ^35^ ^36^ ^37^ ^38^ These studies concluded that imaging for low back pain without red flags does not benefit patients, nor is it cost effective. Despite this evidence, our results suggest no major change in lumbar spine radiography ordered in 2015 compared with 2000. Furthermore, the use of lumbar spine MRIs has increased significantly over the study period. Without any clear justification, the use of lumbar spine MRIs has increased by 15% a year and, in absolute terms, 53 more tests per 10 000 person years. Imaging tests are labour intensive and not without potential harm.^39^

Conversely, the increase in use of HbA1c tests and the plateau of urine microscopy and culture test use is likely to reflect appropriate changes. The HbA1c test was originally approved exclusively to monitor patients with diabetes. In 2011, however, the World Health Organization approved the use of the test to diagnose as well as monitor diabetes.^40^ This guideline change is likely to have contributed to the significant increase in HbA1c test use from this date.

In 2006, the Scottish Intercollegiate Guidelines Network guideline recommended a clinical diagnosis of urinary tract infection,^41^ also adopted by NICE. This guideline change coincided with a plateau in the number of urine microscopy and culture tests ordered. From 2007, the average annual increase in these tests was 0.1% (95% confidence interval −0.7% to 0.9%), compared with 9.3% (8% to 10.7%) from 2000 to 2007. For most tests, however, further investigation into the causes and appropriateness of these changes is still required.

### Limitations of this study

A potential limitation of our analysis is the inconsistency of coding across the study period. To deal with this we only included general practices using CPRD quality assured data. Furthermore, we considered the ordering of a test as either a record indicating that the test had been ordered or evidence of the test result (eg, by letter or by a numerical result). Letter correspondence is often incorporated into CPRD, with around 90% compliance.^42^ ^43^ We therefore captured tests when the order was not directly documented. To mitigate double counting, if the same test appeared in a patient record twice on one day, we matched these data entries and counted only one test.

Furthermore, previous literature suggests that test coding within CPRD has been accurate and stable over time. A systematic review reports that all abnormal test results are recorded accurately in CPRD.^44^ A separate study^3^ reports that from 2000 the proportion of abnormal test results, out of the total number of tests ordered, has remained relatively constant in CPRD (28.7% from 2000 to 2004 and 27.0% from 2005 onwards). These results suggest that the coding of all test results (normal and abnormal) has been consistent from 2000.

The introduction of the Quality and Outcomes Framework improved the frequency of data entry in general practitioner records^45^; 90% of doctors were electronically ordering tests from this date.^3^ ^46^ Thus, to further investigate any effects of potentially incomplete coding in the early 2000s, we conducted a sensitivity analysis, restricting the study period to after the introduction of the Quality and Outcomes Framework. Comparison of our primary and sensitivity analyses shows a consistent direction of results, with the magnitude of increase greater in the main analysis.

The results from our analysis of 44 specific tests suggest that coding before 2004/5 was valid. The use of three tests (laboratory, imaging, and miscellaneous) decreased statistically significantly from 2000 to 2003/4. If the data were not coded appropriately, and thus tests were missed before the introduction of the Quality and Outcomes Framework, we would not have anticipated any reductions in the use of any test. It is of further encouragement that we noted reductions in the use across all three test types (laboratory, imaging, and miscellaneous).

Nevertheless, even if the earlier period is discounted (2000/1 to 2003/4) our sensitivity analysis suggests the same conclusion: a significant increase in the use of tests over time (see supplementary file).

Another potential limitation of our study is the contamination of secondary care data within the CPRD. Given the CPRD’s extensive use and validation as a primary care database and the existence of a dedicated secondary care database (Hospital Episode Statistics), we feel this is unlikely. The temporal trends in brain and knee MRI also further support the lack of contamination from secondary care data (see supplementary file).

Although data in the CPRD are available before 2000, we examined test use from this date for several reasons—namely, the advent of NICE (1999) and the emergence of evidence based practice in UK primary care. Because of our concerns about the quality of data before 2000 we were advised by CPRD experts to start the analysis from 2000. Finally, although the CPRD database is representative of the UK population in terms of age, sex,^5^ and ethnic background,^6^ it only extracts data from general practices that use the Vision GP system. Practices that use this system are located around the UK, but are concentrated in the south of England.^47^

### Further research

Future research should focus on determining the appropriateness of the temporal changes we have reported. Ideally, this research should use individual patient data to measure appropriateness directly against evidence based criteria. We suggest an initial focus on the tests with the greatest temporal changes.

Further research can also investigate factors that increase the use of tests in particular patient groups, such as people older than 85, in whom test ordering showed the greatest increase. Additional research investigating the consequences of accelerating test use (eg, on specialist referral and treatment) would also be valuable.

Some of our team are involved in delivering OpenPathology.net 28; an open data tool (similar to OpenPrescribing.net) that provides easy access to various analytical approaches identifying test ordering behaviour in primary care. This tool will continue our work exploring temporal trends on a live interface.

### Conclusion

The use of tests from UK general practices is increasing substantially; in both sexes and across all patient ages, test types, and 40 of 44 tests specifically studied. These changes have outstripped population growth. In light of unprecedented financial strains on the NHS, these results allow policymakers to assess trends in the use of tests, stratified by the type of test and the age and sex of patients. Our results will provide guidance for future healthcare resource planning.

**What is already known on this topic**

Few data quantify the use of tests in primary care internationally
High income countries (Australia, United States) have published reports showing temporal increases in test use
In the United Kingdom, one study showed an increase in use of laboratory tests during 2005-09, but this was in a limited sample, only assessed laboratory tests, and was in a restricted population

**What this study adds**

Total test use has increased markedly over time, in both sexes, and across all age groups, test types (laboratory, imaging, and miscellaneous) and for 40 of 44 tests specifically studied
Of the patients who underwent at least one test annually, the proportion who had more than one test increased significantly over time
In light of unprecedented financial strains on the National Health Service, accurate data on core primary care services are essential

## Web extra

Extra material supplied by authors

## Notes

Contributors: JOS, CH, RP, BG, and FDRH conceived the study. JOS, RP, CB, and SS designed the study, which was further refined by general practitioner experts CH, FDRH, PL, and CS. JOS drafted the protocol, which all authors contributed to and revised critically. JOS and SS were responsible for data management. JOS, SS, and RP did the statistical analyses. JOS drafted the manuscript, to which all authors contributed, revised critically, and approved. JOS is the guarantor. The corresponding author attests that all listed authors meet authorship criteria and that no others meeting the criteria have been omitted.

Ethical approval: This study was approved by Trent MultiResearch Ethics Committee (reference No 05/MRE04/87). The protocol was approved by the independent scientific advisory committee of the MHRA (protocol No 17_06R; available from the authors on request).

Data sharing: The General Practices in the Clinical Practice Research Datalink (CPRD) is run by the UK Department of Health. All the data are available via an application to the CPRD. Data acquisition is associated with a fee.

Transparency: The manuscript’s guarantor (JOS) affirms that the manuscript is an honest, accurate, and transparent account of the study being reported; that no important aspects of the study have been omitted; and that any discrepancies from the study as planned (and, if relevant, registered) have been explained.
