# Supplementary material for: Temporal trends in use of tests in UK primary care, 2000-15: retrospective analysis of 250 million tests
Source: BMJ. 2018 Nov 28;363:k4666. doi: 10.1136/bmj.k4666 (PMC6260131; doi:10.1136/bmj.k4666)
Supplement: Supplementary file 1 — Supplementary information: additional information, tables, and figures [file osuj044789.ww1.pdf]

Supplementary file

Contents

Text S1 list of included tests........ 2

Text S2 Extended included tests........ 21

Text S3: Extended Discussion........ 23

Table S4 Age and Sex Adjusted rates of test use per 10,000 person-years, stratified by sex........ 25

Table S5 Age and sex adjusted rates of test use per 10,000, by test type........ 26

Table S6 Changes in the proportion of tests ordered for patients that received many tests........ 27

Table S7 Age and sex adjusted rates of specific test use per 10,000 person-years........ 28

Table S8 Average annual percentage increase in specific test use........ 30

Table S9 A comparison of tests included and not included in the Quality Outcomes Framework........ 33

Table S10 Tests included and not included the Quality Outcomes Framework........ 34

Figures S9 Temporal trends in test use stratified by Sex........ 39

Figures S10 AAPC plotted against absolute change in test use per 10,000 person years........ 40

Figures S11 Temporal trends in specific test use........ 41

Text S1 list of included tests

List of tests within total tests

Imaging

1. Abdominal aortography
2. Abdominal fistulography
3. ABPI - Ankle brachial pressure index
4. Angiocardiography
5. Antenatal scan
6. Aortography
7. Arm arteriography
8. Arteriography - lower limb
9. Arteriography - upper limb
10. Arteriography of carotid artery
11. Arthrography of knee
12. Barium enema
13. Barium follow through
14. Barium meal
15. Barium swallow
16. Bone densitometry
17. Brain isotope studies
18. Breast contrast radiog
19. Bronchography
20. Cardiac isotope studies
21. Carotid artery angiography
22. Carotid artery doppler
23. CAT scan - abdomen

24. CAT scan - face
25. CAT scan - neck
26. CAT scan - thorax
27. CAT scan - whole body
28. Cholecystogram
29. Cineradiography - GIT
30. Cineradiography - GUT
31. Cineradiography - joints
32. Cineradiography - resp. tract
33. Computed tomography angiography of coronary arteries
34. Computed tomography arteriogram of thorax
35. Computed tomography abdomen
36. Computed tomography brain
37. Computed tomography chest
38. Computed tomography gall bladder
39. Computed tomography head/sinuses
40. Computed tomography kidney
41. Computed tomography lower limb
42. Computed tomography neck
43. Computed tomography pelvis
44. Computed tomography shoulder
45. Computed tomography sternoclavicular joint
46. Computed tomography upper limb
47. Computed tomography urinary tract
48. Computed tomography pelvis
49. Computed tomography pulmonary angiography
50. Computerised bone densimetry
51. Contrast neurodiography
52. Contrast radiog.abd.cavity
53. Contrast radiog.larynx/trachea

54. Contrast radiog.peritoneal cav
55. Contrast radiogr. oropharynx
56. Contrast radiogr.abdom.cavity
57. Contrast radiogr.nasopharynx
58. Contrast radiogr.resp.organs
59. Contrast radiography - ducts
60. Contrast radiography GIT
61. Contrast radiography head/neck
62. Coronary arteriography
63. CSF isotope study
64. Dental radiography
65. Doppler ultrasound of vessels of extremities
66. Dual-energy X-ray absorptiometry (DEXA)
67. Dynam.non-im.isotope: brain
68. Dynam.non-im.isotope: heart
69. Dynam.non-im.isotope: liver
70. Dynam.non-im.isotope: thorax
71. Dynam.non-im.isotope: thyroid
72. Dynam.non-im.isotope: urinary
73. Dynam.non-im.isotope:blood flo
74. Echocardiogram
75. Elbow arthrogram
76. Encephalography
77. Femoral arteriography
78. Head fistulography
79. Heart isotope studies
80. Hip arthrogram
81. Hormone radioassay-parathyroid
82. Hormone radioassay-sex/placent
83. Hormone radioassay - adrenal

84. Hormone radioassay - pituitary
85. Hormone radioassay - thyroid
86. Hypothalamus hormone radioass.
87. Hysterography
88. Hysterosalpingography
89. I-V pyelography
90. Intervertebral disc arthrogram
91. Intravenous cystography
92. Intravenous pyelography
93. Intravenous urogram
94. Isotope B12 + iron absorption
95. Isotope bone scan
96. Isotope brain angiography
97. Isotope diagnostic radiology
98. Isotope distribut.static scan
99. Isotope dynamic heart scan
100. Isotope dynamic liver scan
101. Isotope lung perfusion scan
102. Isotope lymphography
103. Isotope phlebography
104. Isotope scan spleen
105. Isotope stat scan parathyroid
106. Isotope static cystography
107. Kids.,urets, bladder abdo xray
108. Knee arthrography
109. Left anterior tibial doppler pressure
110. Left brachial doppler pressure
111. Left dorsalis pedis doppler pressure
112. Left peroneal doppler pressure
113. Left posterior tibial doppler pressure

114. Left toe brachial pressure index
115. Leg arteriography
116. Liver isotope studies
117. Lower limb arteriogram
118. Lower limb venogram
119. Lung isotope studies
120. Lymph isotope studies
121. Male genital contrast radiog.
122. Male genital venogram
123. Mammary contrast radiog
124. Mammary ductogram
125. Mammography
126. Micturating cystogram
127. MRI abdomen
128. MRI of Brain
129. MRI of cervical spine
130. MRI of femur
131. MRI of knee
132. MRI of lower limb
133. MRI of lumbar spine
134. MRI of shoulder
135. MRI of thoracic spine
136. MRI of thorax
137. MRI of upper limb
138. Myelography
139. Myocardial perfusion scan
140. Neck fistulography
141. Nuchal scan
142. Octreotide scan
143. Optical coherence tomography

144. Per-oral cholangiography
145. Percutaneous cystography
146. Plethysmography
147. Pneumoencephalography
148. Quantitative ultrasound scan of heel
149. Renal arteriography
150. Renal isotope studies
151. Renal soft tis. X-ray
152. Right anterior tibial doppler pressure
153. Right brachial doppler pressure
154. Right dorsalis pedis doppler pressure
155. Right posterior tibial doppler pressure
156. Right toe brachial pressure index
157. Shoulder arthrogram
158. Sialography
159. Sinuses contrast radiography
160. Skeletal survey
161. Tc-99m hexamethylpropyleneamine oxime leucocyte bowel scan
162. Tc-99m hexamethylpropyleneamine oxime leucocyte hip scan
163. Tc-99m hexamethylpropyleneamine oxime leucocyte knee scan
164. Tc-99m hexamethylpropyleneamine oxime leucocyte scan
165. Tc99m-DMSA renal study
166. Tc99m-DTPA renogram
167. Tc99m-MAG3 renogram
168. Temporomandibular arthrogram
169. Thalamus hormone radioassay
170. Transrectal ultrasound scan of prostate
171. Ultra-sound scan - obstetric
172. Ultrasonic doppler for fetal heart sounds
173. Ultrasound of abdomen

174. Ultrasound of bladder
175. Ultrasound of scrotum
176. Ultrasound scan of Achilles tendon
177. Ultrasound scan of aorta
178. Ultrasound scan of back
179. Ultrasound scan of buttock
180. Ultrasound scan of calcaneum
181. Ultrasound scan of chest
182. Ultrasound scan of chest wall
183. Ultrasound scan of elbow
184. Ultrasound scan of fingers
185. Ultrasound scan of foot
186. Ultrasound scan of forearm
187. Ultrasound scan of gall bladder
188. Ultrasound scan of hand
189. Ultrasound can of kidneys
190. Ultrasound scan of liver
191. Ultrasound scan of lower leg
192. Ultrasound scan of pelvis
193. Ultrasound scan of popliteal artery
194. Ultrasound scan of sacral spine
195. Ultrasound scan of salivary glands
196. Ultrasound scan of thigh
197. Ultrasound scan of thyroid
198. Ultrasound scan of upper abdomen
199. Ultrasound scan of upper arm
200. Ultrasound scan of wrist
201. Urethrography
202. Venogram - renal
203. Venography - azygos

204. Ventriculography - contrast
205. Vitamin B12 isotope studies
206. X-ray abdomen
207. X-ray ankle joint
208. X-ray carpus
209. X-ray cervical spine
210. X-ray chest
211. X-ray clavicle
212. X-ray coccyx
213. X-ray elbow
214. X-ray facial sinuses
215. X-ray femur
216. X-ray foot
217. X-ray frontal sinuses
218. X-ray hand
219. X-ray head of femur
220. X-ray head of humerus
221. X-ray hip joint
222. X-ray hip/leg
223. X-ray humerus
224. X-ray jaw
225. X-ray knee
226. X-ray lumbar spine
227. X-ray mandible
228. X-ray maxilla
229. X-ray maxillary sinuses
230. X-ray metacarpals
231. X-ray metatarsal bones
232. X-ray nasal/malar
233. X-ray neck of femur

234. X-ray of radius
235. X-ray of radius/ulna
236. X-ray of toes
237. X-ray of ulna
238. X-ray of wrist
239. X-ray orbit
240. X-ray pelvis
241. X-ray phalanges of toes
242. X-ray pubis
243. X-ray radius
244. X-ray ribs
245. X-ray sacro-iliac joint
246. X-ray sacrum
247. X-ray sacrum/coccyx
248. X-ray scaphoid
249. X-ray scapula
250. X-ray shaft of femur
251. X-ray shaft of humerus
252. X-ray shoulder joint
253. X-ray shoulder/arm
254. X-ray sternoclavicular joint
255. X-ray skull
256. X-ray sternum
257. X-ray tarsus
258. X-ray teeth
259. X-ray temporomandib.jnt
260. X-ray thoracic cage
261. X-ray thoracic spine

Laboratory

1. Acid phosphatase
2. Alpha fetoprotein
3. B12 levels
4. Carbemazepine monitoring
5. Serum chloride
6. Digoxin blood level
7. Follicle stimulating hormone
8. Human chorionic gonadotropin
9. Lactate dehydrogenase
10. Luteinising hormone
11. Lithium levels
12. Phenobarbitone
13. Phenytoin
14. Prolactin level
15. Prostatic acid phosphatase
16. Reticulocytes
17. Theophylline
18. Total protein
19. Valproate
20. Amniocentesis
21. Clotting tests
22. Faecal occult blood
23. Glandular fever test
24. Pregnancy test
25. Seminal analysis
26. Serology
27. Sputum culture
28. Stool culture
29. Eye swab
30. Cervical swab

31. Nose swab
32. Penile swab
33. Skin swab
34. Throat swab
35. Wound swab
36. Rubella test
37. Tuberculin test
38. Amylase
39. Uric acid blood level
40. Anti mitochondrial antibodies
41. Anti nuclear antibodies
42. Blood group antibodies
43. Syphilis test
44. Viral studies
45. Guthrie test
46. Immunoglobulin
47. Plasma electrophoresis
48. Rheumatoid factor
49. Chemical function tests
50. Examination of faeces
51. Synovial fluid examination
52. Bone marrow examination
53. Sputum examination
54. Amniotic fluid examination
55. SH-antigen (hepatitis b) test
56. HIV test
57. Infection titres
58. Anti smooth muscle autoantibodies
59. Thyroid autoantibodies
60. DNA binding autoantibodies

61. Cardiac enzymes
62. Serum osmolality
63. Serum globin
64. Blood trace elements / vitamins
65. Histology
66. Other autoantibodies
67. Hepatitis a test
68. Other bacteriology tests
69. Microscopy for malarial parasites
70. Fructosamine
71. Urethral swab
72. Antostreptolysin o titre
73. Radioallergosorbent (RAST) test
74. Lipoprotein electrophoresis
75. Lupus erythematosus (LE) cells
76. Gonadotrophin
77. Serum cortisol
78. Sex hormone binding globulin
79. Other immunology tests
80. Tricyclics
81. Plasma viscosity
82. Toxicology
83. Serum amino acids
84. Blood gases
85. Vomit examination
86. Calculus examination
87. Haemoglobin variants
88. Legionella antibody test
89. Immuno electrophoresis
90. Feto-placental hormones

91. Serum bicarbonate
92. Skin prick test
93. Serum fibrinogen level
94. Transfer coefficient for CO
95. Serum parathyroid hormone
96. HLA tissue typing
97. Blood lead level
98. Chlamydia
99. Cerebro-spinal fluid examination
100. Blood alcohol level
101. Methotrexate
102. Ascitic fluid examination
103. Parietal cell autoantibodes
104. Complement tests
105. Gastrointestinal hormones
106. Serum growth hormone
107. Sputum cytology
108. Biopsy of the skin
109. Other biopsy of skin NOS
110. Microscopy culture & sensitivities
111. Serum insulin
112. Tumour markers
113. Enzymes/specific protein
114. Serum paracetamol
115. Serum cyclosporin
116. Schilling test - b12 absorption
117. Sickle cell disease screen
118. Thrombin time
119. Serum adrenaline
120. Serum noradrenaline

121. Anion gap
122. Serum aldosterone
123. Serum lactate
124. Disaccharidase tolerance test
125. Pleural fluid examination
126. Adrenal autoantibodies
127. Serum renin
128. Carboxyhaemoglobin
129. Serum adrenocorticotrophic hormone
130. Serum amiodarone
131. Immunology screening tests
132. Hepatitis B Antibody
133. Biochemical screening tests
134. Genetic observations
135. Haematology screening tests
136. Ante-natal blood tests
137. Ear swab
138. Schilling test
139. Synacthen test
140. Disaccharidase tolerance tests
141. D-xylose absorption test
142. Short tetracosactrin test
143. Nitrogen balance test
144. Pentagastrin test
145. Rubella
146. HTLV
147. Helicobacter pylori serology
148. Helicobacter pylori antibody
149. C. Difficile toxin A
150. Hepatitis B surface antigen

151. Chlamydia
152. Virus: serology
153. Mycoplasma
154. Leptospira
155. Toxoplasma
156. Legionella
157. Syphilis
158. Rubella IgM
159. Human hepes virus
160. Fungus serology
161. Epstein-Barr virus
162. Aspergillus
163. Yersinia
164. HIV
165. Treponema pallidum haemaglutination
166. Malaria
167. Hepatitis C
168. Brucella
169. Hepatitis E
170. Parasite
171. Hepatitis G
172. Bordetella
173. Respiratory syncytial virus
174. Hepatitis D
175. Dengue virus
176. Human T-lymphotropic virus
177. Influenza
178. West Nile
179. Tropheryma whipplei
180. Camplobacter serology

181. Infleunza A virus
182. Rapid human immunodeficiency virus antibody
183. Borrelia
184. Nasopharyngeal virology swab
185. Serum IgG anticardiolipins level
186. Autoantibody titre positive
187. Serum anti-cardiolipin level
188. Coeliac disease screen
189. Mitochondrial antibody level
190. DS DNA antibody by ELISA
191. Serum IgM anticardiolipins level
192. Thyroglobulin autoantibodies
193. Microsomal autoantibodies
194. Lupus circulating anticoagulant index
195. Anti-neutrophil cytoplasmic antibody level
196. Reticulin R1 autoantibody titre
197. Serum endomysium antibodies
198. Anti-gliadin antibody level
199. Albumin autoantibody level
200. Lupus anticoagulant screen
201. Islet cell antibody level
202. Thyroid peroxidase antibody level
203. Anti-nuclear IgG antibody level
204. Intrinsic factor antibody screen
205. Extractable nuclear antigen antibody level
206. Human leucocyte antigen antibody level
207. Anti-neutrophil cytoplasmic antibody screen
208. Anti liver kidney microsomal antibody level
209. Skin antibody level
210. Anti-cyclic citrullinated peptide antibody level

211. Cytoplasmic anti-neutrophil cytoplasmic antibody level
212. Liver autoantibody level
213. TSH receptor antibody level
214. Anti-thrombin III level
215. Centromere antibody level
216. dsDNA binding autoantibody level
217. Anti-nuclear antibody level
218. Anti -Hb antibody level
219. Anti-B autoantibody level
220. Intrinsic factor antibody level
221. IgA antibody level
222. Striated muscle antibody level
223. Myeloperoxidase antibody level
224. Serum glomerular basement membrane antibody level
225. Proteinase 3 antibody level
226. GBM antibody level
227. Intrinsic factor antibody
228. Anti-tissue transglutaminase level
229. ssDNA binding autoantibody level
230. Paraneoplastic antibody level
231. Acetyl choline receptor antibody level
232. Serum Ro 02-0683 number
233. ALKM - Anti liver kidney microsomal antibody level
234. Ganglioside antibody level
235. Serum anti-ganglioside M1 level
236. Cold antibody level
237. Glycolipid antibody level
238. Platelet antibodies test
239. Parathyroid antibody level
240. U-snRNP Antibody level

241. Pemphigoid antibody level
242. Pemphigus antibody level
243. Yo antibody level
244. Ri antibody level
245. Histone antibody level
246. Sperm antibody level
247. Serum voltage-gated calcium channel antibody level
248. CMV IgM antibody level
249. CMV IgG antibody level
250. Carbohydrate antigen 125 level
251. Carcinoembryonic antigen level
252. CA199 level
253. CA153 level
254. Plasma chromogranin A level
255. Squamous cell carcinoma antigen level
256. Serum amylase level
257. Plasma cholinesterase level
258. Serum transferrin
259. Serum angiotensin converting enzyme level
260. Serum caeruloplasmin
261. Serum haptoglobin
262. Serum A1 - antitrypsin
263. Serum immunoreactive trypsin level
264. Serum A1 - acid glycoprotein
265. Serum beta 2 microglobulin level
266. Serum pseudocholinesterase
267. Serum cholinesterase level
268. Enzymes/specific proteins NOS
269. Plasma amylase level
270. Serum haptoglobin screen

271. Serum lipase
272. Leucocyte alkaline phosphatase level
273. Plasma beta 2 microglobulin level
274. Serum P1 - phenotype
275. Tartrate labile serum acid phosphatase level
276. Serum glutamate D.H.
277. Faecal trypsin level
278. Serum orosomucoid level
279. Thiopurine methyltransferase enzyme activity
280. Urine: Bence Jones' protein
281. Urine vanillylmandelic acid/creatinine ratio

Miscellaneous

1. Bronchoscopy
2. Colonoscopy
3. Diabetic retinopathy screening
4. ECG ambulatory
5. ECG exercise
6. Electrocardiogram (ECG)
7. Electroencephalography (EEG)
8. Intraocular pressure left
9. Intraocular pressure right
10. Nerve conduction studies
11. Pap smear
12. Spirometry
13. Upper endoscopy
14. Vaginal smear

Text S2 Extended included tests

Temporal change in age and sex-standardised rates were modelled with joinpoint regression [1]. Joinpoint regression models consists of straight lines which are connected by joinpoints - the estimated location of a significant change in the slope of the trend line. The joinpoint programme starts from a null hypothesis model of zero joinpoints and tests whether the alternative hypothesis of the maximum number of joinpoints specified for the model has a statistically significant lower sum of residual squares [2,3]. Permutation tests are applied sequentially until a model of best fit is reached [3]. Because multiple tests are performed, the significance level of each test is adjusted to control the overall type I error at specified a level (0.05) [2]. The maximum number of joinpoints is dependent upon the number of observations in the model, for our model a maximum of 2 joinpoints was allowed, as recommended [2].

Locations of a significant change in temporal slope were identified (a 'joinpoint') and the annual percentage change (APC) of the slope between joinpoints was determined. Thus, our calculated APC represent the annual percentage change for the years between joinpoints. Calculation of the AAPC allows for comparison of tests over the same time interval and for comparison between time intervals. To determine the annual percentage change over the entire study period (2000/1 to 2015/16) and over the post-QOF period (2004/5 to 2015/16) we calculated the average annual percentage change. The AAPC is computed as a weighted average of the APCs within the joinpoint model, with the weights equal to the length of the respective APC intervals. It is thus valid even if the joinpoint model indicates that there were changes in trend over the interval [4].

To examine the temporal change in utilisation of specific tests, we selected 44 specific tests (28 laboratory, 11 imaging and five other, miscellaneous tests (supplementary file). Twenty-five tests were selected based on the following criteria: 1. Specific guidance on their use in primary care is stated in one or more of the following guidelines/frameworks: Quality Outcomes Framework (QOF), National Institute of Health and Clinical Excellence (NICE), Choosing Wisely or NICE Do Not Do guidelines. 2. They were one of the two most frequent laboratory or imaging tests ordered from Oxfordshire primary care (data attained directly from Oxford University Hospital (OUH)). This list of tests was discussed and refined in consultation with our patient and public involvement group and agreed upon by all authors.

A further 19 tests were identified as part of data cleaning and in consultation with GPs as tests that are typically ordered as one test but return many results. For instance, a liver function test returns results for Alanine aminotransferase (ALT), Aspartarte aminotransferase (AST), Alkaline

phosphatase (ALP) and Gamma glutamyl transferase (GGT) amongst others, all of which have individual Read codes and are recorded separately. Selection of these additional specific tests occurred before data analysis. The full codes are listed above in Supplementary Text S1.

Originally, we were going to exclude tests unlikely to be ordered by GPs. These tests were: Uric acid blood level, serum chloride, serum bicarbonate, anion gap, blood gases, ascitic fluid examination, Schilling test. Upon further expert General Practitioner review, before data was analysed, it was decided to include these tests in the temporal analysis of total tests. Expert GP review concluded that it is likely or at least plausible that these tests are ordered by UK General Practitioner and exclusion of these tests would be inappropriate. It was also stated that given the tiny proportion of total test use made up by the above tests, inclusion of these tests in the total test analysis is very unlikely to affect the results. All clinicians (JOS, FDRH, CS, PL, BG, JA and CH) in the author group discussed and agreed on the above stated process to select the included tests.

Text S3: Extended Discussion

We have taken many measures to ensure that the consistency of coding throughout the study period is consistent. As stated in the manuscript, the CPRD only includes General Practices with robust, up-to-date and valid data [5]. We also considered ordering of the test from two perspectives: a record of the test having been ordered and the result of the test (for instance, via a letter or numerical results). Letter correspondence is often incorporated into CPRD, with around 90% compliance [6,7]. We also believe the following points further support the validity of coding.

Significant clinical event coding. For General Practices to be included in the CPRD, they must report 'significant clinical events' [8,9], including test ordering.

Secondary care data contamination. The CPRD contains primary care data, with additional linkage to secondary care if requested (we did not attain linked secondary data as it was beyond the aim of our study). Thus, given the quality and validation of CPRD data, it is unlikely, but possible, that some of our test ordering we present reflects tests ordered by secondary care clinicians. Assessment of the temporal trends in two specific tests (Knee MRI and Brain MRI) suggests there is little, if any, secondary care contamination in our data. The provision of direct access MRI has been inconsistent around the UK [10], but substantial national effort to increase direct access MRIs for GP occurred around 2005/6 [11,12], these changes are reflected in the substantial increase in knee and brain MRIs around the same time. The rate of Knee, and Brain MRIs from our results are very low before 2005 (the least, and 3rd least utilised tests respectively). It should be noted that the other MRI test in our analysis (Lumbar spine) has been available direct access for GPs since the mid to late 90's [13].

Independent validation of test codes in CPRD. A systematic review reports that *all* abnormal test results are recorded accurately in CPRD [14]. A separate study [9] reports that from 2000 the proportion of abnormal results, out of the total number of tests ordered, has remained relatively constant in CPRD (28.7% from 2000-2004 and 27.0% from 2005 onwards). These results suggest that the coding of all test results (normal and abnormal results) has been consistent from 2000.

Decreasing use of some specific tests before 2004 (QOF introduction). The results from our analysis of 44 specific tests support the conclusion that coding before 2004/5 was valid. We were concerned that test use coding before 2004 would be inconsistent. We were particularly concerned that not all tests that were ordered, were coded. With the advent of electronic ordering, we were less concerned – as this coding then became automated - and we thus conducted a sensitivity analysis when 90% of general practices were electronically ordering tests (after 2004). Nevertheless, our results from three tests (urine drug monitoring, vaginal swabbing, and lumbar spine radiography) add weight to the conclusion that coding was valid before 2004. The use of these three tests fell significantly in use from 2000 to 2003/4. If the data were not coded appropriately, and thus tests were missed before QOF, we would not have anticipated any reductions in use for any test. It is of further encouragement that we noted reductions in the use across all three test types (laboratory, imaging, and miscellaneous).

References

1 Institute National Cancer. Statistical Methodology and Applications Branch, Surveillance Research Program. Joinpoint user's guide 4.5.0.1.

2 National Cancer Institute. Joinpoint Help Manual, Version 4.5.0.1. 2015.http://surveillance.cancer.gov/joinpoint/Joinpoint_Help_4.2.0.0.pdf (accessed 5 Jan 2018).

3 Kim H-J, Fay MP, Feuer EJ, *et al.* Permutation tests for joipoint regression with applications to cancer rates. *Stat Med* 2000;**19**:335–51. doi:10.1002/(SICI)1097-0258(20000215)19:33.3.CO;2-Q

4 Clegg L, Hankey B, Tiwari R, *et al.* Estimating average annual per cent change in trend analysis. *Stat Med* 2009;**28**:3670–3682. doi:10.1002/sim.3733

5 Herrett E, Gallagher AM, Bhaskaran K, *et al.* Data Resource Profile: Clinical Practice Research Datalink (CPRD). *Int J Epidemiol* 2015;**44**:827–36. doi:10.1093/ije/dyv098

6 Khan NF, Harrison SE, Rose PW. Validity of diagnostic coding within the General Practice Research Database: A systematic review. *Br J Gen Pract* 2010;**60**:199–206. doi:10.3399/bjgp10X483562

7 Jick H, Jick SS, Derby LE. Validation of information recorded on general practitioner based computerised data resource in the United Kingdom. *BMJ* 1991;**302**:766–8. doi:10.1136/bmj.302.6779.766

8 Medicines and Healthcare Products Regulatory Agency. GPRD recording guidelines for vision users. London: 2004. http://www.vision3.homecall.co.uk/general/GPR Data Recording Guidelines.pdf

9 Busby J, Schroeder K, Woltersdorf W, *et al.* Temporal growth and geographic variation in the use of laboratory tests by NHS general practices: Using routine data to identify research priorities. *Br J Gen Pract* 2013;**63**:256–66. doi:10.3399/bjgp13X665224

10 Gough-Palmer AL, Burnett C, Gedroyc WM. Open access to MRI for general practitioners: 12 years' experience at one institution - A retrospective analysis. *Br J Radiol* 2009;**82**:687–90. doi:10.1259/bjr/88267089

11 Barrett A. Waiting times for scans to decrease, vows Department of Health. *BMJ* 2005;:331:256.

12 Department of health. Best research for best health: a new national health research strategy. 2006;**6**:435–7.https://www.gov.uk/government/uploads/system/uploads/attachment_data/file/136578/dh_4127152.pdf

13 Chawda S, Watura R, Lloyd DC. Magnetic resonance imaging of the lumbar spine: direct access for general practitioners. *Br J Genera* 1997;**47**:575–6.http://ovidsp.ovid.com/ovidweb.cgi?T=JS&PAGE=reference&D=med4&NEWS=N&AN=9406494

14 Herrett E, Thomas SL, Schoonen WM, *et al.* Validation and validity of diagnoses in the General Practice Research Database: A systematic review. *Br J Clin Pharmacol* 2010;**69**:4–14. doi:10.1111/j.1365-2125.2009.03537.x

Table S4 Age and Sex Adjusted rates of test use per 10,000 person-years, stratified by sex

	**2000/1**	**2001/2**	**2002/3**	**2003/4**	**2004/5**	**2005/6**	**2006/7**	**2007/8**	**2008/9**	**2009/10**	**2010/11**	**2011/12**	**2012/13**	**2013/14**	**2014/15**	**2015/16**	**2000/1 to 2015/16 (95%CI)**	**2004/5 to 2015/16: (95%CI)**
Female	9135.6	11065.7	12803.5	15779.6	18455.2	20702.0	22195.4	23005.9	24688.1	25592.5	26196.4	27304.3	27795.6	29224.1	29096.6	29739.1	8.3% (7.5% to 9.1%)	4.3% (3.5% to 5.1%)
Male	5733.4	7095.9	8315.5	10389.3	12461.6	13891.2	14917.3	15474.1	16565.2	17209.9	17576.3	18220.8	18601.4	19489.4	19073.7	19527.9	8.7% (7.7% to 9.7%)	4.2% (3.2% to 5.1%)

Table S5 Age and sex adjusted rates of test use per 10,000, by test type

Test type	2000/1	2001/2	2002/3	2003/4	2004/5	2005/6	2006/7	2007/8	2008/9	2009/10	2010/11	2011/12	2012/13	2013/14	2014/15	2015/14
Laboratory	13091.2	16196.9	18893.7	23533.8	27898.3	31306.2	33609.1	34840.1	37360.2	38757.5	39633.6	41256.9	41953.7	44234.9	43725.2	44847.0
Imaging	1035.5	1084.3	1189.5	1351.4	1449.0	1648.0	1762.1	1810.0	1940.2	2077.2	2113.3	2181.8	2287.4	2277.5	2271.4	2259.1
Miscellaneous	716.1	843.5	984.1	1222.7	1501.5	1553.7	1639.2	1703.3	1776.2	1745.4	1758.6	1755.2	1786.4	1796.9	1734.3	1696.2

Table S6 Changes in the proportion of tests ordered for patients that received many tests

Annual number of tests received per patient	2000/1	2004/5	2015/16	Absolute percentage change: 2000/1 to 2015/16 (95%CI)	p value	Absolute percentage change: 2004/5 to 2015/16 (95%CI)	p value
1	35.8%	24.2%	19.5%	-16.4% (-16.3% to -16.5%)	p<0.0001	-4.8% (-4.7% to -4.9%)	p<0.0001
2	15.2%	11.6%	9.0%	-6.2% (-6.1% to -6.3%)	p<0.0001	-2.6% (-2.55% to -2.7%)	p<0.0001
3	9.1%	7.7%	5.6%	-3.5% (-3.4% to -3.6%)	p<0.0001	-2.0% (-1.99% to -2.1%)	p<0.0001
4	7.0%	6.4%	4.7%	-2.3% (-2.27% to -2.4%)	p<0.0001	-1.78% (-1.74% to -1.82%)	p<0.0001
5	5.8%	5.9%	4.5%	-1.35% (-1.3% to -1.4%)	p<0.0001	-1.39% (-1.34% to -1.43%)	p<0.0001
6	5.0%	5.8%	4.8%	-0.2% (-0.18% to -0.3%)	p<0.0001	-0.97% (-0.93% to -1.0%)	p<0.0001
7	4.2%	5.4%	5.1%	0.86% (0.82% to 0.9%)	p<0.0001	-0.32% (-0.27% to -0.37%)	p<0.0001
8	3.4%	4.8%	5.0%	1.59% (1.54% to 1.64%)	p<0.0001	0.20% (0.16% to 0.24%)	p<0.0001
9	2.7%	4.1%	4.8%	2.07% (2.03% to 2.1%)	p<0.0001	0.63% (0.59% to 0.67%)	p<0.0001
10	2.2%	3.5%	4.3%	2.18% (2.14% to 2.22%)	p<0.0001	0.88% (0.84% to 0.92%)	p<0.0001
>10	9.5%	21%	33%	23.2% (23.1% to 23.3%)	p<0.0001	12.1% (12.0% to 12.2%)	p<0.0001

Table S7 Age and sex adjusted rates of specific test use per 10,000 person-years

Test	2000	2001	2002	2003	2004	2005	2006	2007	2008	2009	2010	2011	2012	2013	2014	2015
Bone Profile	279.1	367.6	436.7	615.5	781.6	901.3	990.7	1031.8	1104.1	1123.1	1125.2	1189.8	1228.8	1259.1	1266.8	1331.5
Chest x-ray	196.4	217.7	223.4	231.8	246.8	270.1	270.7	278.9	290.2	290.8	300.2	316.2	367.6	349.9	360.3	339.3
Clotting tests	421.9	511.4	571.3	663.0	756.5	873.4	979.1	1073.6	1182.6	1269.3	1351.4	1419.4	1499.2	1614.8	1649.2	1676.7
Colonoscopy	33.9	42.3	49.9	58.1	68.1	77.2	80.2	83.6	91.0	93.1	93.5	96.4	99.1	93.8	90.3	87.9
C-Reactive Protein	91.6	137.2	193.5	260.7	344.2	421.4	470.6	514.6	587.7	635.0	691.9	752.1	788.9	848.4	894.7	924.4
Creatine Kinase	31.6	50.1	70.4	108.8	163.8	201.3	218.3	216.5	207.9	191.2	176.1	134.8	120.7	118.0	106.2	98.1
CT Brain	2.3	3.3	4.7	9.3	13.9	14.1	13.3	12.6	14.5	15.5	15.7	17.5	17.2	17.3	19.3	20.1
CT Pelvis	0.1	0.1	0.1	0.3	0.3	0.3	0.3	0.4	0.4	0.5	1.3	3.7	4.6	5.3	6.4	7.8
DEXA	11.4	18.7	21.7	26.3	27.6	27.2	29.0	31.6	34.7	40.1	38.1	38.8	43.8	48.7	48.8	46.1
Echocardiogram	17.6	25.5	37.5	59.2	77.2	81.9	86.5	88.9	94.1	95.0	93.0	93.7	91.4	96.2	90.2	91.0
Erythrocyte sedimentation rate	501.1	591.4	642.8	752.0	820.4	898.6	916.8	938.4	991.3	1016.8	1015.2	1050.5	1048.1	1059.6	1031.6	1027.2
Female Sex Hormones	68.3	77.4	83.3	95.6	102.6	112.1	114.6	113.3	121.8	123.7	127.1	135.5	130.0	142.2	140.4	140.5
Ferritin	68.6	96.3	126.3	176.8	215.1	267.3	307.8	362.5	437.0	500.2	565.9	636.7	698.2	792.4	864.1	930.6
Folate	64.5	88.0	107.7	144.4	178.6	209.6	235.8	267.9	322.1	381.2	449.5	513.1	586.8	650.7	705.4	780.7
Full Blood Count	1607.7	1920.7	2159.7	2507.4	2775.3	3077.1	3249.1	3383.3	3632.0	3736.9	3897.8	4073.9	4119.7	4304.5	4270.1	4272.1
Glucose	961.9	1240.1	1490.9	1838.3	2046.9	2234.7	2312.0	2325.2	2480.7	2506.5	2524.9	2562.8	2463.9	2373.7	2068.1	1887.4
HbA1c	366.8	434.8	517.5	620.0	713.6	760.8	773.7	794.4	826.9	860.3	908.5	955.7	1100.3	1380.0	1514.1	1711.1
Iron	16.7	20.7	22.6	29.1	37.5	45.1	52.8	61.7	76.3	83.6	97.3	99.3	109.2	120.5	133.9	166.3
Lipids	933.1	1174.2	1425.4	1824.3	2233.8	2401.9	2519.1	2457.5	2555.3	2545.2	2510.8	2549.7	2574.1	2706.0	2457.0	2434.2
Liver Function Tests	977.7	1339.8	1651.3	2146.6	2609.9	2988.9	3229.2	3291.5	3495.9	3558.8	3650.1	3790.1	3844.3	4024.8	3940.4	4002.8
MRI Brain	0.1	0.1	0.1	0.1	0.3	1.1	1.6	4.3	5.9	7.0	8.3	9.6	9.4	10.5	11.5	11.8

MRI Knee	0.0	0.0	0.0	0.0	0.0	0.0	0.1	2.9	4.7	5.9	7.0	7.7	7.7	8.6	8.4	9.1
MRI Lumbar Spine	7.4	9.9	12.4	17.4	24.1	32.5	36.8	41.9	48.1	54.9	57.0	57.5	57.8	60.9	60.3	60.3
Oestradiol	29.9	35.0	37.8	40.2	42.7	46.5	47.6	47.3	50.9	51.0	50.8	52.2	48.7	49.0	49.1	48.0
Pap Smear	61.1	87.8	96.3	92.5	84.1	107.0	117.9	123.6	116.0	105.2	97.0	88.6	75.0	85.6	82.2	82.6
Progesterone	13.0	15.2	16.9	20.1	23.1	26.2	26.8	26.8	28.9	30.3	32.8	35.9	35.3	35.9	35.7	35.7
Prostate Specific Antigen	102.6	128.4	149.7	187.7	213.0	235.3	248.4	264.4	296.6	311.5	316.8	319.8	321.9	352.6	331.5	326.4
Renal Function	1422.1	1826.8	2188.1	2753.8	3216.9	3571.2	3920.4	3988.6	4178.5	4277.1	4366.6	4527.7	4565.6	4799.9	4758.0	4854.6
Spirometry	418.7	453.6	518.3	675.6	880.7	848.8	857.1	832.7	847.8	809.3	805.0	789.2	817.5	781.7	740.9	700.7
Testosterone	15.6	20.5	24.1	28.8	30.3	34.4	35.8	38.8	45.2	53.1	57.9	63.3	65.0	73.3	72.3	72.5
Thyroid Function Tests	862.0	1068.3	1223.2	1523.6	1755.0	1934.2	1995.1	2032.2	2187.6	2248.8	2279.7	2372.2	2389.2	2498.3	2393.1	2424.0
Troponin	1.0	2.5	4.5	9.0	13.6	17.2	18.3	17.8	18.1	18.0	19.8	21.1	19.2	19.5	17.7	17.1
Upper Endoscopy	41.1	52.3	61.1	66.2	68.0	70.1	72.6	74.5	83.1	84.7	88.9	88.2	91.1	91.4	91.2	96.3
Urine Albumin Creatinine ratio	6.5	7.9	6.0	42.1	143.0	179.7	225.4	242.5	276.1	481.1	497.3	544.5	558.9	680.2	675.1	564.4
Urine Albumin	13.1	23.1	46.8	116.5	205.5	239.3	253.7	265.3	282.7	367.6	336.2	337.4	318.8	310.9	225.8	168.4
Urine Dipstick	1023.9	1157.0	1293.5	1445.8	1448.3	1536.5	1674.3	1712.1	1781.3	1936.4	1888.0	1904.7	1841.8	1855.8	1690.9	1548.2
Urine MCS	476.1	552.8	615.9	682.6	712.6	783.2	866.6	899.0	956.5	975.6	949.9	920.8	918.3	962.5	964.8	924.4
Urine non-illicit drugs	9.9	9.5	7.2	7.3	7.5	8.4	8.8	9.5	11.2	15.9	16.9	18.2	19.8	16.4	13.5	12.9
US Pelvis	51.3	58.9	63.4	69.3	75.4	83.1	84.3	91.2	98.4	115.9	124.8	135.5	134.5	136.9	128.5	119.3
Vaginal Swab	133.9	148.5	150.9	129.2	109.7	111.4	106.5	102.2	108.4	105.9	94.7	90.5	79.8	76.0	63.3	51.0
Vitamin B12	75.4	98.4	117.4	157.6	191.6	225.3	252.7	286.6	343.8	401.1	470.1	534.2	609.8	676.9	733.0	811.7
Vitamin D	0.3	0.5	0.9	1.4	2.1	2.8	5.5	8.0	13.8	26.3	52.3	81.7	135.0	191.4	204.2	181.9
X-ray Knee	46.1	49.7	50.8	56.5	61.1	69.1	74.0	79.7	86.3	90.1	93.9	96.8	95.4	100.0	101.1	103.6
X-ray Lumber Spine	56.1	54.0	52.7	54.0	56.9	61.1	60.3	60.3	62.4	59.5	56.8	56.0	54.3	56.2	53.8	54.3

Table S8 Average annual percentage increase in specific test use

	2000/1 to 2015/16	Rank %increase 2000/1 to 2015/16	2004/5 to 2015/16: Post QOF	Rank %increase 2004/5 to 2015/16
MRI Knee	69.0% (38.4% to 106.5%)	1	46.5% (27.5% to 68.2%)	2
Vitamin D	53.7% (50.2% to 57.3%)	2	49.6% (45.9% to 53.3%)	1
MRI Brain	47.3% (39.5% to 55.5%)	3	33.8% (28.7% to 39.2%)	4
Urine Albumin Creatinine ratio	44.6% (19.8% to 74.6%)	4	15.6% (8.8% to 22.8%)	5
CT Pelvis	28.4% (16.4% to 41.7%)	5	37.1% (21.7% to 54.4%)	3
Urine Albumin	20.3% (12.3% to 28.7%)	6	-1.0% (-4.8% to 3.0%)	40
Ferritin	19.2% (17.6% to 20.9%)	7	14.4% (13.6% to 15.2%)	7
Troponin	18.8% (16.1% to 21.6%)	8	0.8% (-0.6% to 2.3%)	35
Folate	17.7% (16.5% to 19.0%)	9	14.4% (13.5% to 15.2%)	8
Vitamin B12	17.0% (15.9% to 18.2%)	10	14.0% (13.2% to 14.8%)	9
C-Reactive Protein	16.5% (15.4% to 17.6%)	11	9.4% (8.5% to 10.3%)	10
Iron	16.4% (14.7% to 18.2%)	12	14.7% (12.7% to 16.8%)	6
CT Brain	16.3% (12.4% to 20.3%)	13	3.3% (-0.3% to 7.1%)	26
MRI Lumbar Spine	15.4% (14.3% to 16.6%)	14	9.0% (8.0% to 10.1%)	11
Echocardiogram	11.4% (10.1% to 12.8%)	15	1.4% (0.2% to 2.5%)	33
HbA1c	11.0% (9.5% to 12.4%)	16	8.6% (7.3% to 9.8%)	13
Bone Profile	10.9% (10.1% to 11.7%)	17	5.4% (4.9% to 5.8%)	16
DEXA	10.0% (5.7% to 14.5%)	18	6.0% (5.0% to 7.1%)	15
Testosterone	9.7% (8.2% to 11.2%)	19	8.9% (7.1% to 10.6%)	12

Liver Function Tests	9.5% (8.7% to 10.3%)	20	4.5% (4.1% to 5.0%)	19
Clotting tests	9.3% (8.7% to 9.9%	21	7.6% (6.9% to 8.3%)	14
Renal Function	8.6% (7.4% to 9.9%)	22	4.2% (3.3% to 5.1%)	22
Prostate Specific Antigen	8.1% (6.3% to 9.9%)	23	4.2% (2.6% to 5.8%)	23
Creatine Kinase	7.6% (4.1% to 11.3%)	24	-5.4% (-8.7% to -2.0%)	44
Progesterone	7.1% (6.0% to 8.2%)	25	4.0% (3.0% to 5.0%)	24
Thyroid Function Tests	7.1% (6.1% to 8.1%)	26	2.7% (1.8% to 3.6%)	29
Lipids	6.6% (5.7% to 7.6%)	27	0.2% (-0.8% to 1.1%)	37
Full Blood Count	6.5% (5.8% to 7.2%)	28	4.2% (3.4% to 4.9%)	21
Colonoscopy	6.1% (5.1% to 7.2%)	29	2.3% (1.2% to 3.4%)	31
X-ray Knee	5.9% (5.4% to 6.3%)	30	4.8% (4.3% to 5.3%)	18
Ultrasound Pelvis	5.6% (4.6% to 6.6%)	31	4.5% (3.3% to 5.8%)	20
Upper Endoscopy	5.6% (4.1% to 7.0%)	32	3.1% (2.3% to 3.9%)	28
Female Sex Hormones	5.0% (4.2% to 5.8%)	33	3.2% (2.7% to 3.8%)	27
Erythrocyte sedimentation rate	4.7% (3.9% to 5.4%)	34	2.2% (1.4% to 3.0%)	32
Spirometry	4.7% (3.6% to 5.9%)	35	-1.3% (-2.0% to -0.6%)	41
Glucose	4.5% (3.5% to 5.5%)	36	-0.9% (-1.8% to 0%)	39
Urine MCS	4.3% (3.6% to 5.0%)	37	2.5% (1.9% to 3.1%)	30
Chest x-ray	3.9% (3.3% to 4.5%)	38	3.9% (3.3% to 4.5%)	25
Urine Dipstick	3.1% (1.8% to 4.4%)	39	0.8% (-0.2% to 1.9%)	36
Oestradiol	2.9% (1.6% to 4.1%)	40	1.0% (-0.4% to 2.5%)	34
Urine non-illicit drugs	1.4% (-2.3% to 5.2%)	41	4.9% (1.0% to 8.9%)	17
Pap Smear	-0.1% (-2.5% to 2.3%)	42	-2.6% (-4.9% to -0.2%)	42
X-ray Lumbar Spine	-0.3% (-1.7% to 1.1%)	43	-0.6% (-1.4% to 0.2%)	38

Vaginal Swab	-5.2% (-6.3% to -4.1%)	44	-5.2% (-6.3% to -4.1%)	43

Table S9 A comparison of tests included and not included in the Quality Outcomes Framework

	Median AAPC 2000/1 to 2015/16 (IQR)	Median absolute change in rate of test use from 2000/1 to 2015/16 per 10,000 person-years
Tests in the Quality Outcomes Framework	9.8% (6.6% to 12.8%)	830.9 (250.3 to 1516.3)
Tests not in the Quality Outcomes Framework	7.4% (4.6% to 16.4%)	57.2 (17.4 to 192.2)

Table S10 Tests included and not included the Quality Outcomes Framework

Test	QOF?	Average Annual Percentage increase 2000/1 to 2015/16	Absolute change 2000/1 to 2015/16 per 10,000 person-years	QOF Details
Bone Profile	Yes	10.90%	1052.4	QOF for Dementia: NM09: The percentage of patients with a new diagnosis of dementia recorded in the preceding 1 April to 31 March with a record of FBC, calcium, glucose, renal and liver function, thyroid function tests, serum vitamin B12 and folate levels recorded between 6 months before or after entering on to the register (NM72: same as above, but 'up to 12 months before entering on to the register')
Chest x-ray	No	3.90%	142.9	
Clotting tests	No	9.30%	1254.8	
Colonoscopy	No	6.10%	54	
C-Reactive Protein	No	16.50%	832.8	
Creatine Kinase	No	7.60%	66.5	
CT Brain	No	16.30%	17.8	
CT Pelvis	No	28.40%	7.7	
DEXA	Yes	10.00%	34.7	* QOF for OP<br>NM30: The percentage of patients aged 50 or over and who have not attained the age of 75, with a record of a fragility fracture on or after 1 April 2012, in whom osteoporosis is confirmed on DXA scan, who are currently treated with an appropriate bone-sparing Agent
Echocardiogram	Yes	11.40%	73.4	* QOF for CHF:<br>NM116: The percentage of patients with a diagnosis of heart failure (diagnosed on or after 1 April 2006) which has been confirmed by an echocardiogram or by specialist assessment 3 months before or 12 months after entering on to the register
Erythrocyte sedimentation rate	No	4.70%	526.1	

Female Sex Hormones	No	5.00%	72.2	
Ferritin	No	19.20%	862	
Folate	Yes	17.70%	716.2	* QOF for Dementia:<br>NM09: The percentage of patients with a new diagnosis of dementia recorded in the preceding 1 April to 31 March with a record of FBC, calcium, glucose, renal and liver function, thyroid function tests, serum vitamin B12 and folate levels recorded between 6 months before or after entering on to the register (NM72: same as above, but 'up to 12 months before entering on to the register')
Full Blood Count	Yes	6.50%	2664.4	* QOF for Dementia:<br>NM09: The percentage of patients with a new diagnosis of dementia recorded in the preceding 1 April to 31 March with a record of FBC, calcium, glucose, renal and liver function, thyroid function tests, serum vitamin B12 and folate levels recorded between 6 months before or after entering on to the register (NM72: same as above, but 'up to 12 months before entering on to the register')
Glucose	Yes	4.50%	925.5	* QOF for Dementia:<br>NM09: The percentage of patients with a new diagnosis of dementia recorded in the preceding 1 April to 31 March with a record of FBC, calcium, glucose, renal and liver function, thyroid function tests, serum vitamin B12 and folate levels recorded between 6 months before or after entering on to the register (NM72: same as above, but 'up to 12 months before entering on to the register')<br><br>* QOF for Mental Health<br>NM130: The percentage of patients aged 18 years and over with schizophrenia, bipolar affective disorder and other psychoses who have a record of blood glucose or HbA1c in the preceding 12 months
HbA1c	Yes	11.00%	1344.3	* QOF for Diabetes<br>NM74: The percentage of patients with diabetes who have had the following care processes performed in the preceding 12 months: BMI measurement; BP measurement; HbA1c measurement; Cholesterol measurement; Record of smoking status; Foot examination; Albumin: creatinine ratio; Serum creatinine measurement<br><br>* QOF for Mental Health<br>NM130: The percentage of patients aged 18 years and over with schizophrenia, bipolar affective disorder and other psychoses who have a record of blood glucose or HbA1c in the preceding 12 months
Iron	No	16.40%	149.6	

Lipids	Yes	6.60%	1501.1	*QOF for Coronary Heart Disease :<br>NM118: The percentage of patients with coronary heart disease whose last measured total cholesterol (measured in the preceding 12 months) is 5 mmol/l or less.<br><br>*QOF for Diabetes<br>NM74: The percentage of patients with diabetes who have had the following care processes performed in the preceding 12 months: BMI measurement; BP measurement; HbA1c measurement; Cholesterol measurement; Record of smoking status; Foot examination; Albumin: creatinine ratio; Serum creatinine measurement<br><br>*QOF for Mental Health<br>NM129: The percentage of patients aged 18 and over with schizophrenia, bipolar affective disorder and other psychoses who have a record of total cholesterol: hdl ratio in the preceding 12 months *This is actually Total Cholesterol: HDL ratio<br><br>*QOF for PAD<br>NM35: The percentage of patients with peripheral arterial disease in whom the last measured total cholesterol (measured in preceding 15 months) is 5.0mmol/l or less<br><br>*QOF for Stroke/TIA<br>NM60: The percentage of patients with a stroke shown to be non- haemorrhagic, or a history of TIA whose last measured total cholesterol (measured in the preceding 15 months) is 5mmol/l or less
Liver Function Tests	Yes	9.50%	3025.1	* QOF for Dementia:<br>NM09: The percentage of patients with a new diagnosis of dementia recorded in the preceding 1 April to 31 March with a record of FBC, calcium, glucose, renal and liver function, thyroid function tests, serum vitamin B12 and folate levels recorded between 6 months before or after entering on to the register (NM72: same as above, but 'up to 12 months before entering on to the register')
MRI Brain	No	47.30%	11.7	
MRI Knee	No	69.00%	9.1	
MRI Lumbar Spine	No	15.40%	52.9	
Oestradiol	No	2.90%	18.1	
Pap Smear	Yes	-0.10%	21.5	*Cervical screening<br>CS01 The percentage of women (aged from 25 to 64 in England and Northern Ireland, from 20 to 60 in Scotland<br>and from 20 to 64 in Wales) whose notes record that a cervical screening test has

				been performed in the preceding 5 years
Progesterone	No	7.10%	22.7	
Prostate Specific Antigen	No	8.10%	223.8	
Renal Function	Yes	8.60%	3432.5	* QOF for Dementia:<br>NM09: The percentage of patients with a new diagnosis of dementia recorded in the preceding 1 April to 31 March with a record of FBC, calcium, glucose, renal and liver function, thyroid function tests, serum vitamin B12 and folate levels recorded between 6 months before or after entering on to the register (NM72: same as above, but 'up to 12 months before entering on to the register')
Spirometry	Yes	4.70%	282	* QOF for COPD:<br>NM103: The percentage of patients with COPD (diagnosed on or after 1 April 2011) in whom the diagnosis has been confirmed by post bronchodilator spirometry between 3 months before and 12 months after entering on to the register (Inherited)<br>NM105: The percentage of patients with COPD with a record of FEV1 in the preceding 12 months (Inherited)
Testosterone	No	9.70%	56.9	
Thyroid Function Tests	Yes	7.10%	1562	* QOF for Dementia:<br>NM09: The percentage of patients with a new diagnosis of dementia recorded in the preceding 1 April to 31 March with a record of FBC, calcium, glucose, renal and liver function, thyroid function tests, serum vitamin B12 and folate levels recorded between 6 months before or after entering on to the register (NM72: same as above, but 'up to 12 months before entering on to the register')
Troponin	No	18.80%	16.1	
Upper Endoscopy	No	5.60%	55.2	
Urine Albumin	No	5.60%	557.9	

Urine Albumin Creatinine ratio	Yes	20.30%	155.3	*QOF for Chronic Kidney Disease<br>NM109: The percentage of patients on the CKD register whose notes have a record of a urine albumin:creatinine ratio (or protein:creatinine ratio) test in the preceding 12 months (Inherited)<br><br>*QOF for HTN<br>NM75: The percentage of patients with a new diagnosis of hypertension in the preceding 1st April to 31st March who have a record of urinary albumin: creatinine ratio test in the three months before or after the date of entry to the hypertension register.<br><br>*QOF for Diabetes<br>NM59: The percentage of patients with diabetes who have a record of an albumin:creatinine ratio (ACR) test in the preceding 15 months
Urine Dipstick	Yes	44.60%	524.3	* QOF for HTN<br>-NM76: The percentage of patients with a new diagnosis of hypertension in the preceding 1st April to 31st March who have a record of a test for haematuria in the three months before or after the date of entry to the hypertension register.
Urine MCS	No	3.10%	448.3	
Urine non-illicit drugs	No	4.30%	3	
US Pelvis	No	1.40%	68	
Vaginal Swab	No	-5.20%	-82.9	
Vitamin B12	Yes	17.00%	736.3	* QOF for Dementia:<br>NM09: The percentage of patients with a new diagnosis of dementia recorded in the preceding 1 April to 31 March with a record of FBC, calcium, glucose, renal and liver function, thyroid function tests, serum vitamin B12 and folate levels recorded between 6 months before or after entering on to the register (NM72: same as above, but 'up to 12 months before entering on to the register')
Vitamin D	No	53.70%	181.6	
X-ray Knee	No	5.90%	57.5	
X-ray Lumber Spine	No	-0.30%	-1.8	

Figures S9 Temporal trends in test use stratified by Sex

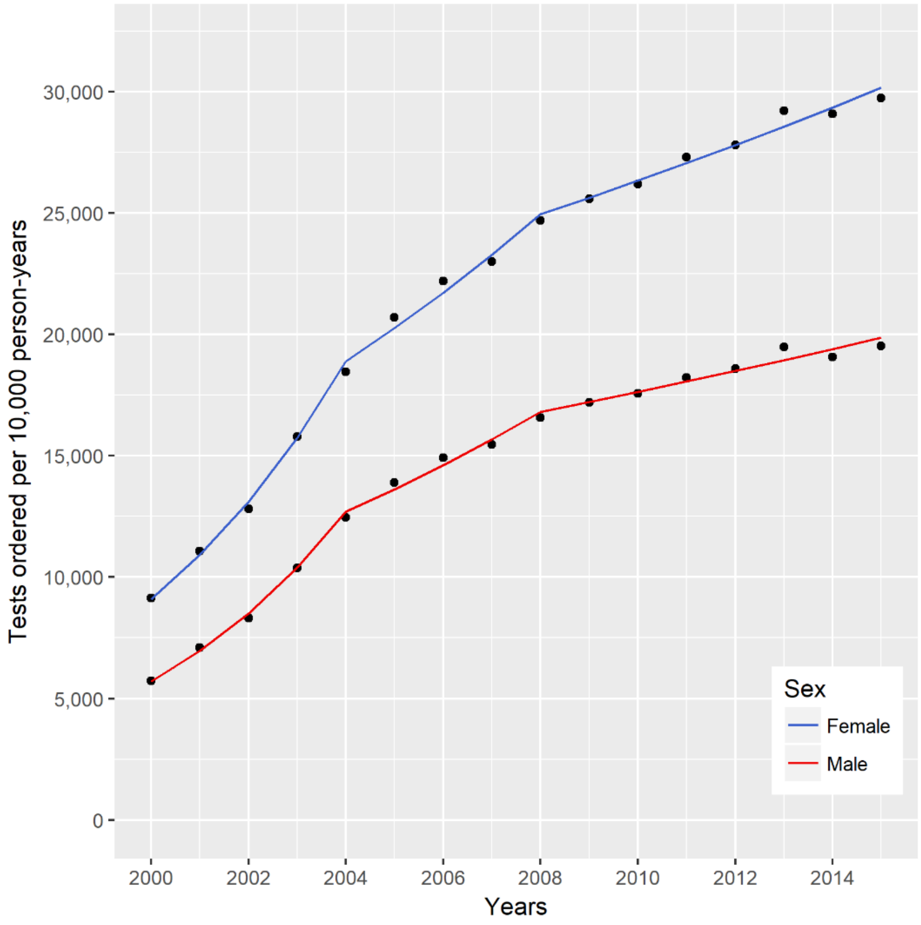

Female: APC: 2000 – 2004: 20.1%, 2004 – 2008: 7.2%, 2008-2015: 2.7%
Male: APC: 2000 – 2004: 22.1%, 2004 – 2008: 7.3%, 2008-2015: 2.4%

Figures S10 AAPC plotted against absolute change in test use per 10,000 person years

This figure has been submitted as a separate supplementary figure

Figures S11 Temporal trends in specific test use

Pattern: Consistent, linear increase

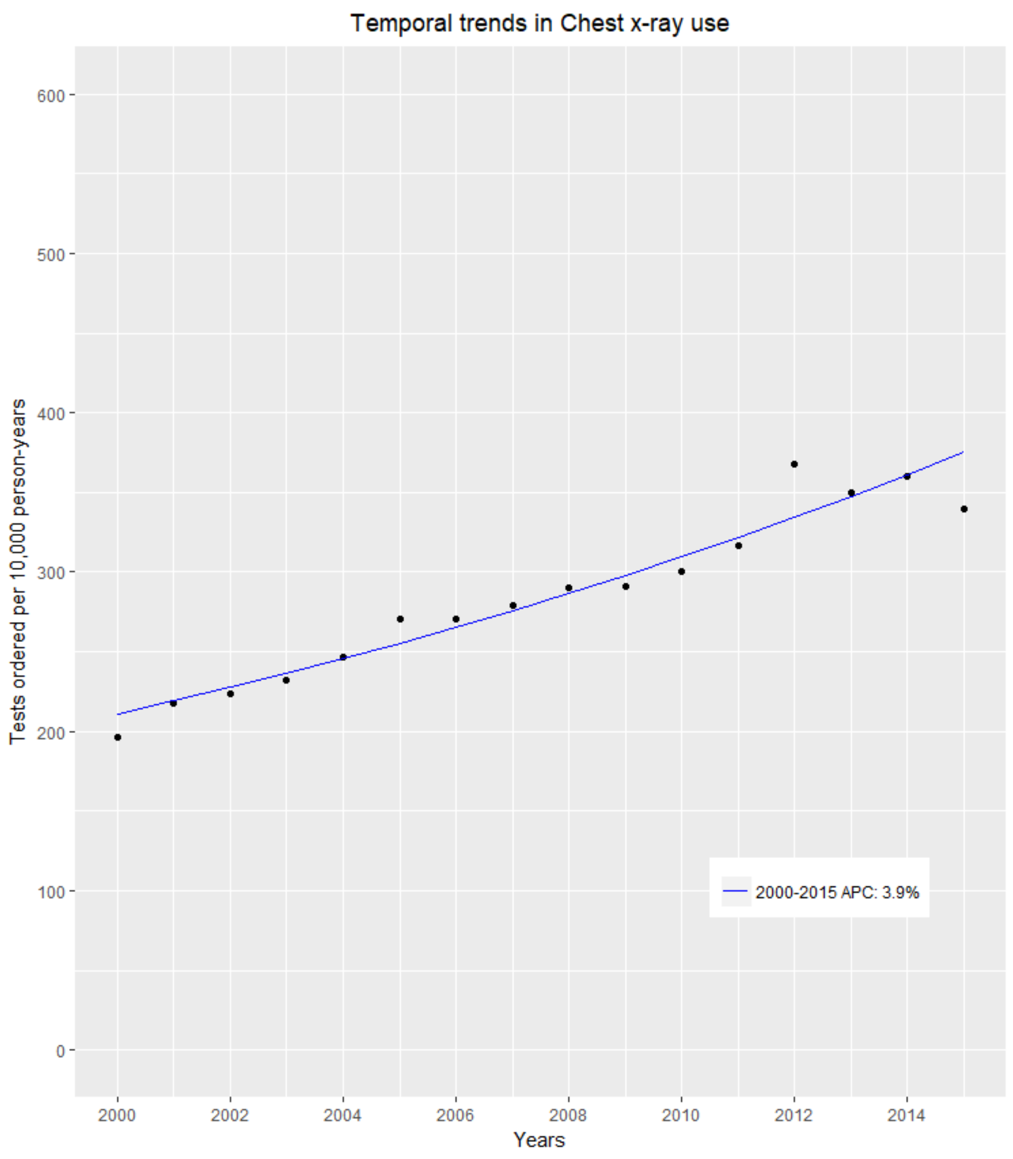

Temporal trends in Chest x-ray use
Tests ordered per 10,000 person-years
600
500
400
300
200
100
0
2000
2002
2004
2006
2008
2010
2012
2014
Years
2000-2015 APC: 3.9%

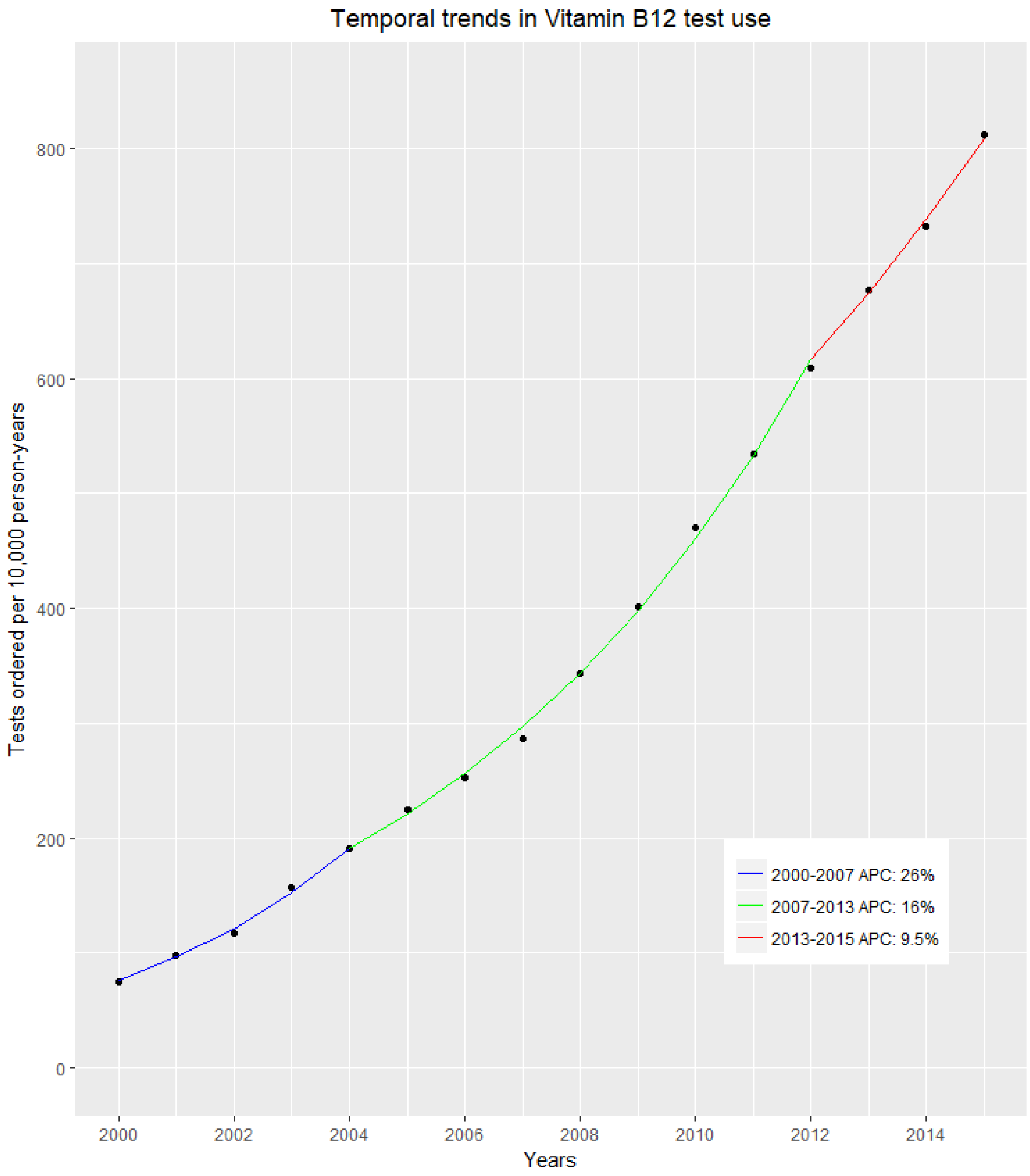

Temporal trends in Vitamin B12 test use
Tests ordered per 10,000 person-years
800
600
400
200
0
2000
2002
2004
2006
2008
2010
2012
2014
Years
2000-2007 APC: 26%
2007-2013 APC: 16%
2013-2015 APC: 9.5%

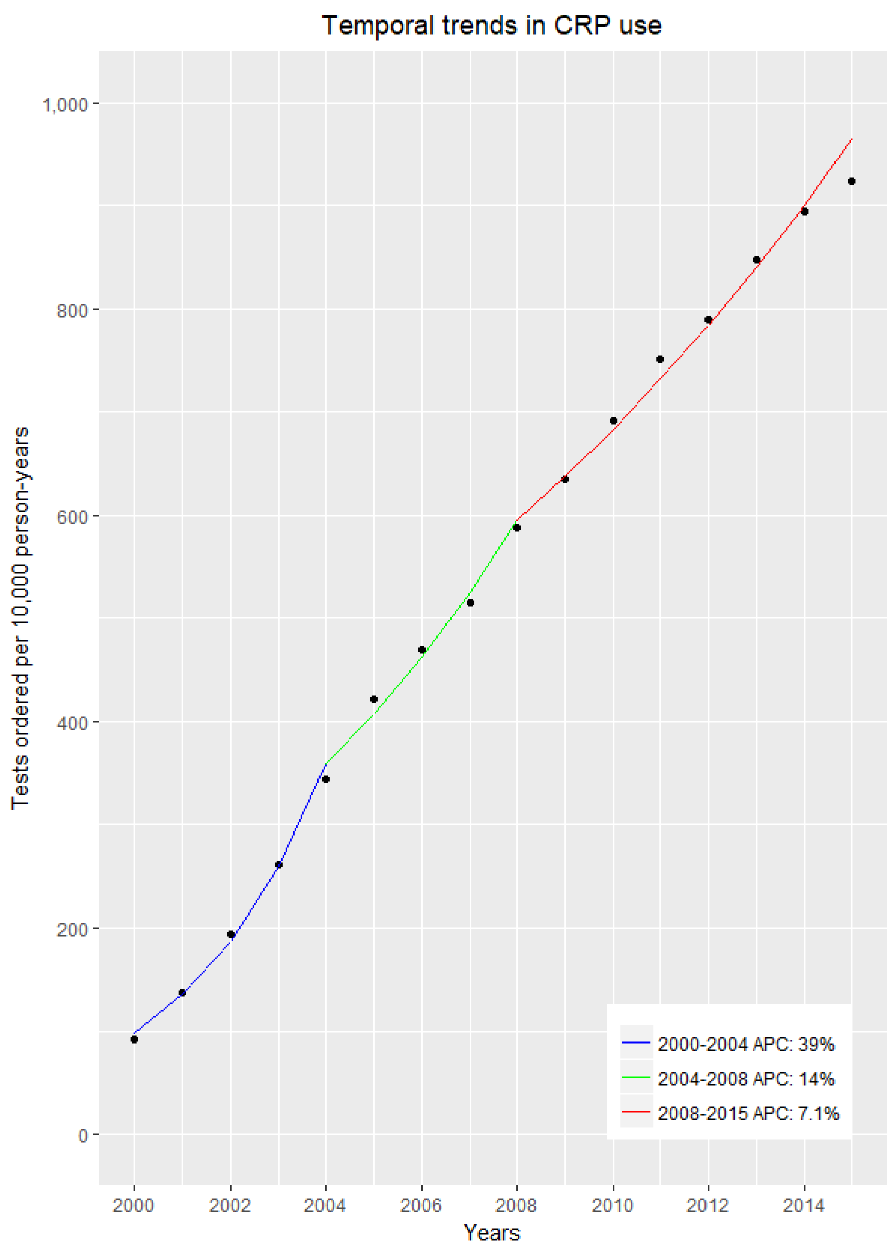

Temporal trends in CRP use
1,000
800
600
400
200
0
Tests ordered per 10,000 person-years
2000
2002
2004
2006
2008
2010
2012
2014
Years
2000-2004 APC: 39%
2004-2008 APC: 14%
2008-2015 APC: 7.1%

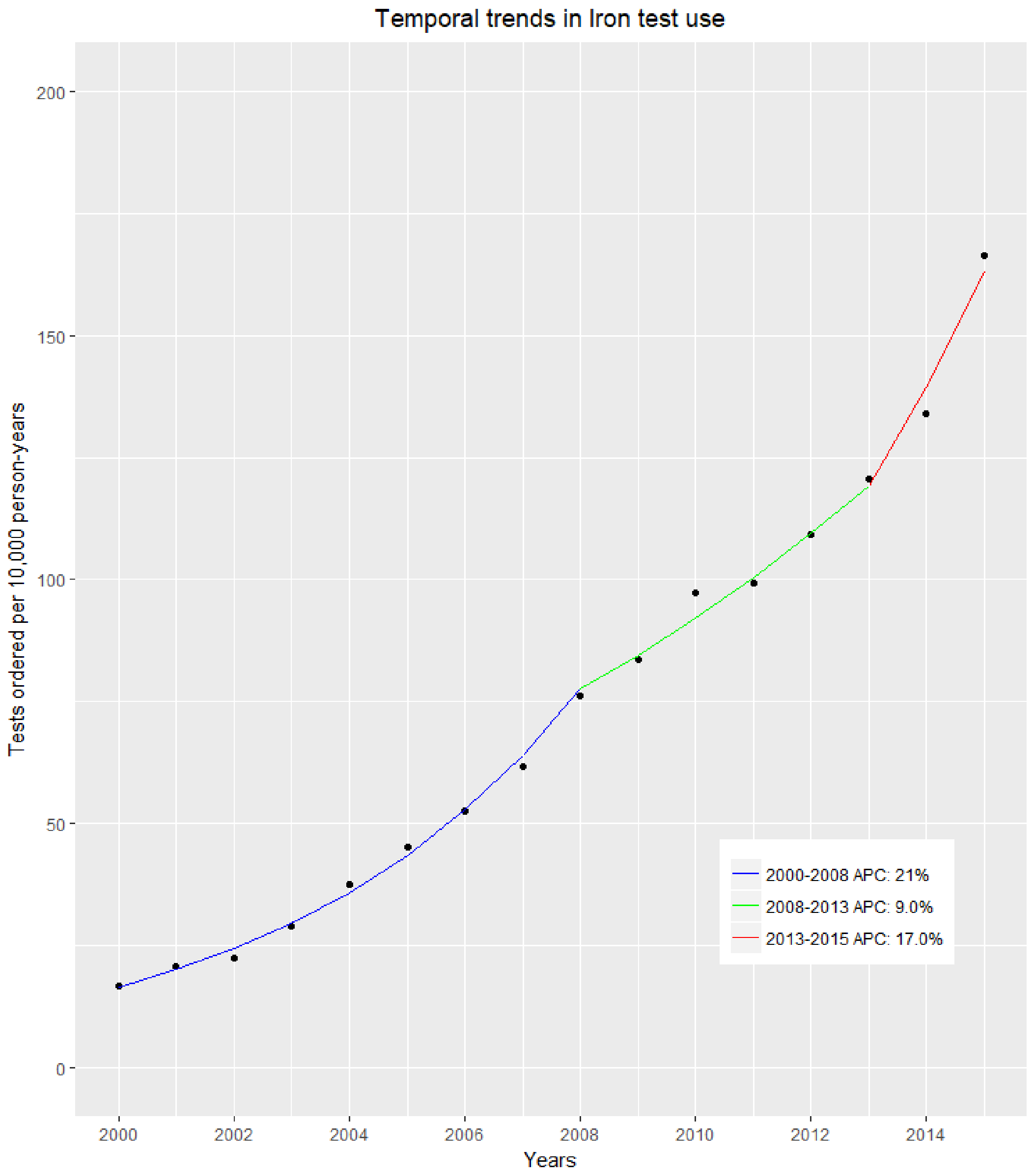

Temporal trends in Iron test use
Tests ordered per 10,000 person-years
200
150
100
50
0
2000
2002
2004
2006
2008
2010
2012
2014
Years
2000-2008 APC: 21%
2008-2013 APC: 9.0%
2013-2015 APC: 17.0%

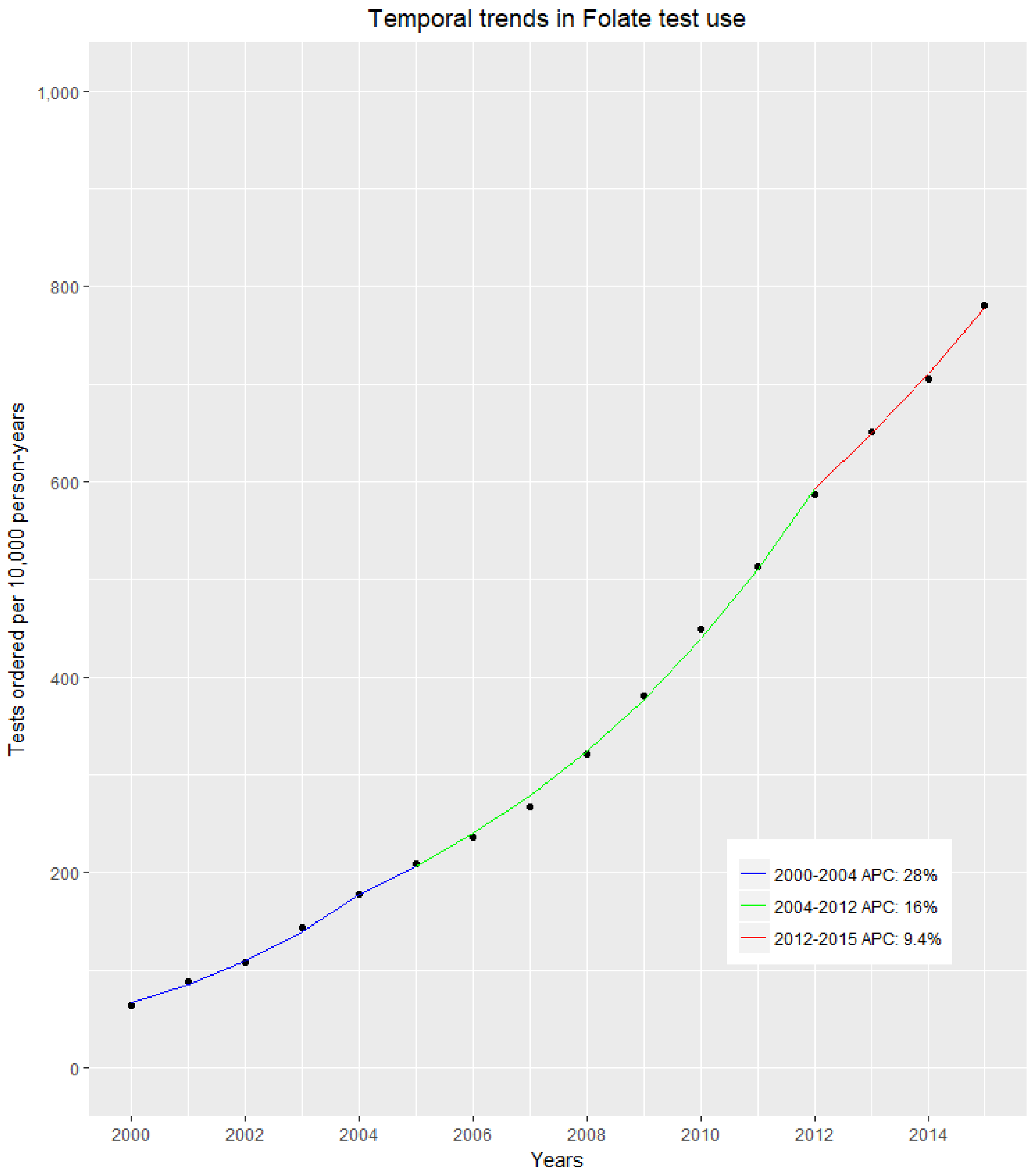

Temporal trends in Folate test use
Tests ordered per 10,000 person-years
1,000
800
600
400
200
0
2000
2002
2004
2006
2008
2010
2012
2014
Years
2000-2004 APC: 28%
2004-2012 APC: 16%
2012-2015 APC: 9.4%

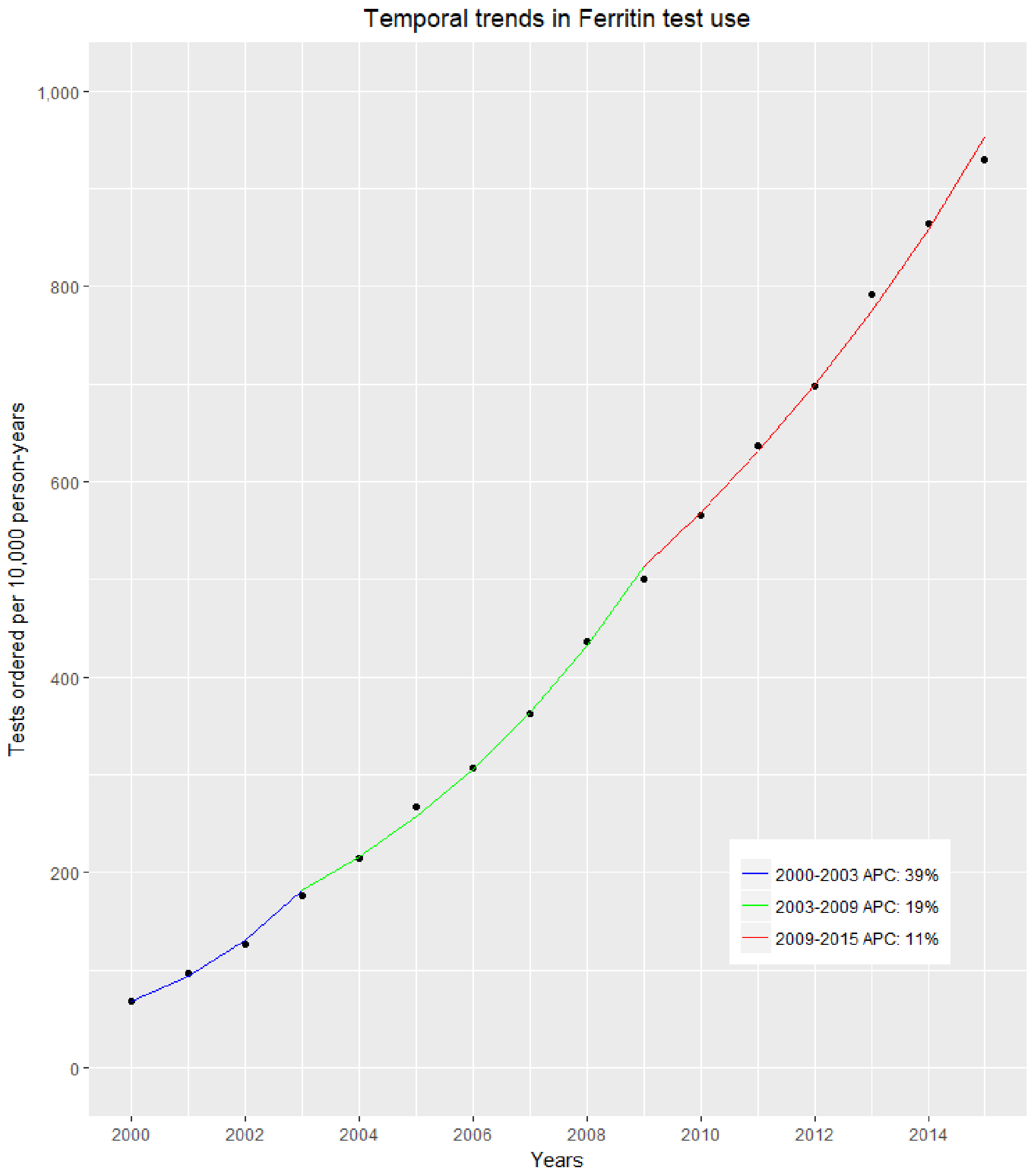
Temporal trends in Ferritin test use
Tests ordered per 10,000 person-years
1,000
800
600
400
200
0
2000
2002
2004
2006
2008
2010
2012
2014
Years
2000-2003 APC: 39%
2003-2009 APC: 19%
2009-2015 APC: 11%

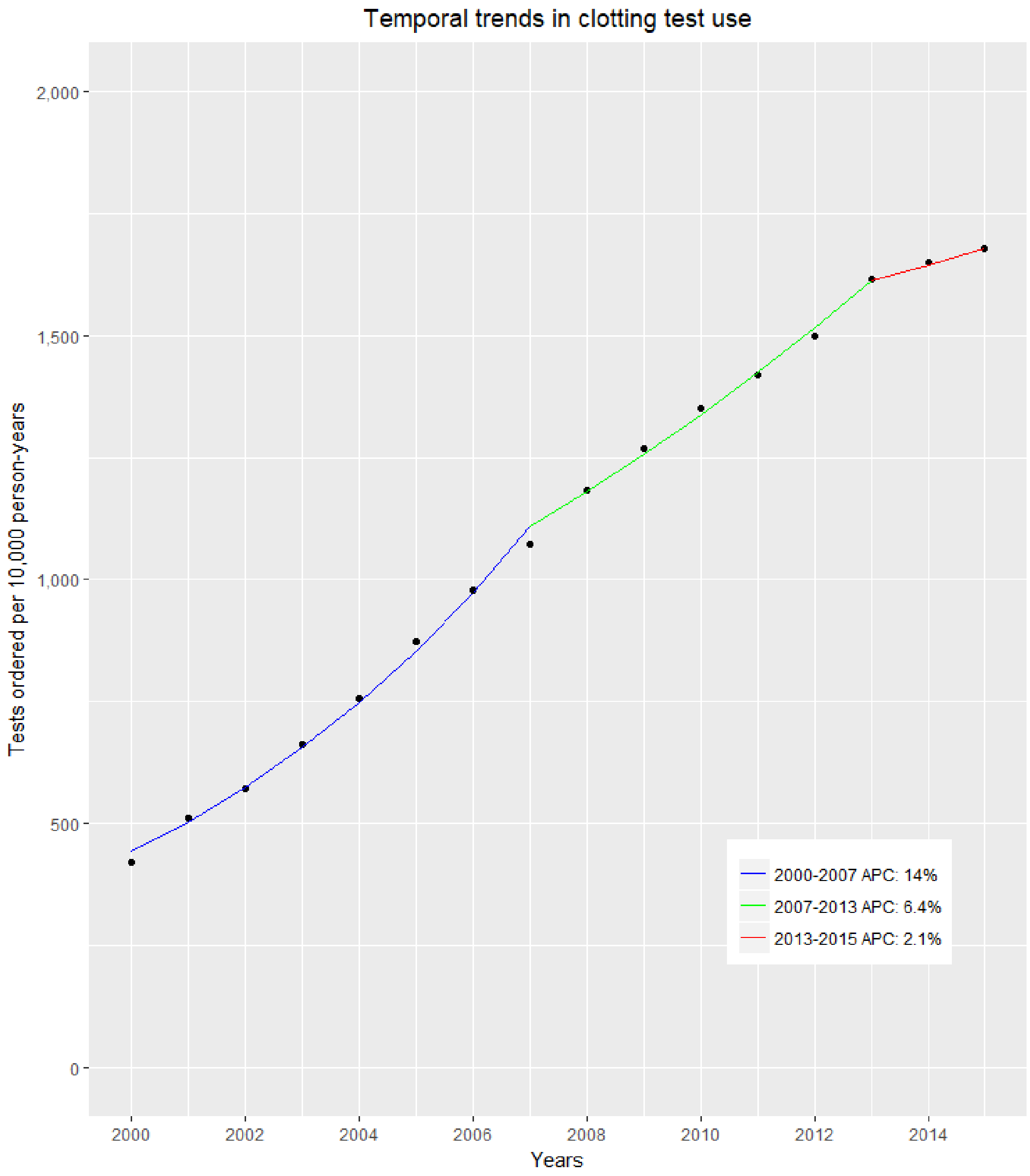
Temporal trends in clotting test use
Tests ordered per 10,000 person-years
2,000
1,500
1,000
500
0
2000
2002
2004
2006
2008
2010
2012
2014
Years
2000-2007 APC: 14%
2007-2013 APC: 6.4%
2013-2015 APC: 2.1%

Pattern: Rapid increase and then increased less quickly

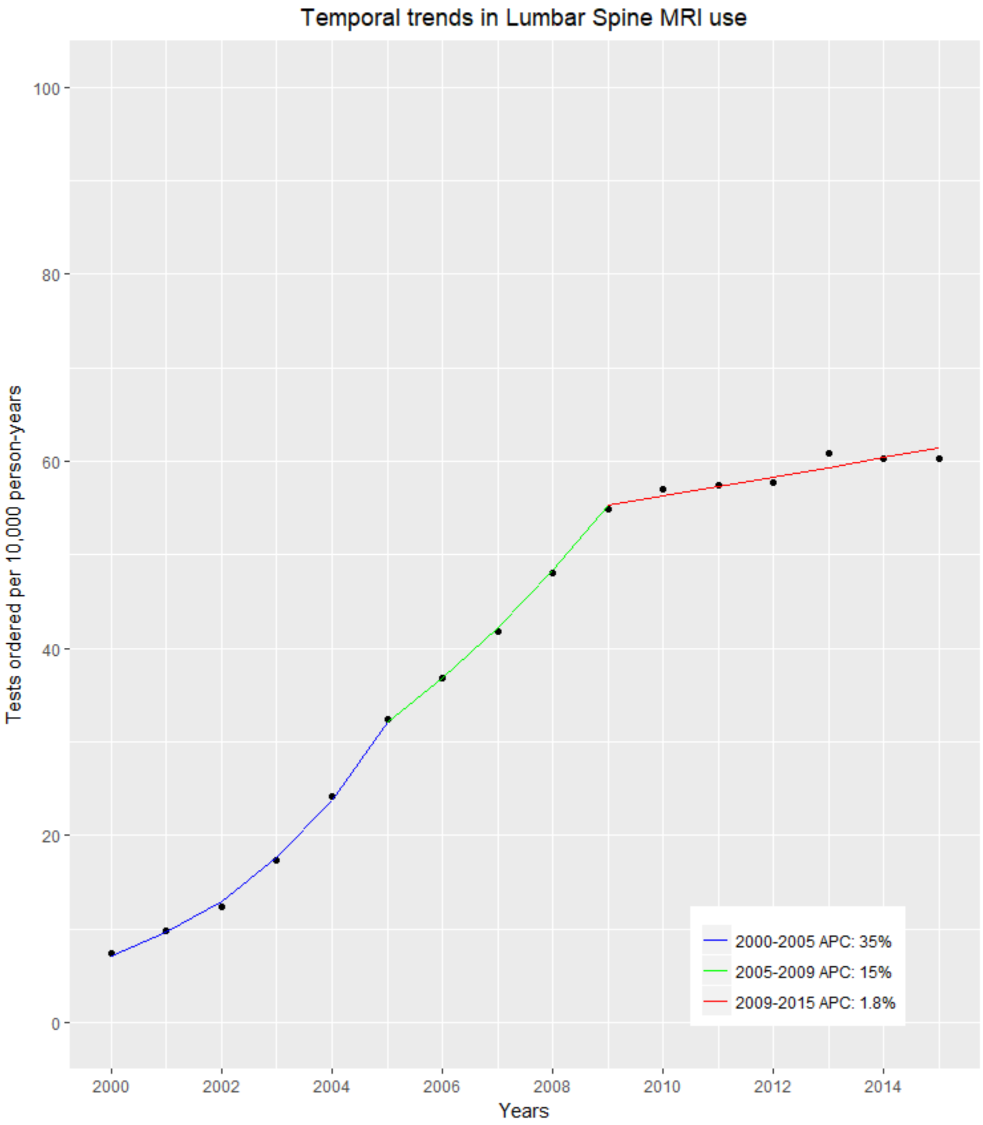

Temporal trends in Lumbar Spine MRI use
Tests ordered per 10,000 person-years
100
80
60
40
20
0
2000
2002
2004
2006
2008
2010
2012
2014
Years
2000-2005 APC: 35%
2005-2009 APC: 15%
2009-2015 APC: 1.8%

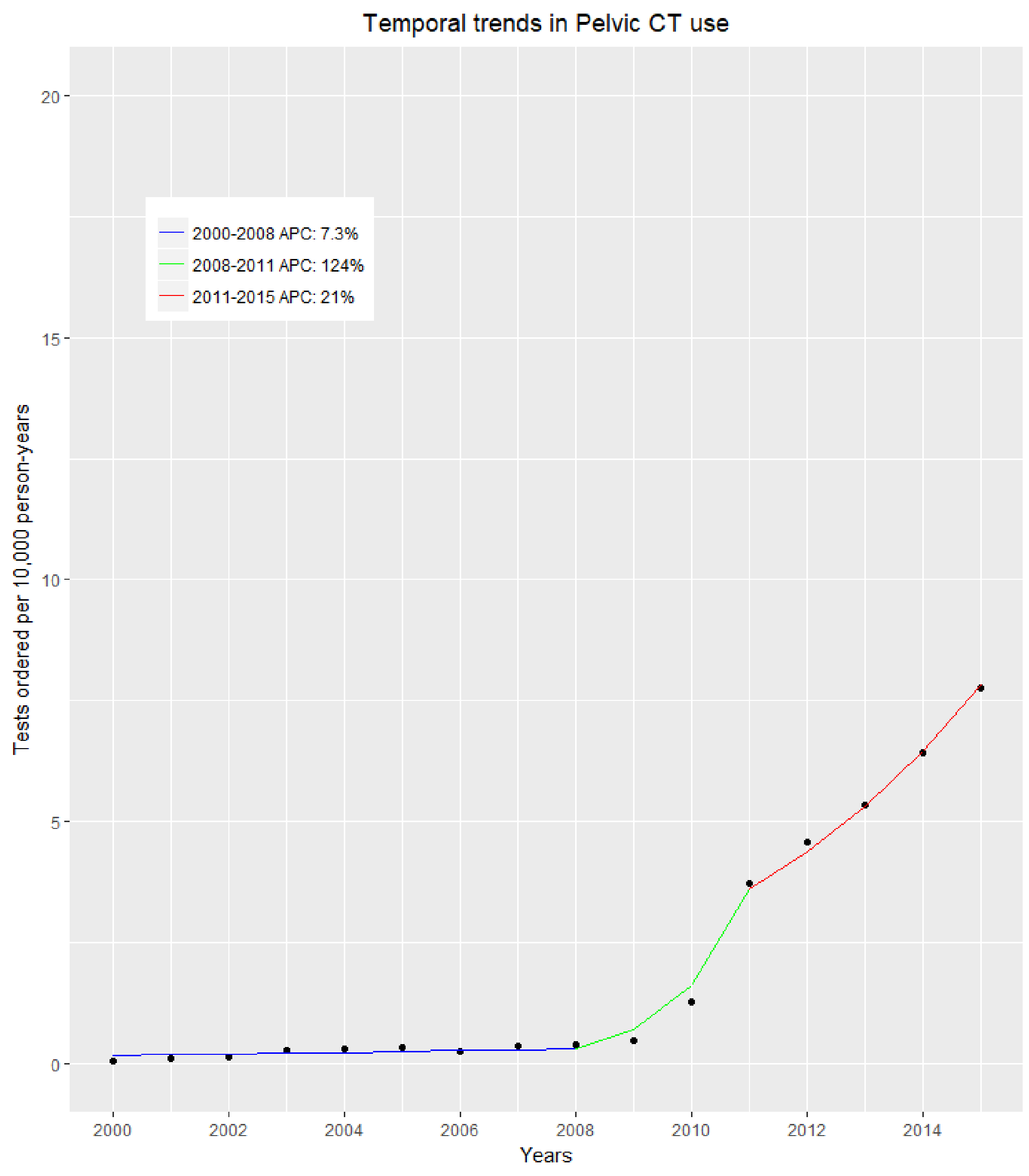

Temporal trends in Pelvic CT use
2000-2008 APC: 7.3%
2008-2011 APC: 124%
2011-2015 APC: 21%
Tests ordered per 10,000 person-years
Years
0
5
10
15
20
2000
2002
2004
2006
2008
2010
2012
2014

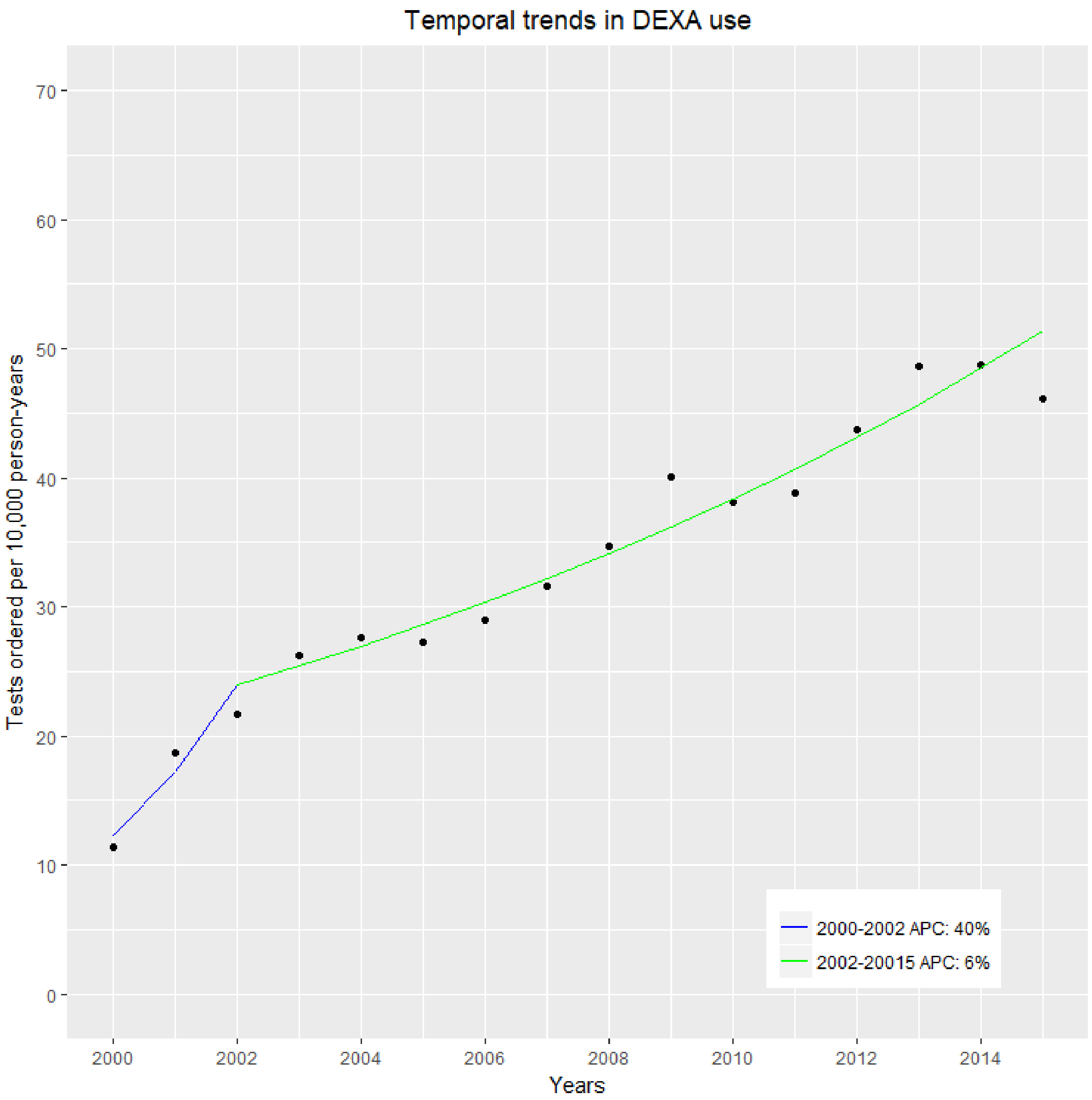
Temporal trends in DEXA use
Tests ordered per 10,000 person-years
70
60
50
40
30
20
10
0
2000
2002
2004
2006
2008
2010
2012
2014
Years
2000-2002 APC: 40%
2002-20015 APC: 6%

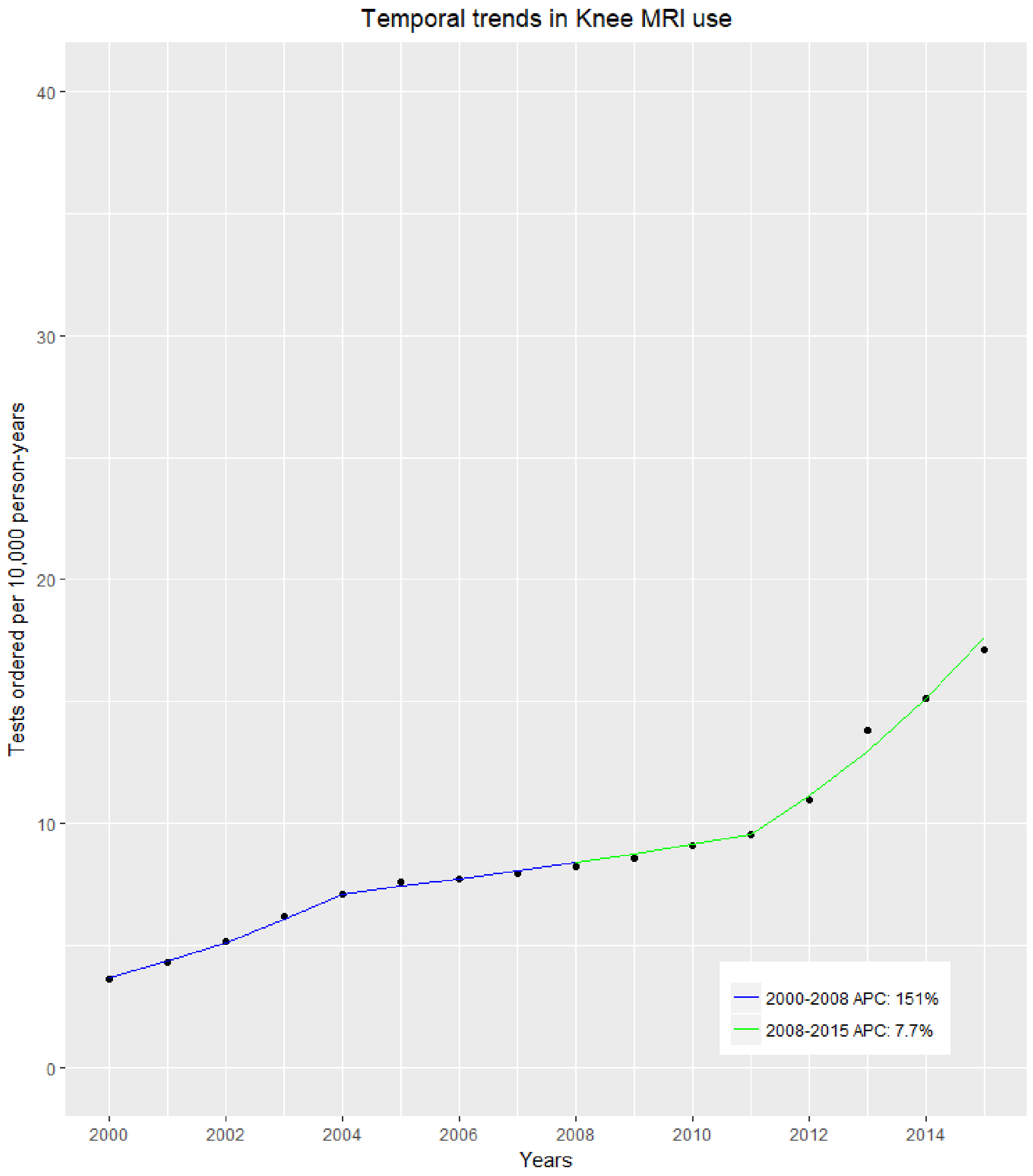
Temporal trends in Knee MRI use
Tests ordered per 10,000 person-years
40
30
20
10
0
2000
2002
2004
2006
2008
2010
2012
2014
Years
2000-2008 APC: 151%
2008-2015 APC: 7.7%

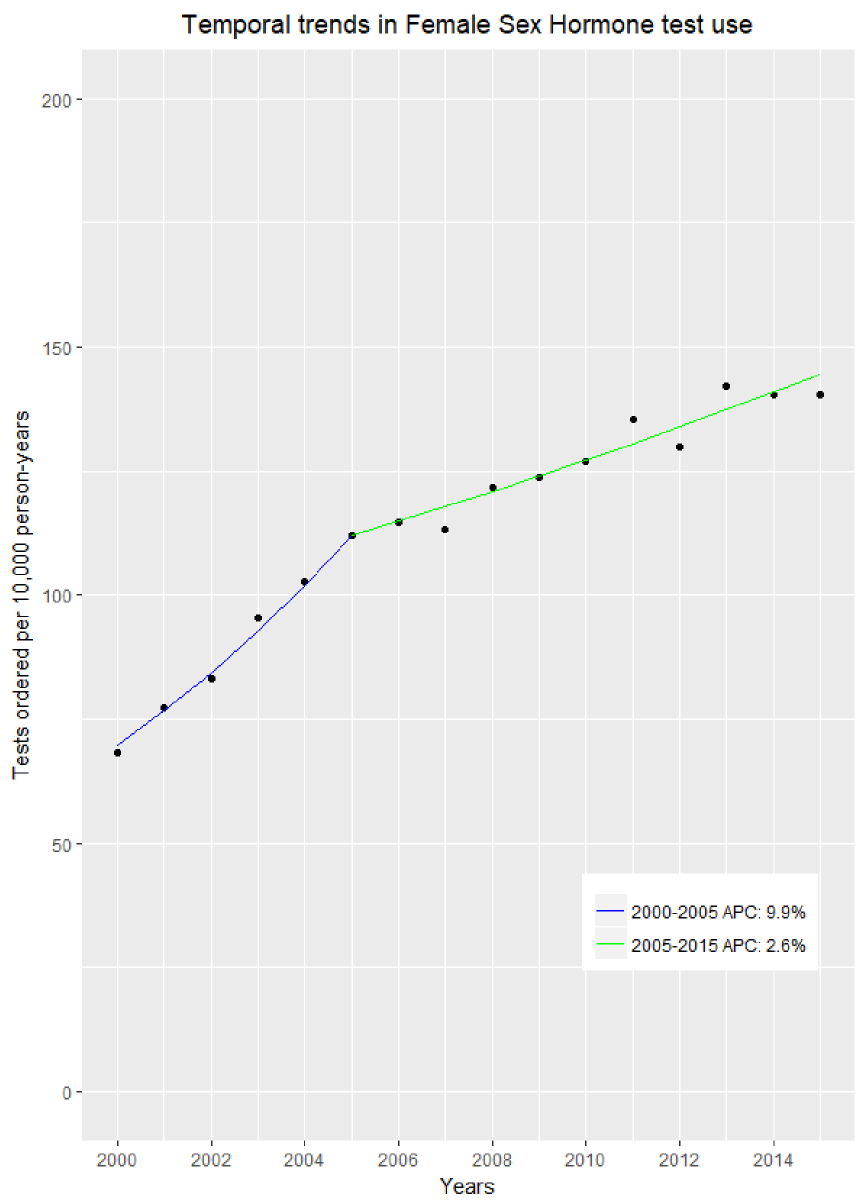
Temporal trends in Female Sex Hormone test use
Tests ordered per 10,000 person-years
200
150
100
50
0
2000
2002
2004
2006
2008
2010
2012
2014
Years
2000-2005 APC: 9.9%
2005-2015 APC: 2.6%

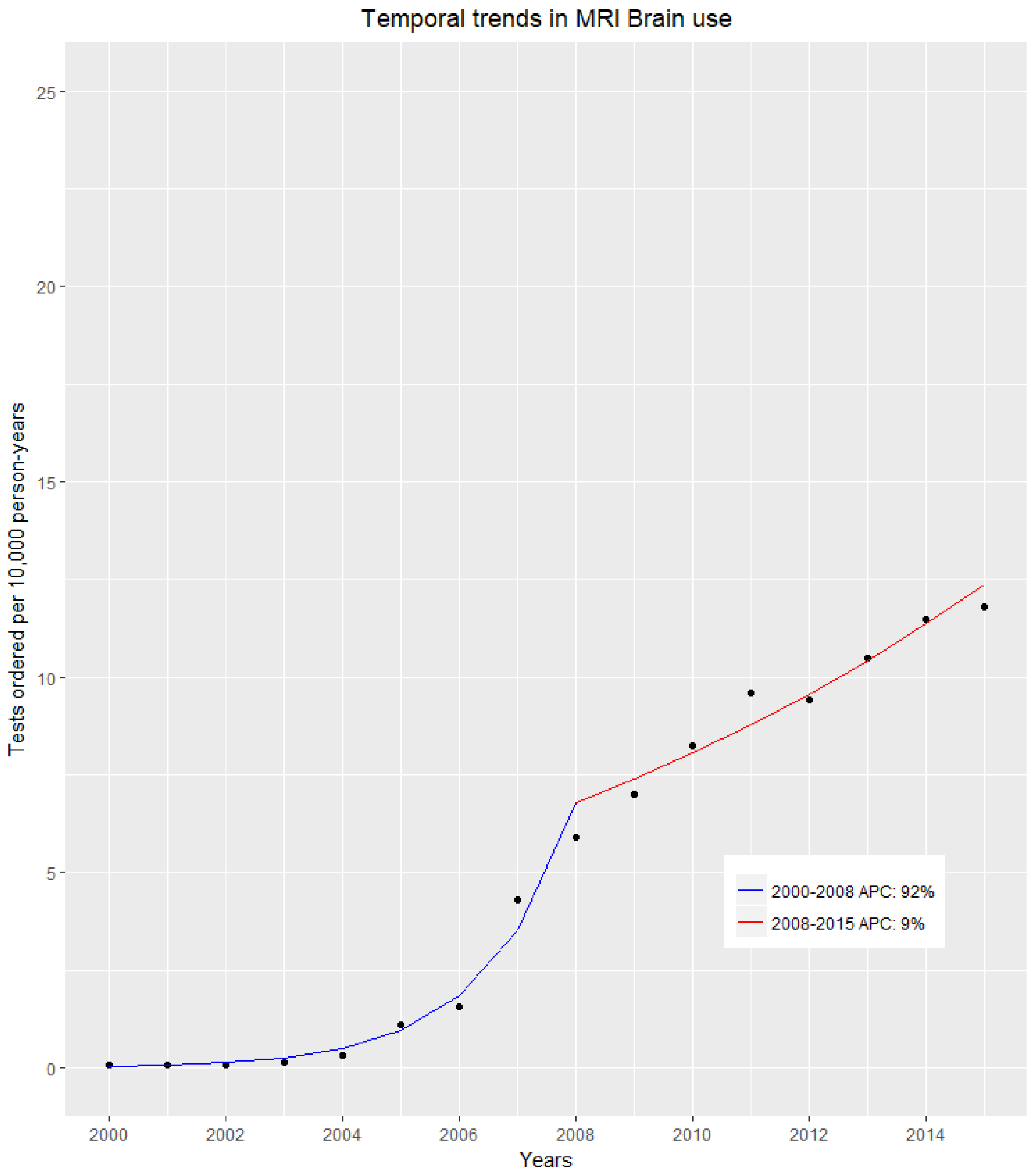

Temporal trends in MRI Brain use
Tests ordered per 10,000 person-years
Years
2000-2008 APC: 92%
2008-2015 APC: 9%

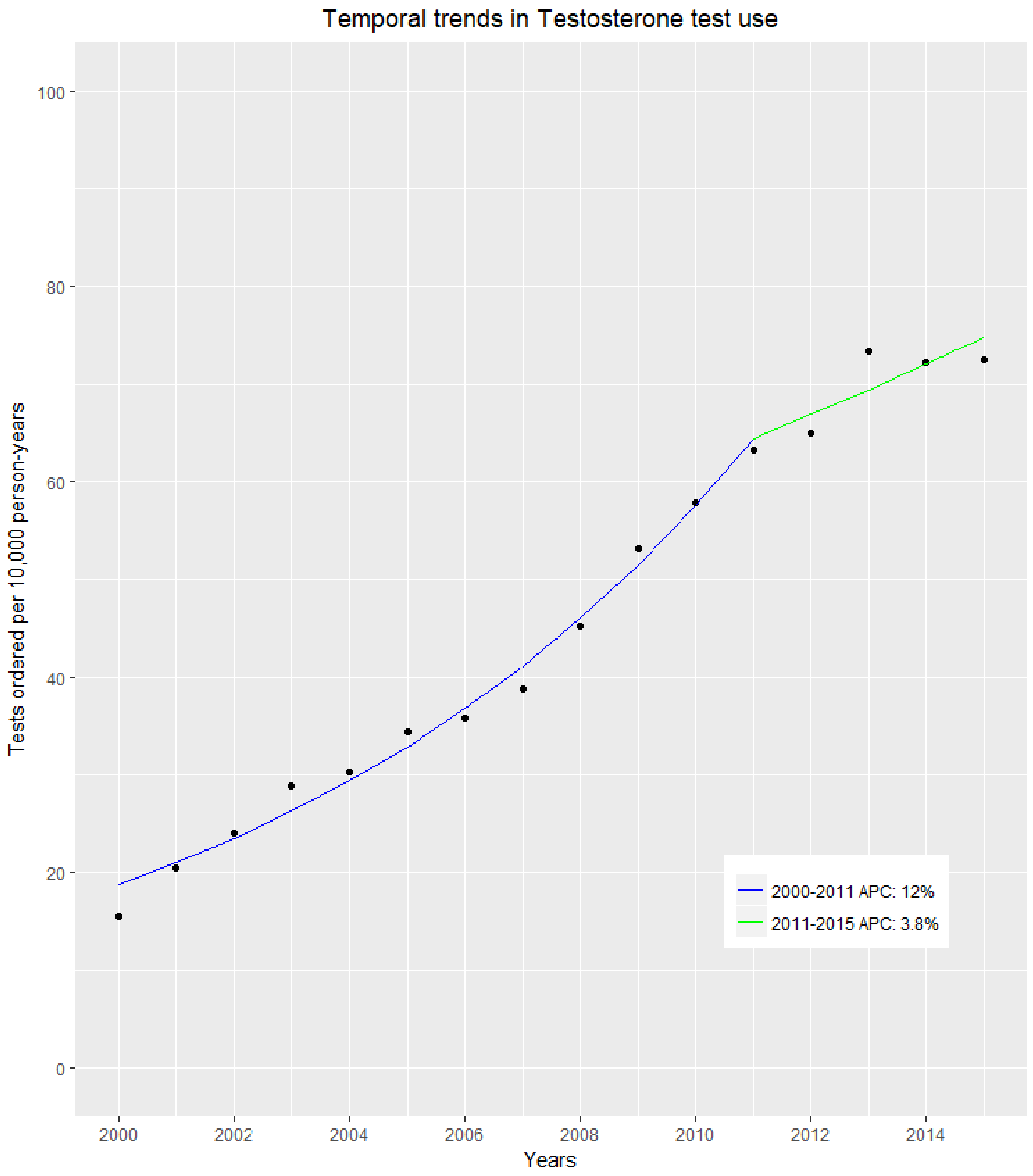

Temporal trends in Testosterone test use
Tests ordered per 10,000 person-years
100
80
60
40
20
0
2000
2002
2004
2006
2008
2010
2012
2014
Years
2000-2011 APC: 12%
2011-2015 APC: 3.8%

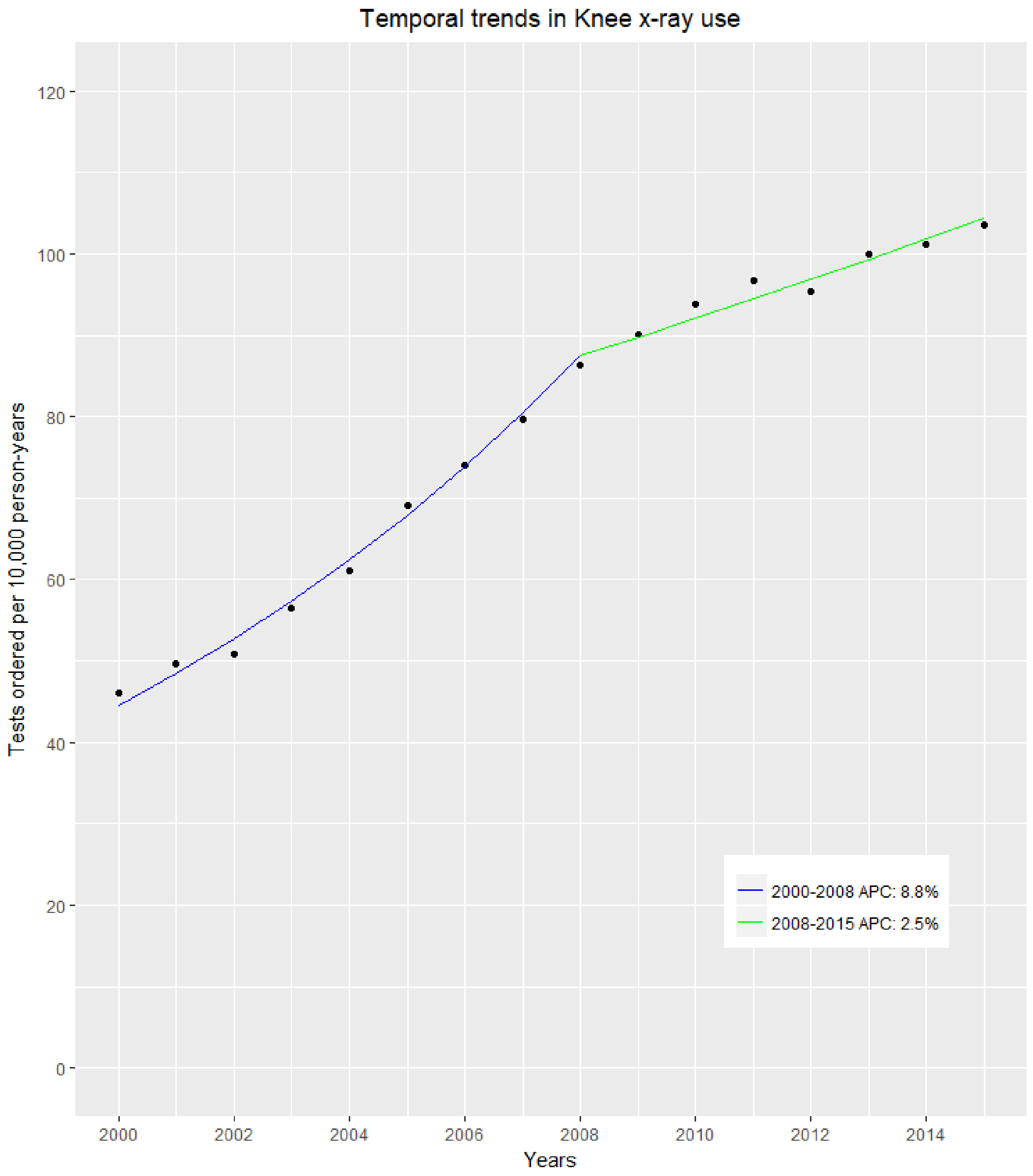
Temporal trends in Knee x-ray use
Tests ordered per 10,000 person-years
0
20
40
60
80
100
120
2000
2002
2004
2006
2008
2010
2012
2014
Years
2000-2008 APC: 8.8%
2008-2015 APC: 2.5%

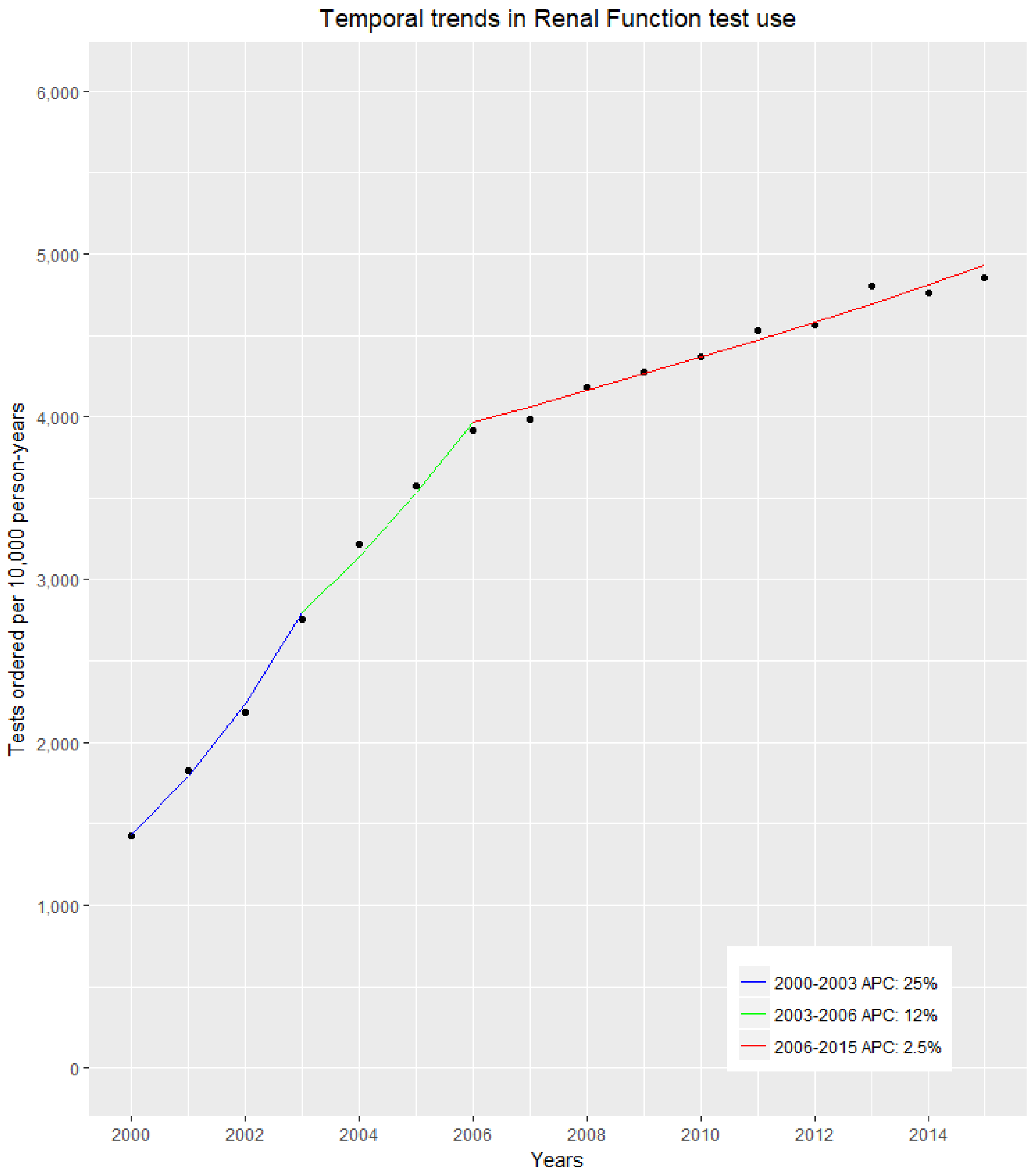
Temporal trends in Renal Function test use
Tests ordered per 10,000 person-years
6,000
5,000
4,000
3,000
2,000
1,000
0
2000
2002
2004
2006
2008
2010
2012
2014
Years
2000-2003 APC: 25%
2003-2006 APC: 12%
2006-2015 APC: 2.5%

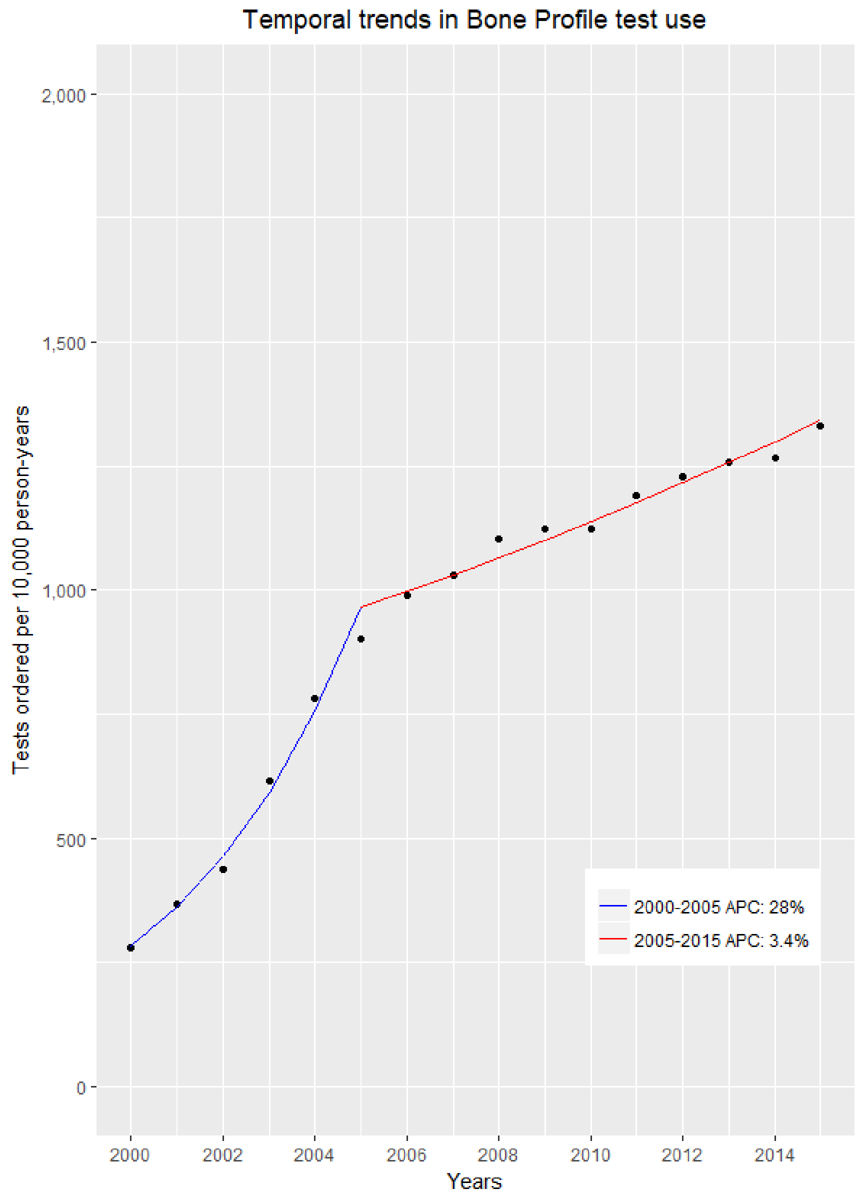

Temporal trends in Bone Profile test use
Tests ordered per 10,000 person-years
2,000
1,500
1,000
500
0
2000
2002
2004
2006
2008
2010
2012
2014
Years
2000-2005 APC: 28%
2005-2015 APC: 3.4%

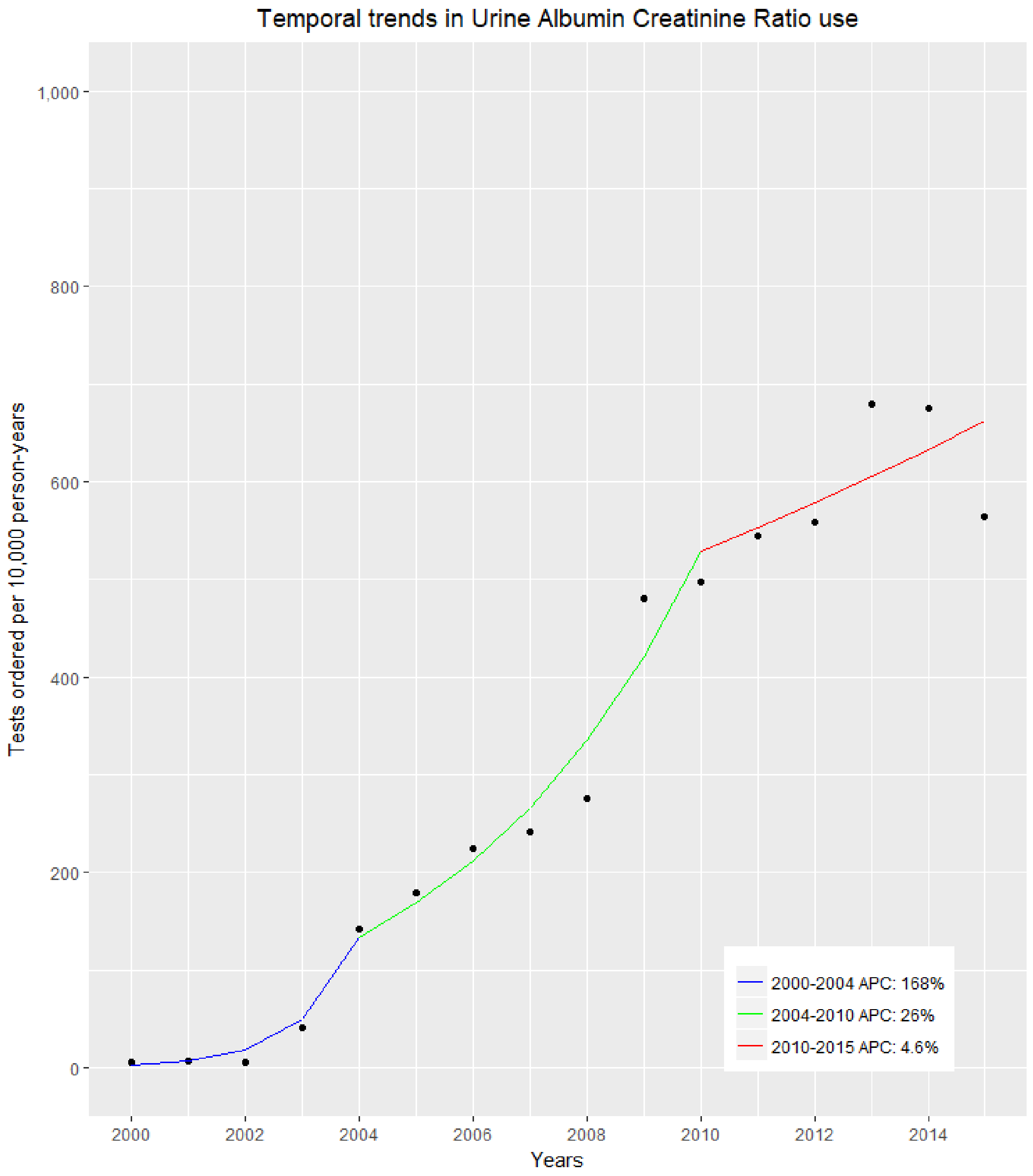
Temporal trends in Urine Albumin Creatinine Ratio use
Tests ordered per 10,000 person-years
1,000
800
600
400
200
0
2000
2002
2004
2006
2008
2010
2012
2014
Years
2000-2004 APC: 168%
2004-2010 APC: 26%
2010-2015 APC: 4.6%

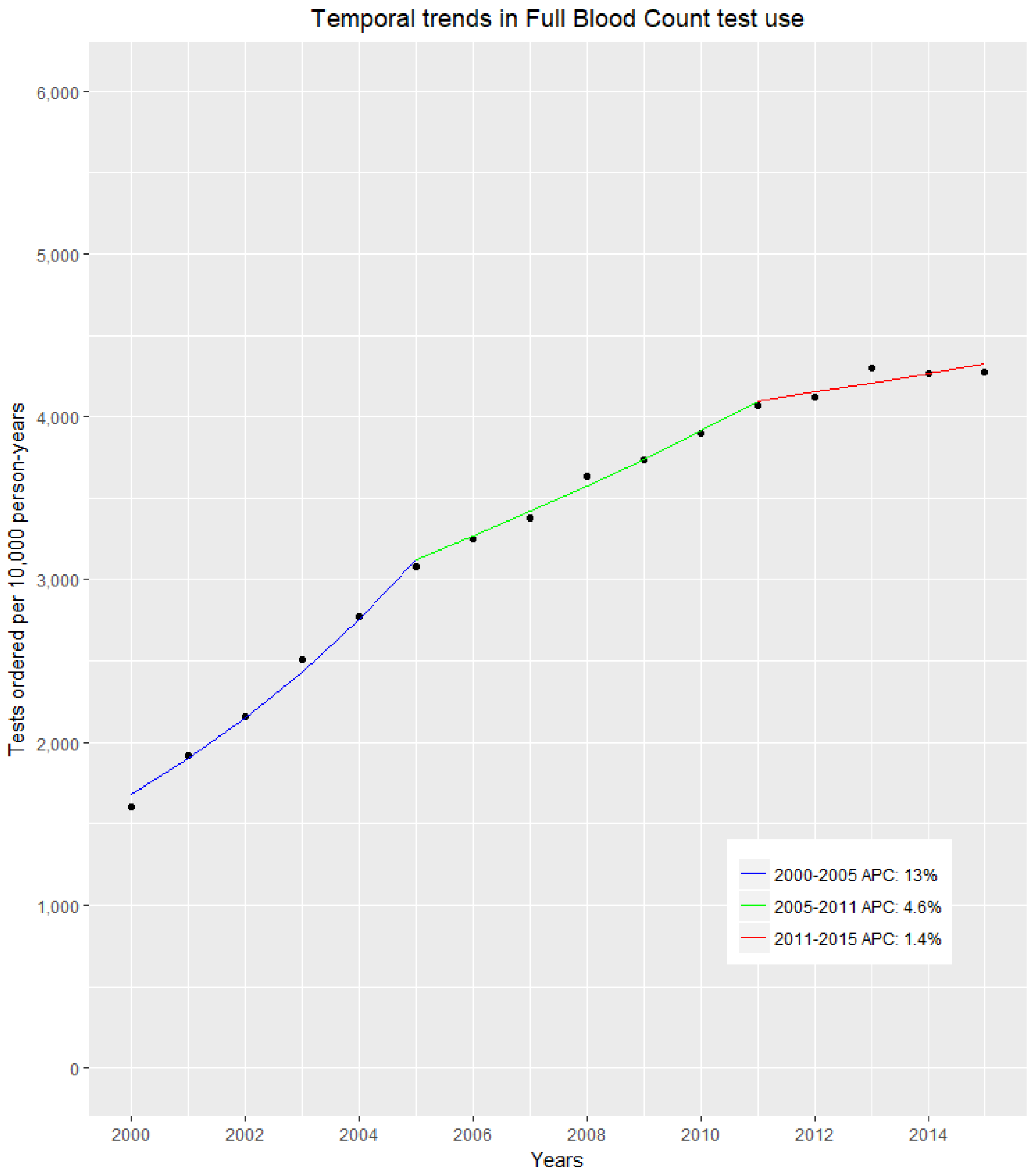

Temporal trends in Full Blood Count test use
Tests ordered per 10,000 person-years
6,000
5,000
4,000
3,000
2,000
1,000
0
2000
2002
2004
2006
2008
2010
2012
2014
Years
2000-2005 APC: 13%
2005-2011 APC: 4.6%
2011-2015 APC: 1.4%

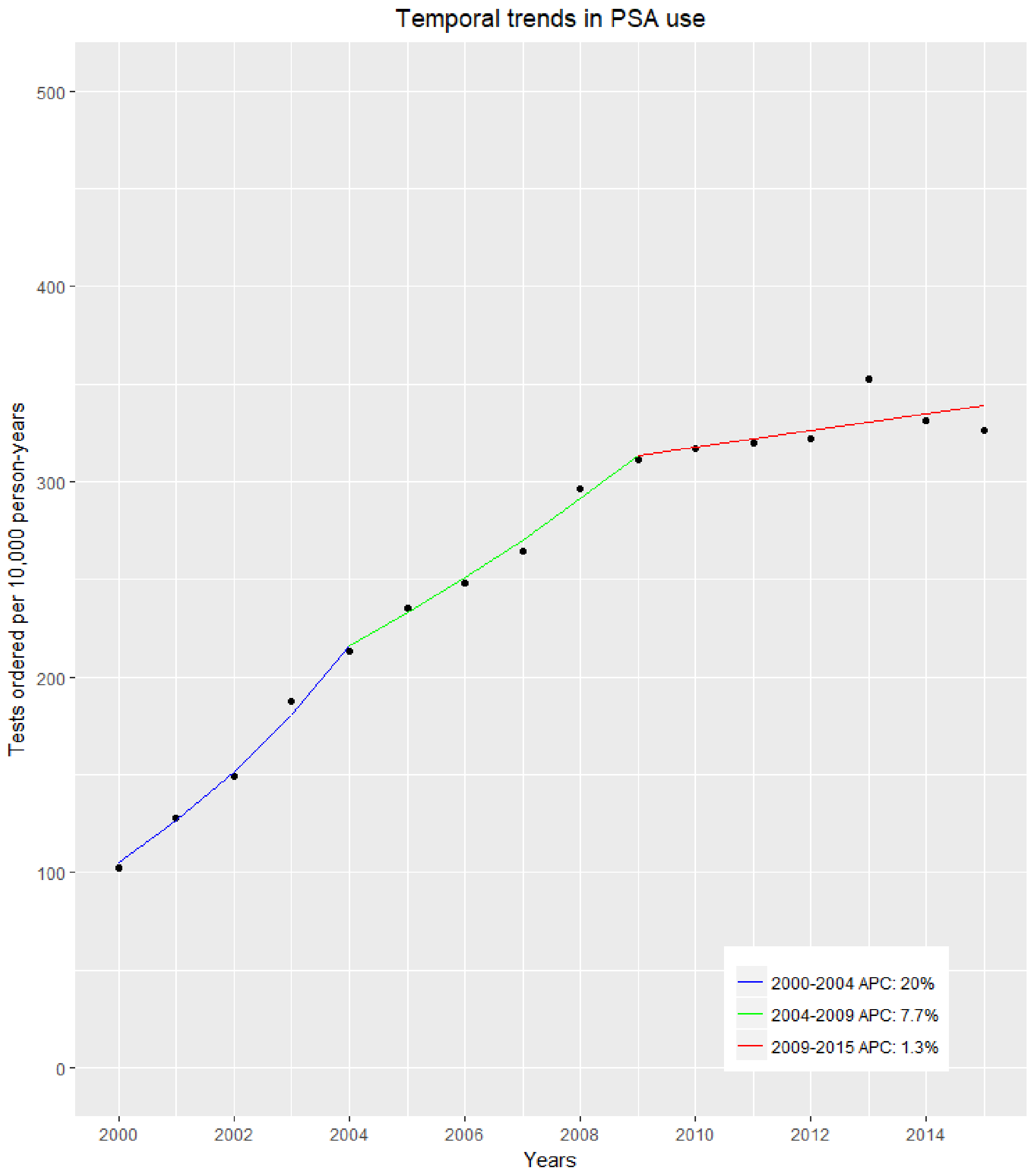

Temporal trends in PSA use
Tests ordered per 10,000 person-years
500
400
300
200
100
0
2000
2002
2004
2006
2008
2010
2012
2014
Years
2000-2004 APC: 20%
2004-2009 APC: 7.7%
2009-2015 APC: 1.3%

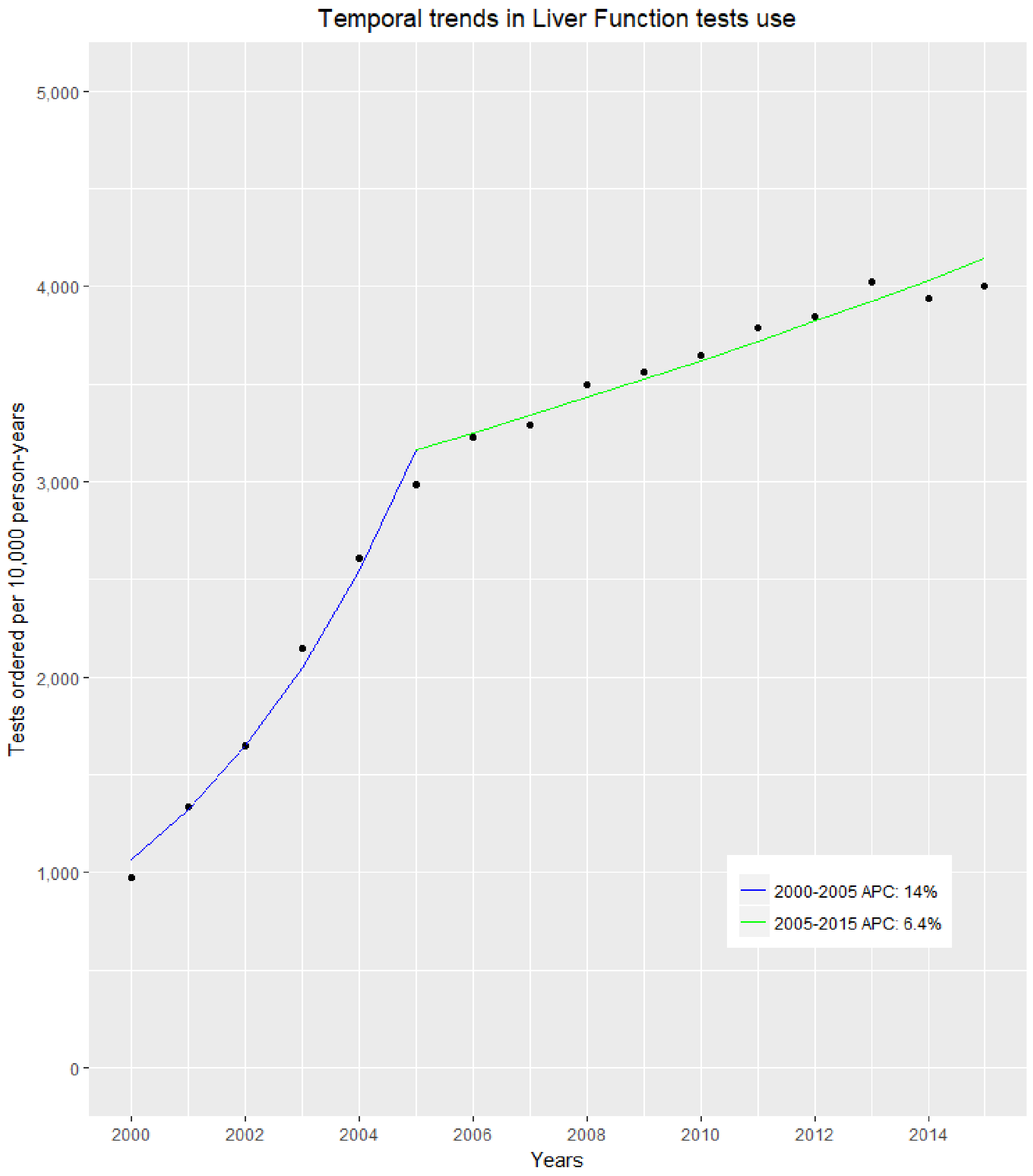
Temporal trends in Liver Function tests use
Tests ordered per 10,000 person-years
5,000
4,000
3,000
2,000
1,000
0
2000
2002
2004
2006
2008
2010
2012
2014
Years
2000-2005 APC: 14%
2005-2015 APC: 6.4%

Pattern: Plateau

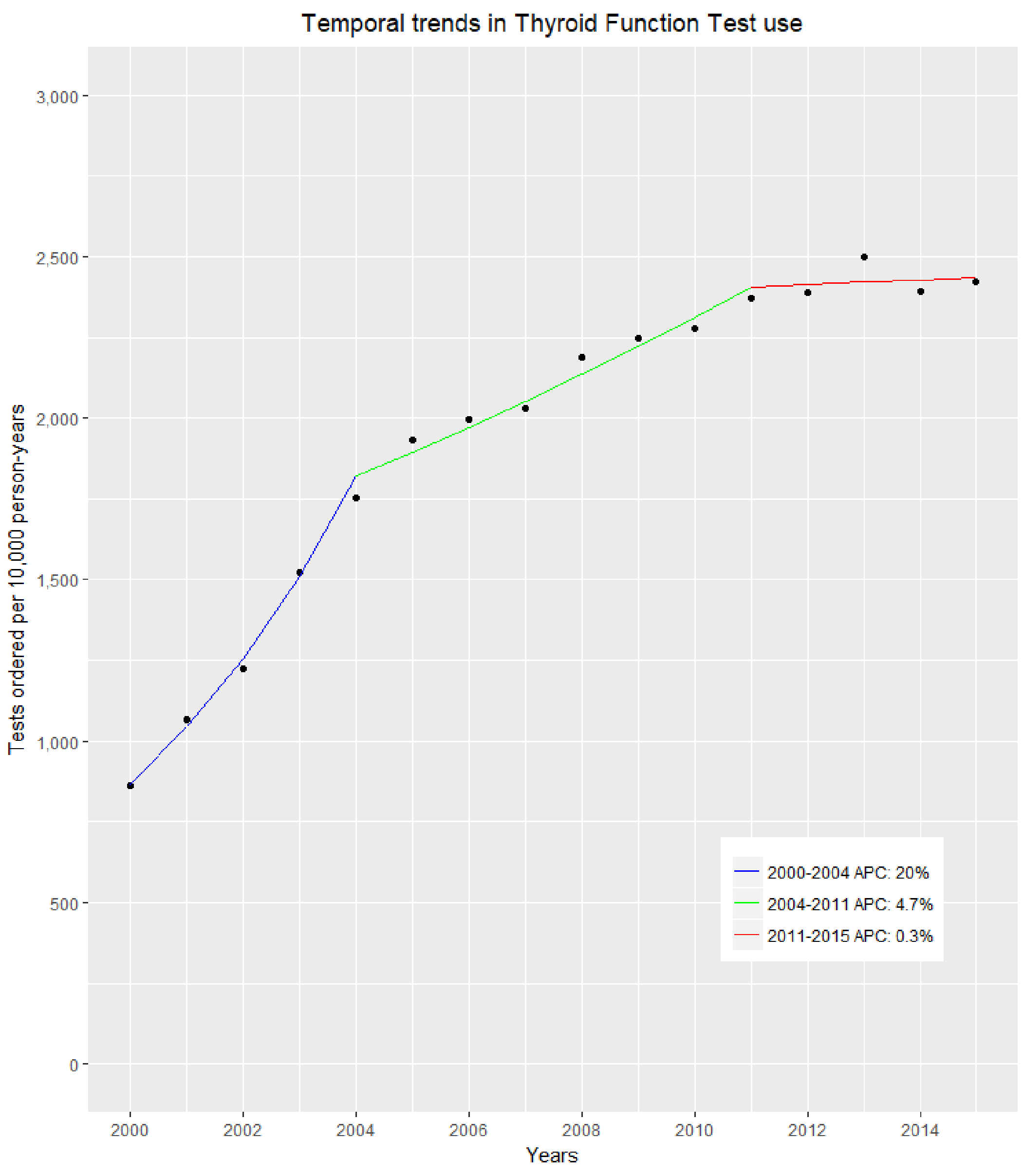

Temporal trends in Thyroid Function Test use
Tests ordered per 10,000 person-years
Years
0
500
1,000
1,500
2,000
2,500
3,000
2000
2002
2004
2006
2008
2010
2012
2014
2000-2004 APC: 20%
2004-2011 APC: 4.7%
2011-2015 APC: 0.3%

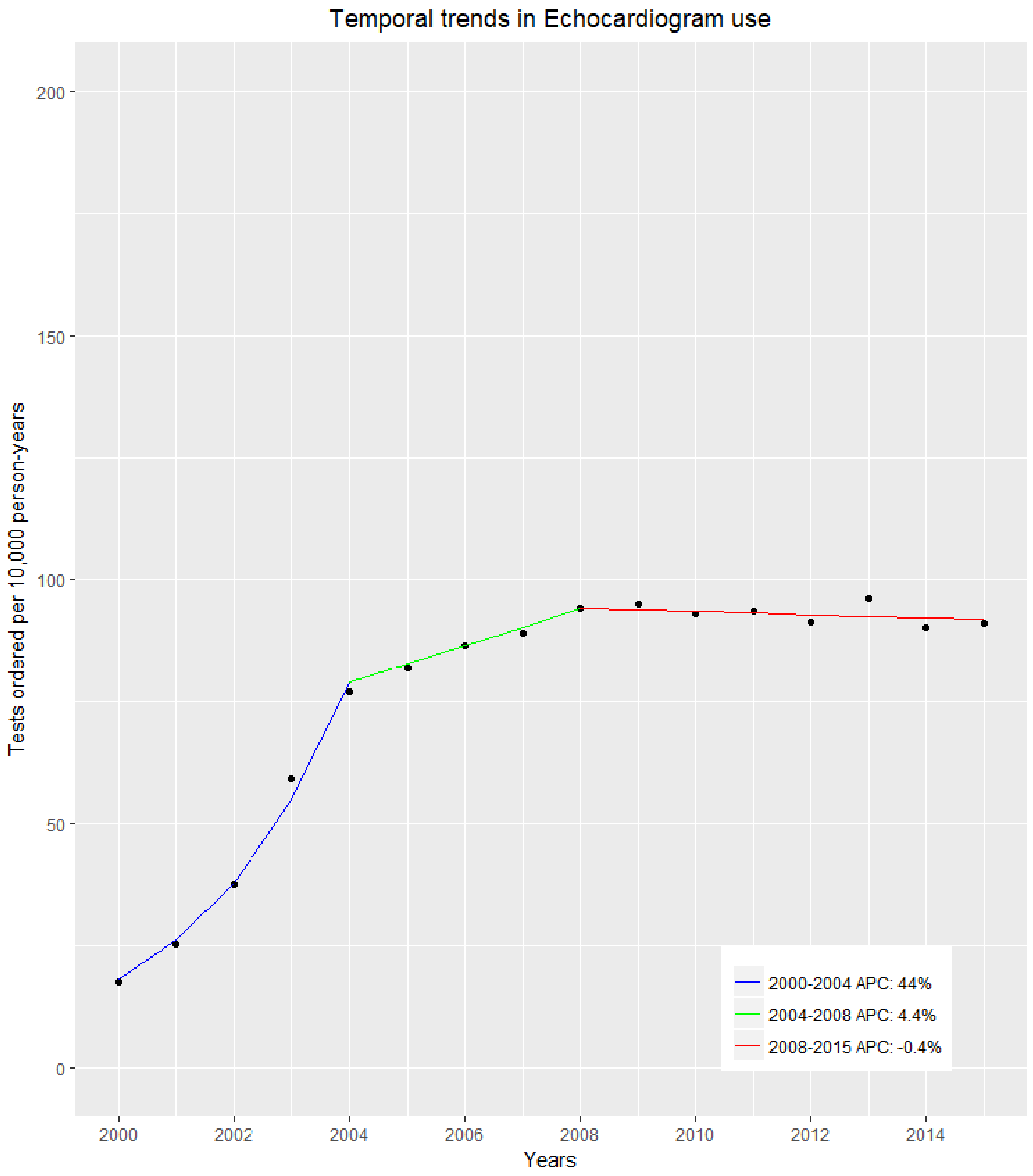

Temporal trends in Echocardiogram use
Tests ordered per 10,000 person-years
200
150
100
50
0
2000
2002
2004
2006
2008
2010
2012
2014
Years
2000-2004 APC: 44%
2004-2008 APC: 4.4%
2008-2015 APC: -0.4%

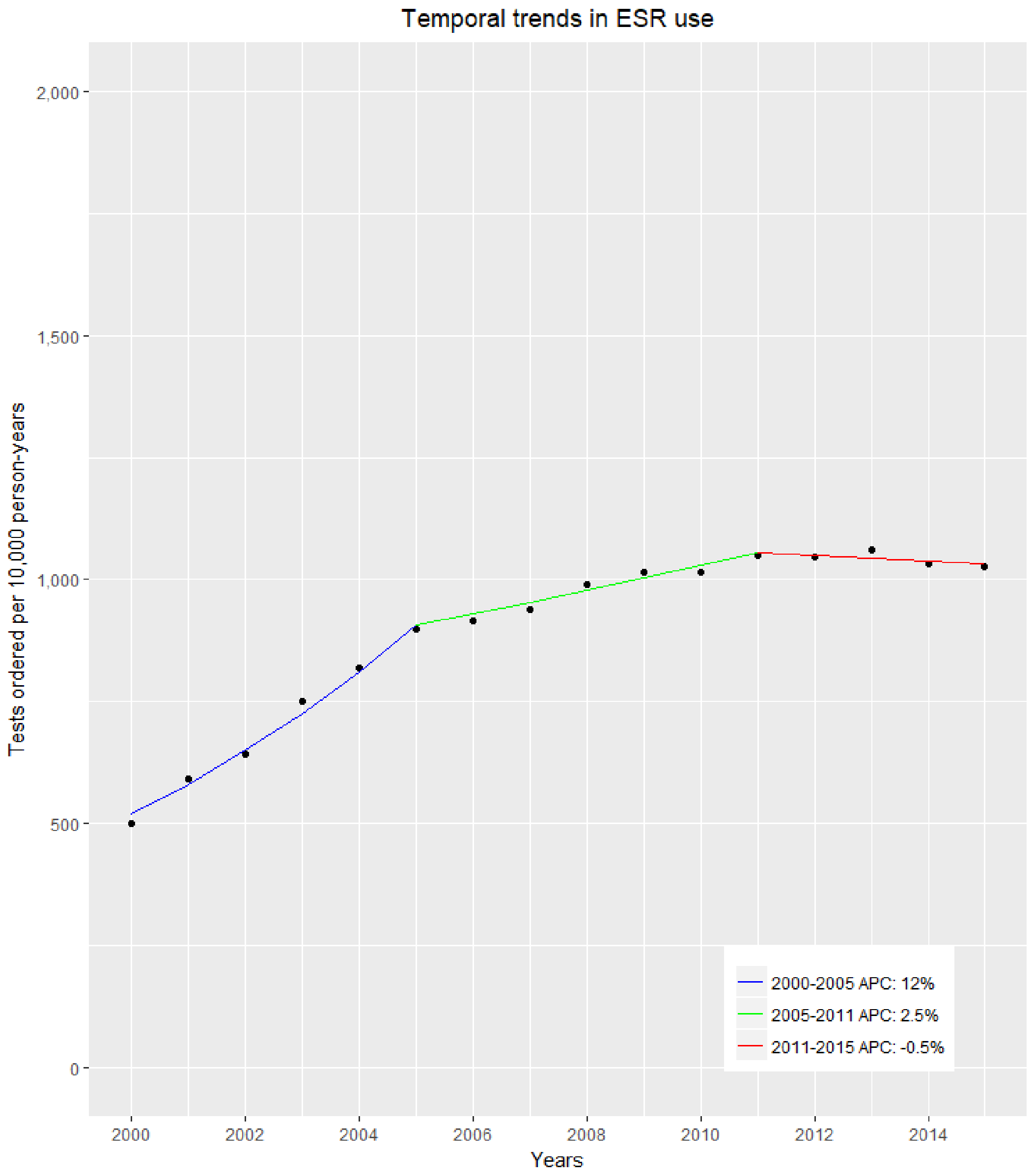

Temporal trends in ESR use
Tests ordered per 10,000 person-years
2,000
1,500
1,000
500
0
2000
2002
2004
2006
2008
2010
2012
2014
Years
2000-2005 APC: 12%
2005-2011 APC: 2.5%
2011-2015 APC: -0.5%

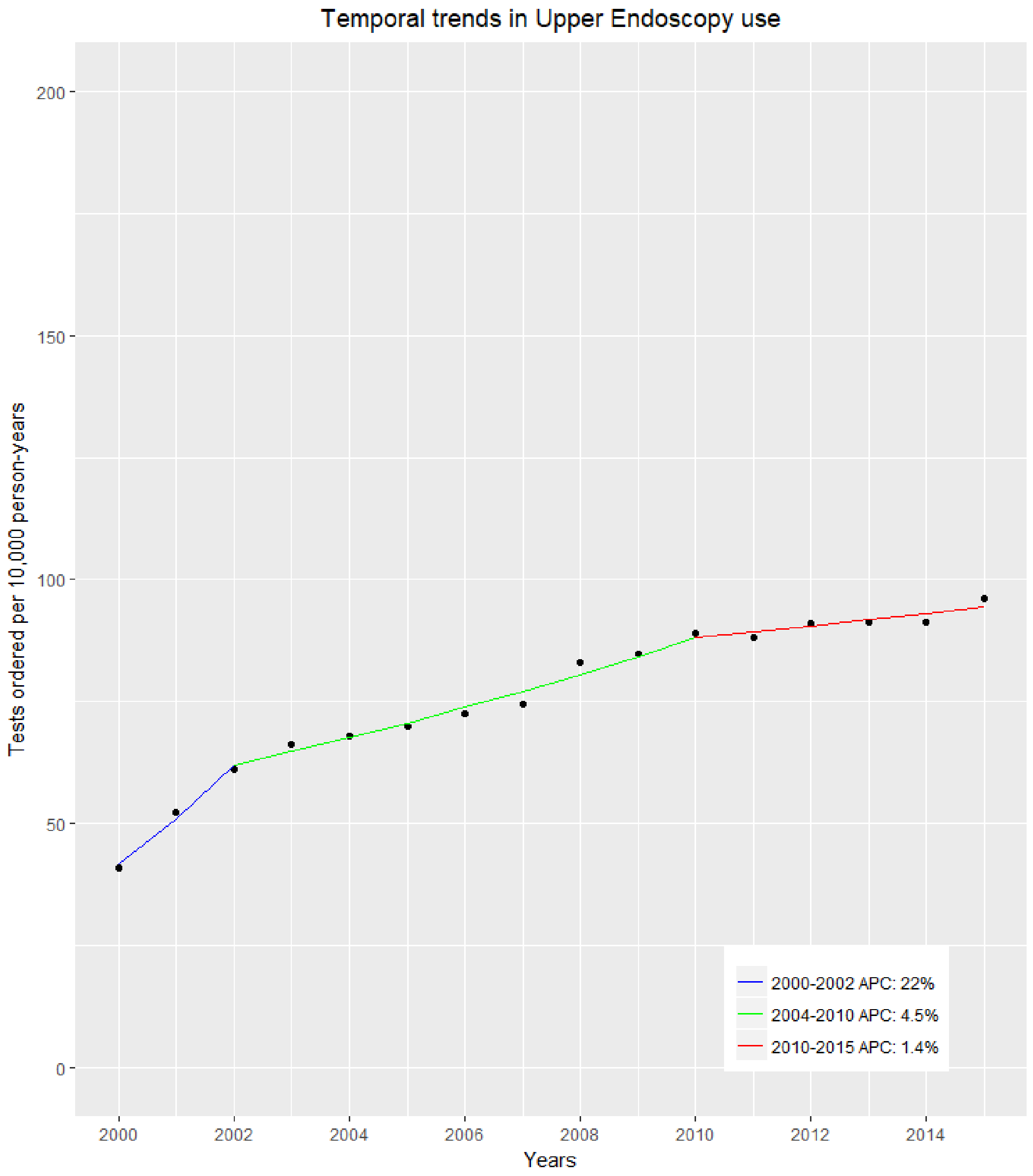
Temporal trends in Upper Endoscopy use
Tests ordered per 10,000 person-years
0
50
100
150
200
2000
2002
2004
2006
2008
2010
2012
2014
Years
2000-2002 APC: 22%
2004-2010 APC: 4.5%
2010-2015 APC: 1.4%

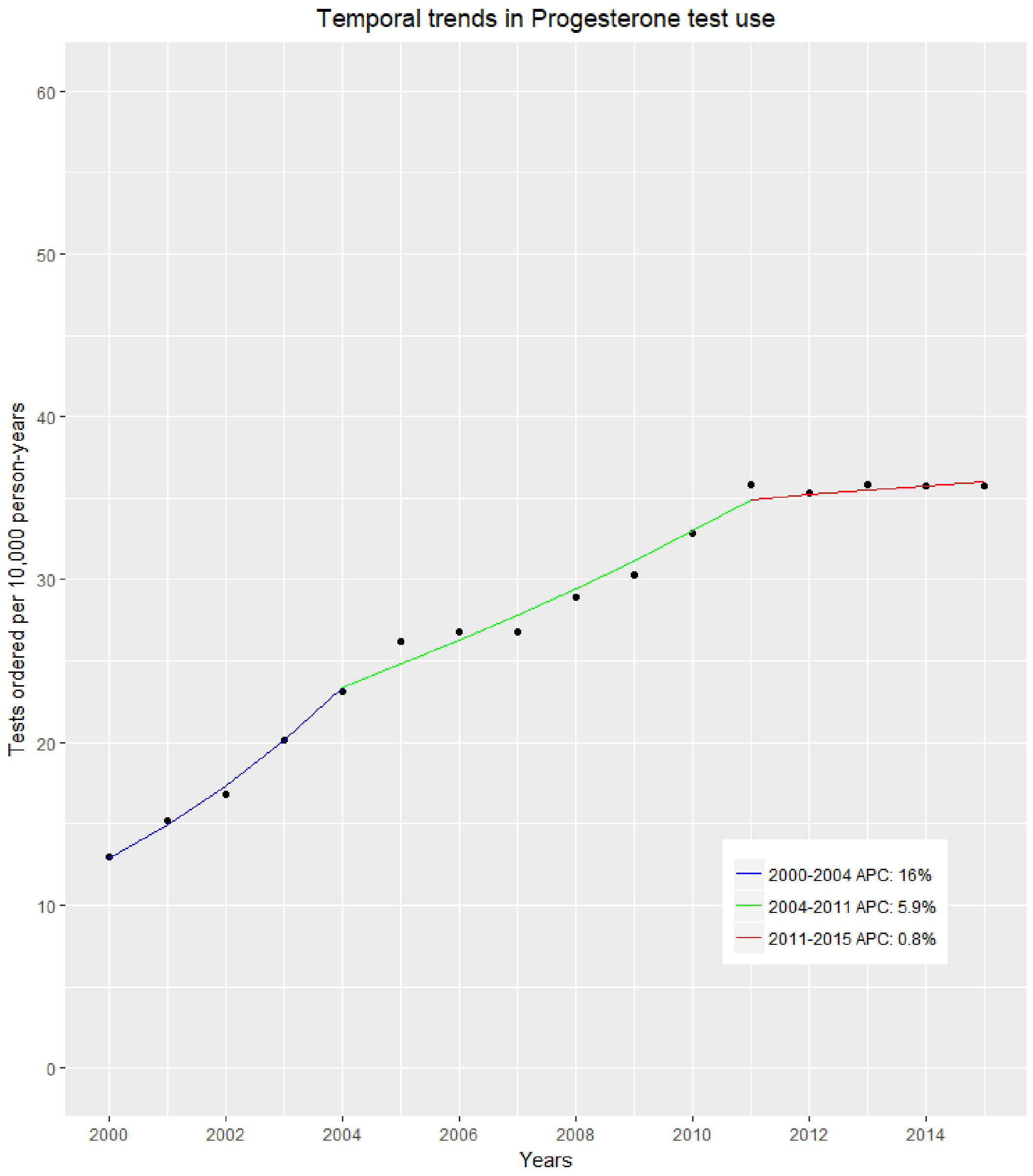

Temporal trends in Progesterone test use
Tests ordered per 10,000 person-years
0
10
20
30
40
50
60
2000
2002
2004
2006
2008
2010
2012
2014
Years
2000-2004 APC: 16%
2004-2011 APC: 5.9%
2011-2015 APC: 0.8%

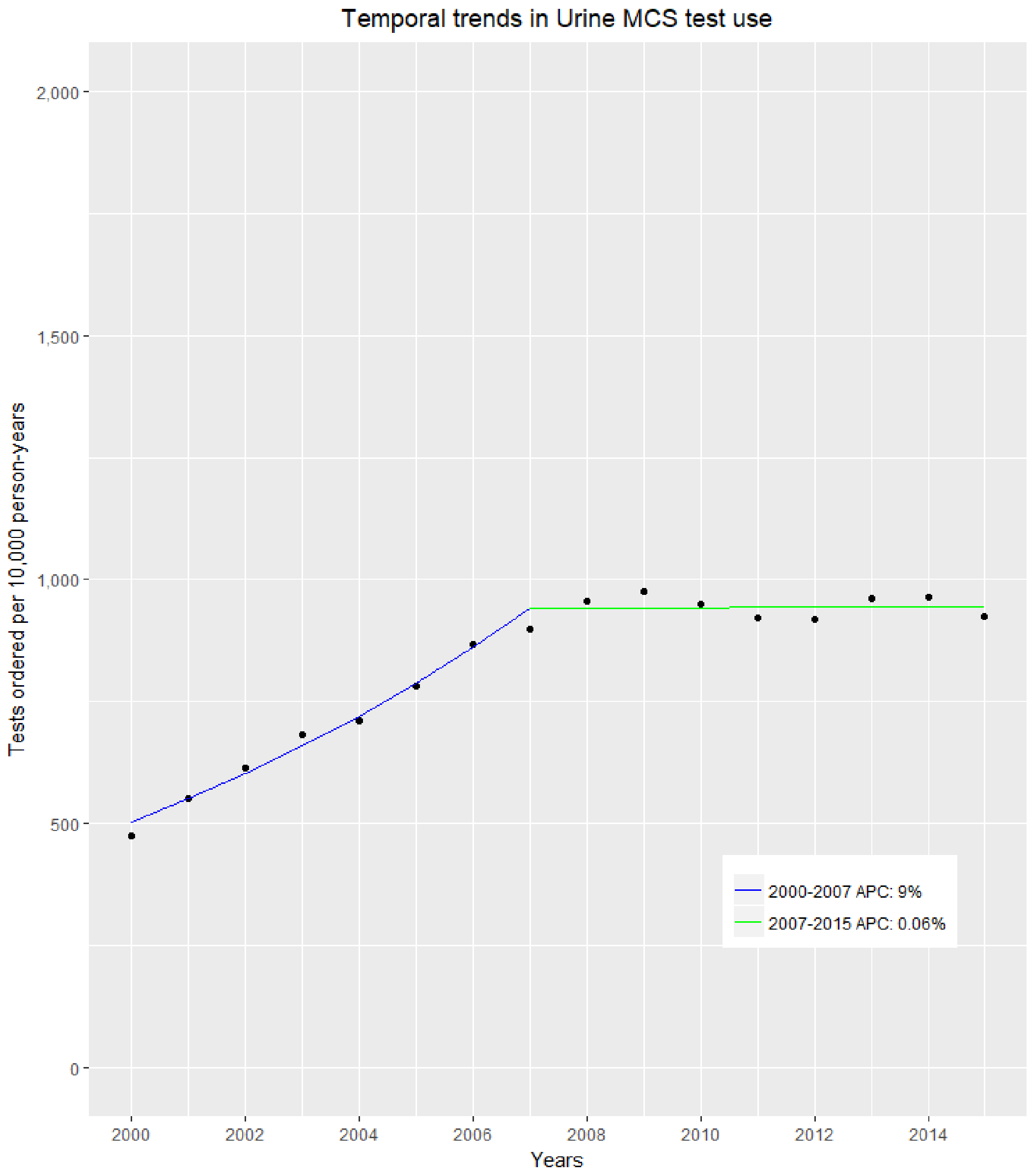

Temporal trends in Urine MCS test use
Tests ordered per 10,000 person-years
2,000
1,500
1,000
500
0
2000
2002
2004
2006
2008
2010
2012
2014
Years
2000-2007 APC: 9%
2007-2015 APC: 0.06%

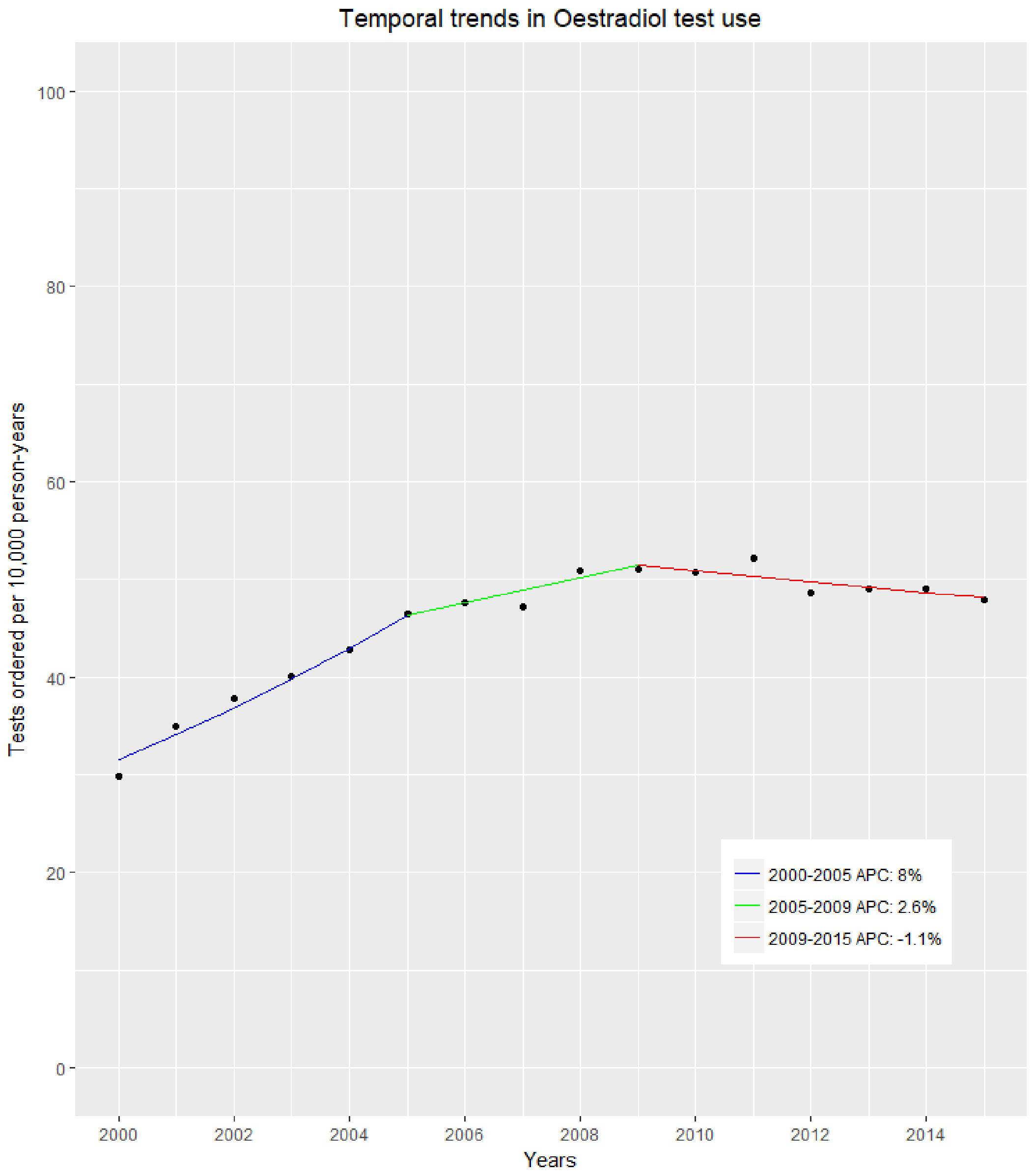

Temporal trends in Oestradiol test use
Tests ordered per 10,000 person-years
100
80
60
40
20
0
2000
2002
2004
2006
2008
2010
2012
2014
Years
2000-2005 APC: 8%
2005-2009 APC: 2.6%
2009-2015 APC: -1.1%

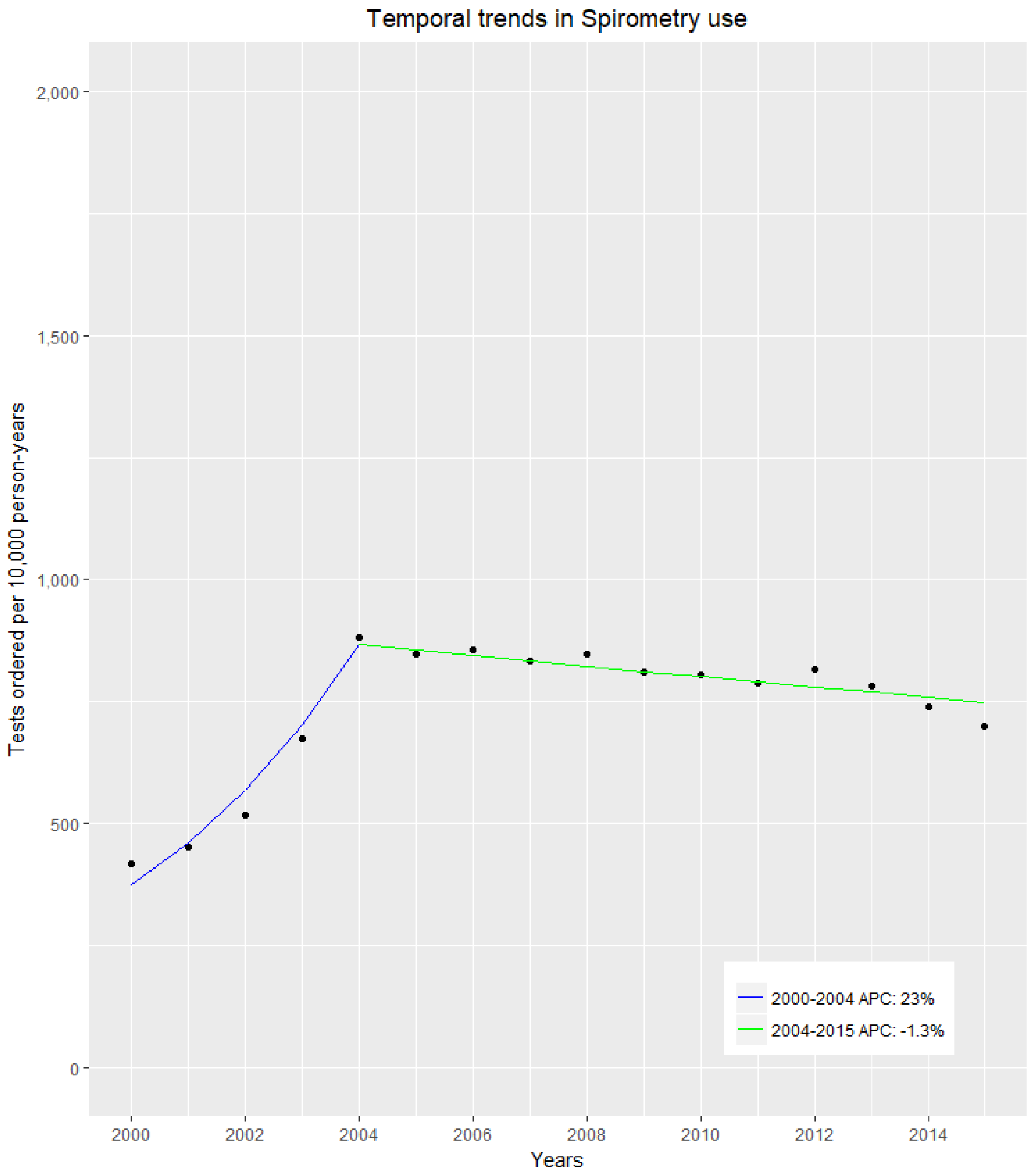
Temporal trends in Spirometry use
Tests ordered per 10,000 person-years
2,000
1,500
1,000
500
0
2000
2002
2004
2006
2008
2010
2012
2014
Years
2000-2004 APC: 23%
2004-2015 APC: -1.3%

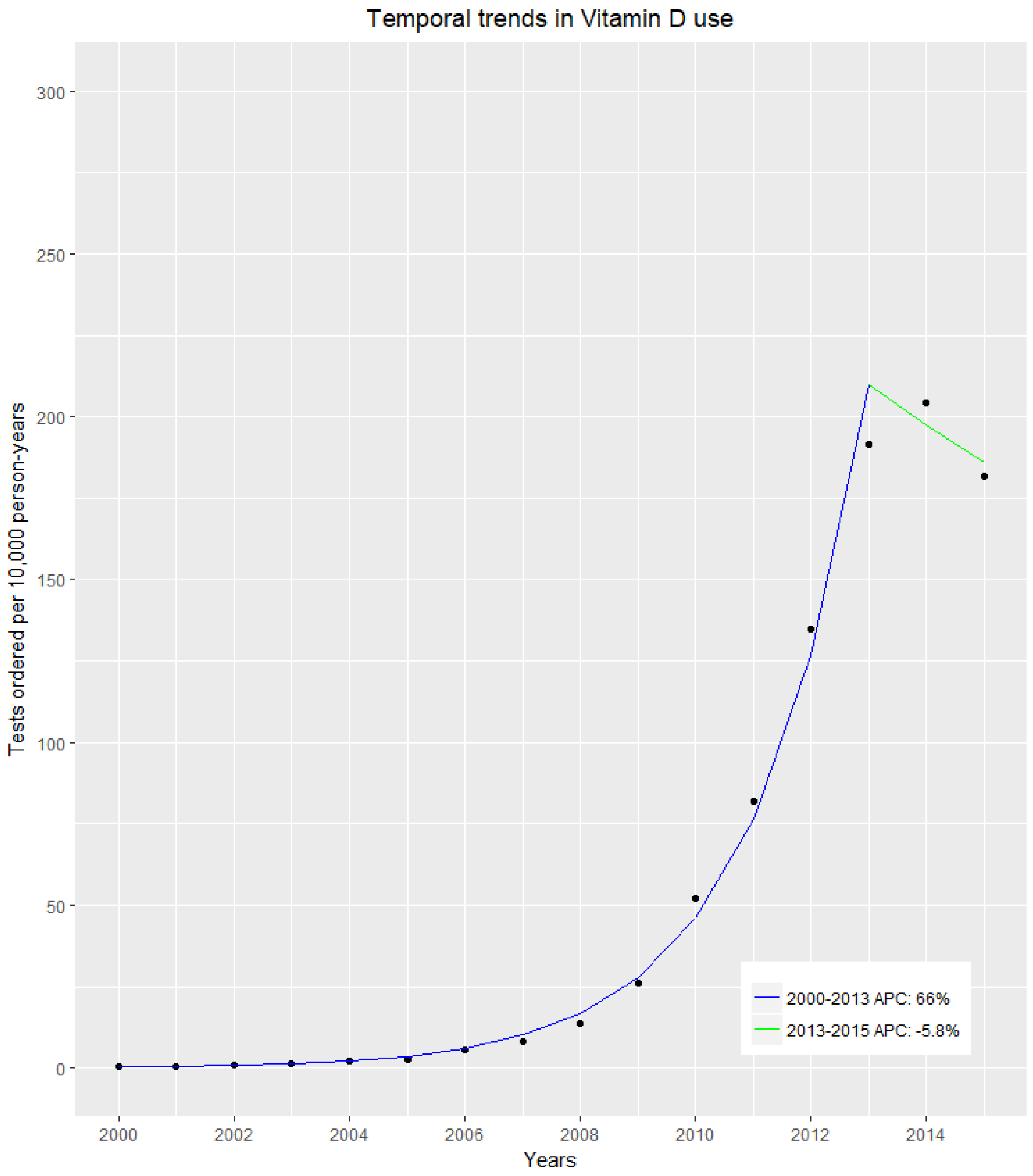
Temporal trends in Vitamin D use
Tests ordered per 10,000 person-years
300
250
200
150
100
50
0
2000
2002
2004
2006
2008
2010
2012
2014
Years
2000-2013 APC: 66%
2013-2015 APC: -5.8%

Pattern: Inverted U distribution

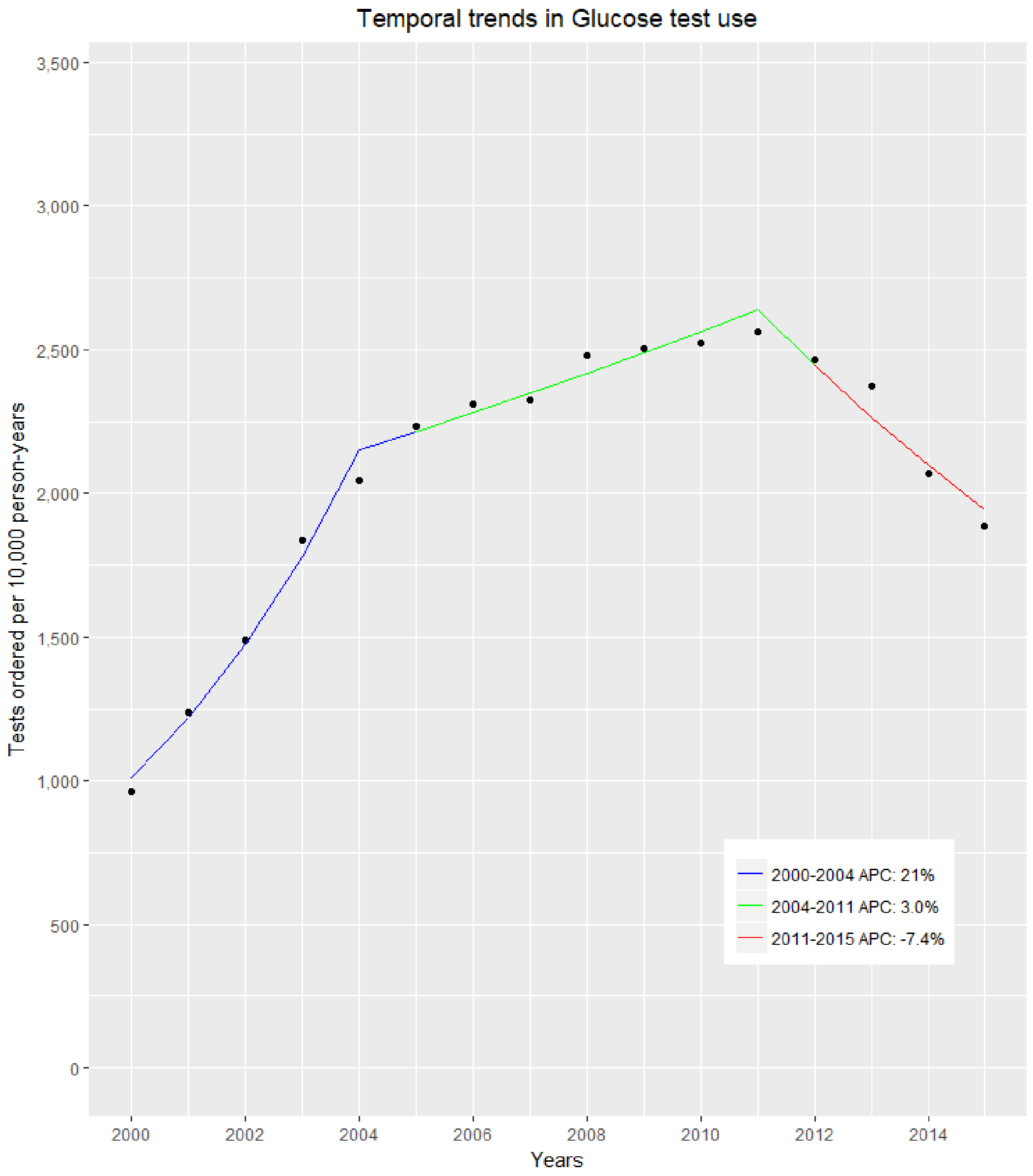

Temporal trends in Glucose test use
Tests ordered per 10,000 person-years
3,500
3,000
2,500
2,000
1,500
1,000
500
0
2000
2002
2004
2006
2008
2010
2012
2014
Years
2000-2004 APC: 21%
2004-2011 APC: 3.0%
2011-2015 APC: -7.4%

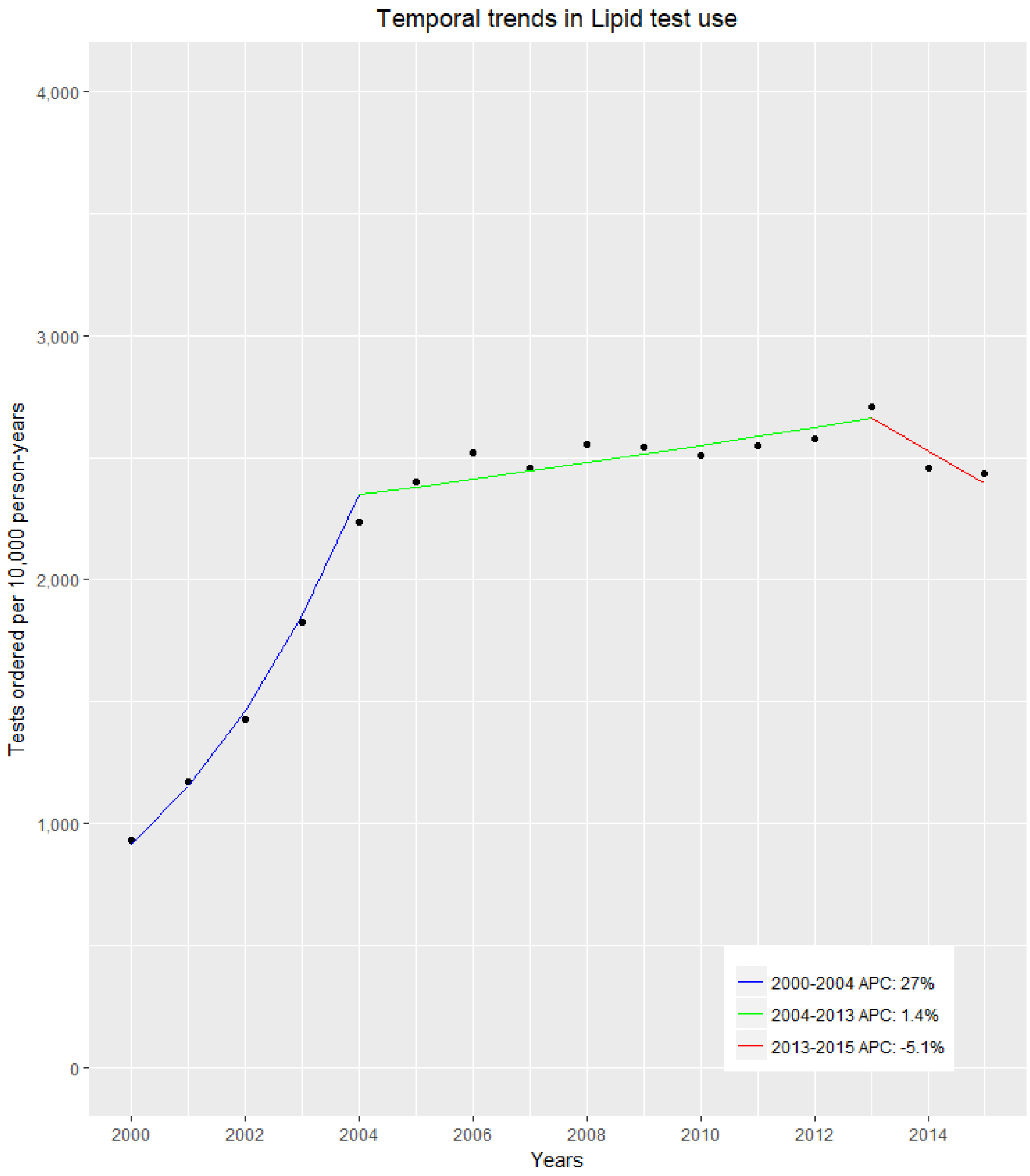

Temporal trends in Lipid test use
4,000
3,000
2,000
1,000
0
Tests ordered per 10,000 person-years
2000
2002
2004
2006
2008
2010
2012
2014
Years
2000-2004 APC: 27%
2004-2013 APC: 1.4%
2013-2015 APC: -5.1%

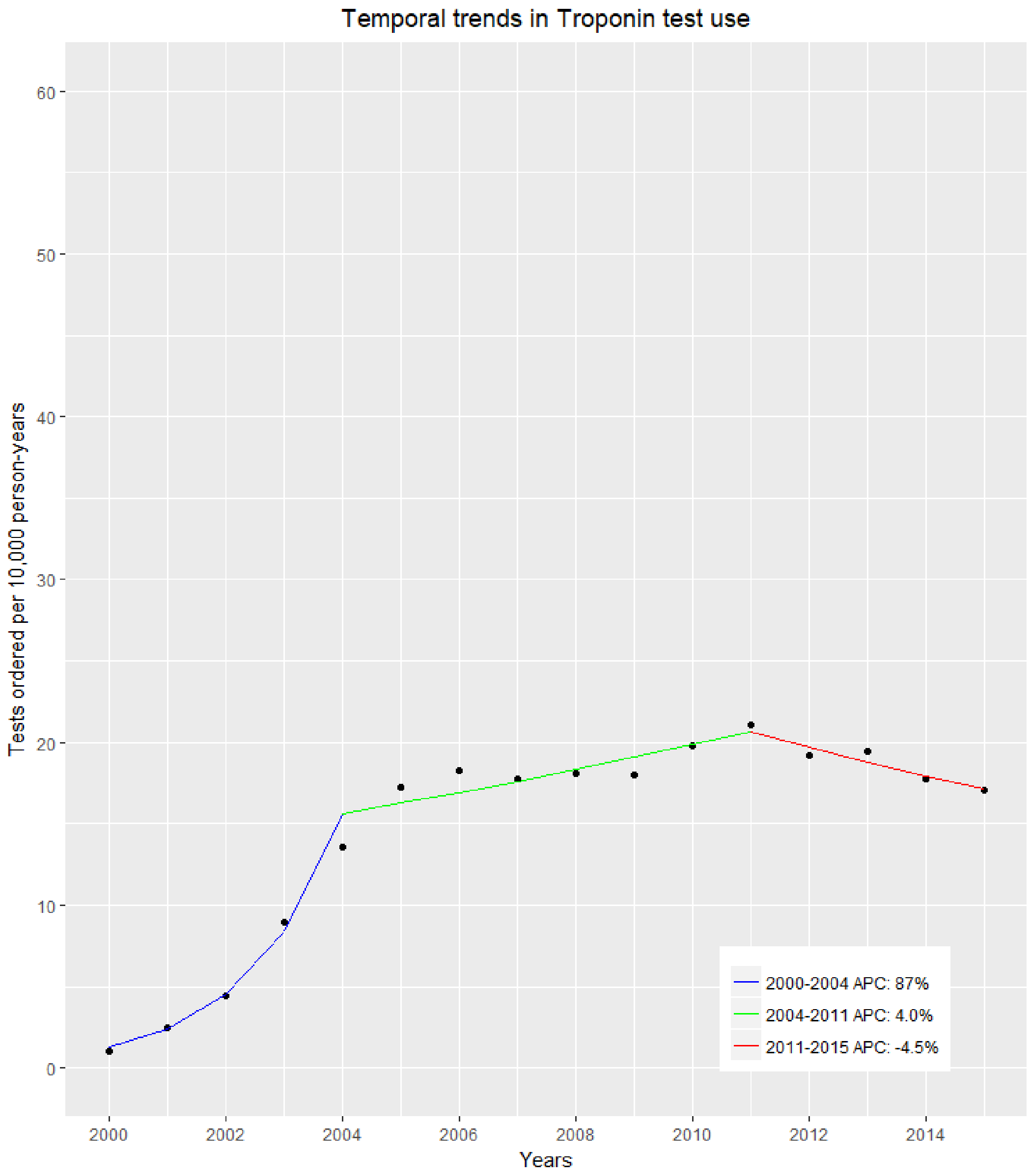

Temporal trends in Troponin test use
Tests ordered per 10,000 person-years
60
50
40
30
20
10
0
2000
2002
2004
2006
2008
2010
2012
2014
Years
2000-2004 APC: 87%
2004-2011 APC: 4.0%
2011-2015 APC: -4.5%

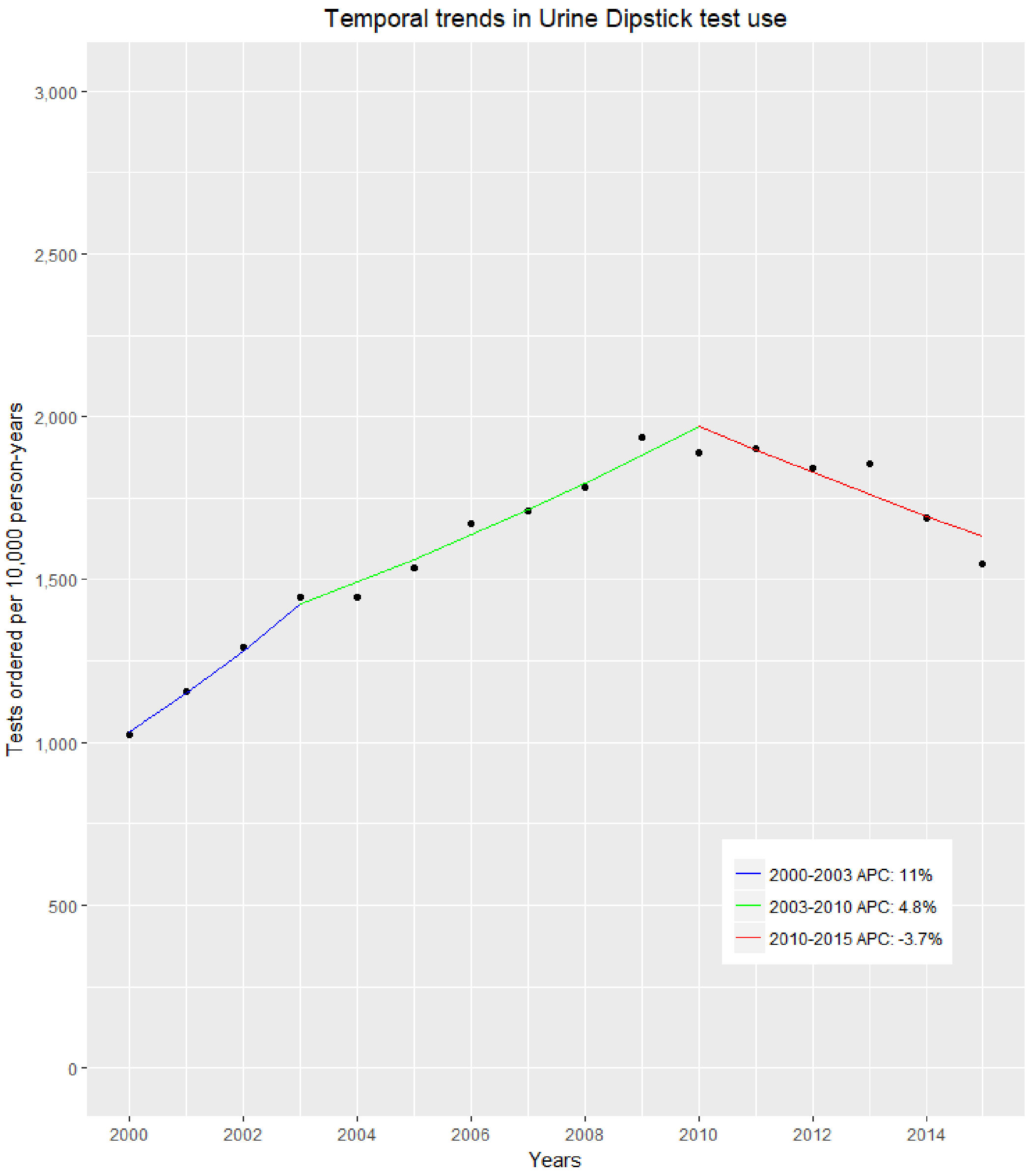

Temporal trends in Urine Dipstick test use
Tests ordered per 10,000 person-years
3,000
2,500
2,000
1,500
1,000
500
0
2000
2002
2004
2006
2008
2010
2012
2014
Years
2000-2003 APC: 11%
2003-2010 APC: 4.8%
2010-2015 APC: -3.7%

Temporal trends in Urine Microalbumin test use

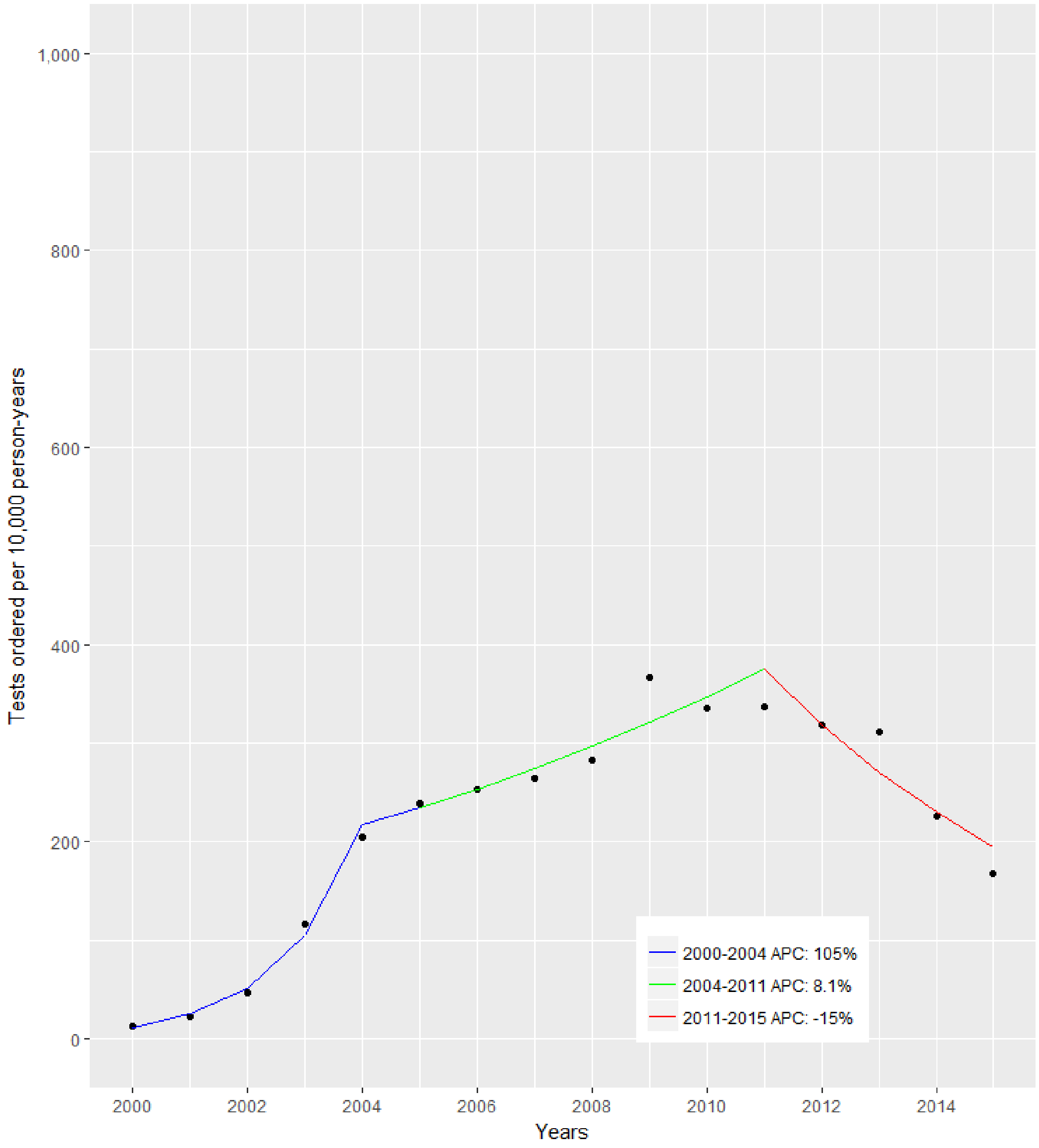

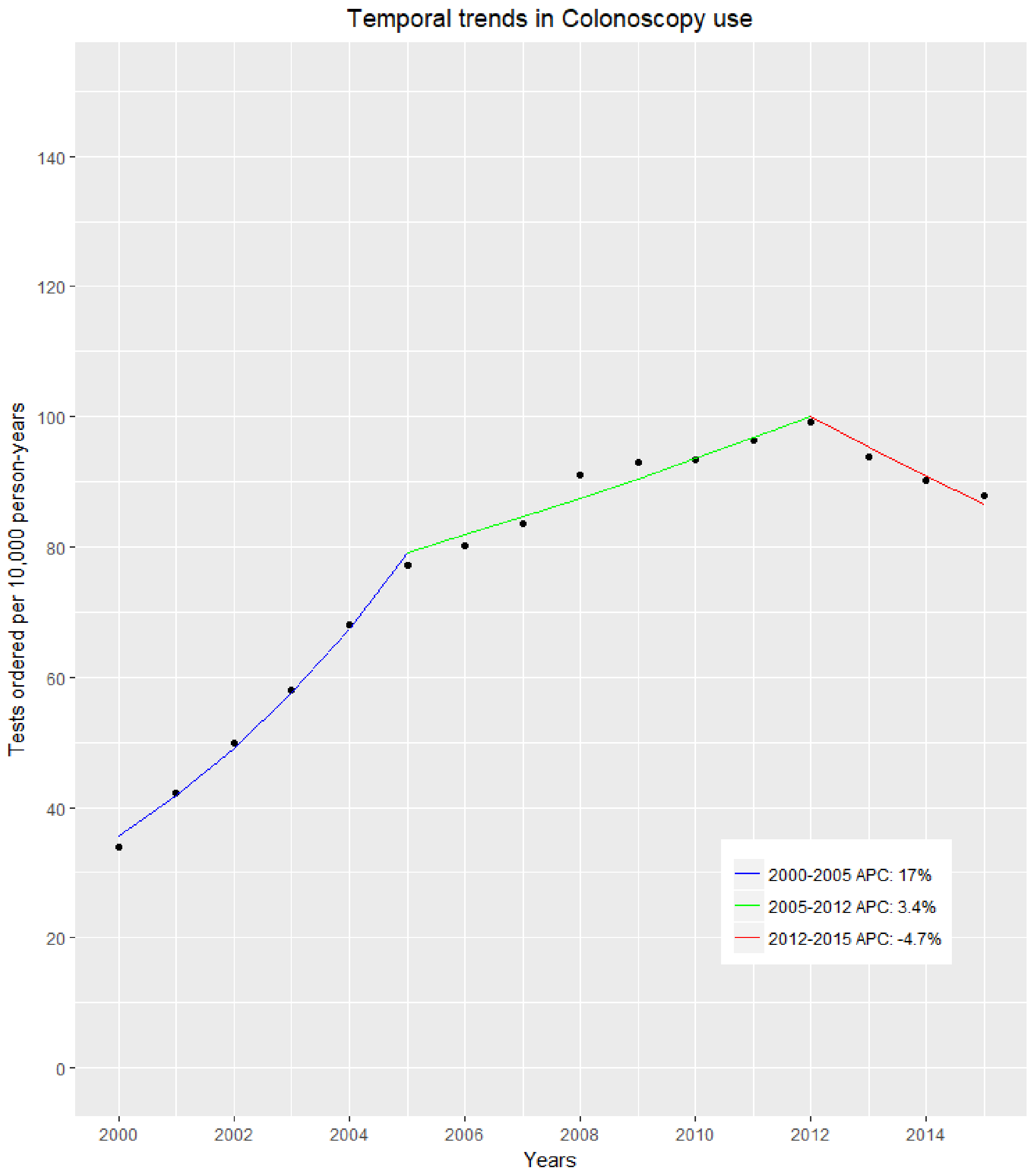
Temporal trends in Colonoscopy use
Tests ordered per 10,000 person-years
140
120
100
80
60
40
20
0
2000
2002
2004
2006
2008
2010
2012
2014
Years
2000-2005 APC: 17%
2005-2012 APC: 3.4%
2012-2015 APC: -4.7%

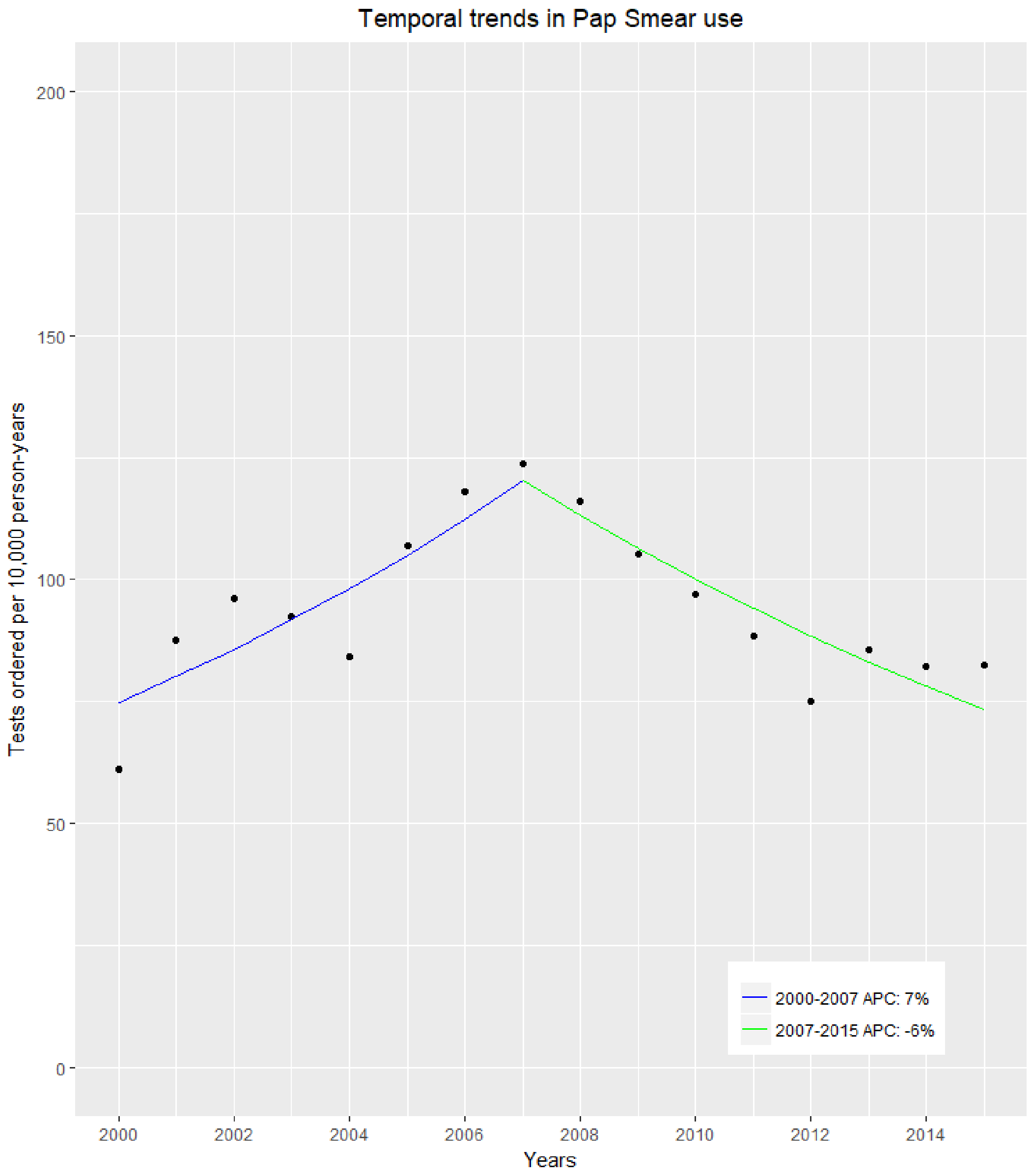
Temporal trends in Pap Smear use
Tests ordered per 10,000 person-years
200
150
100
50
0
2000
2002
2004
2006
2008
2010
2012
2014
Years
2000-2007 APC: 7%
2007-2015 APC: -6%

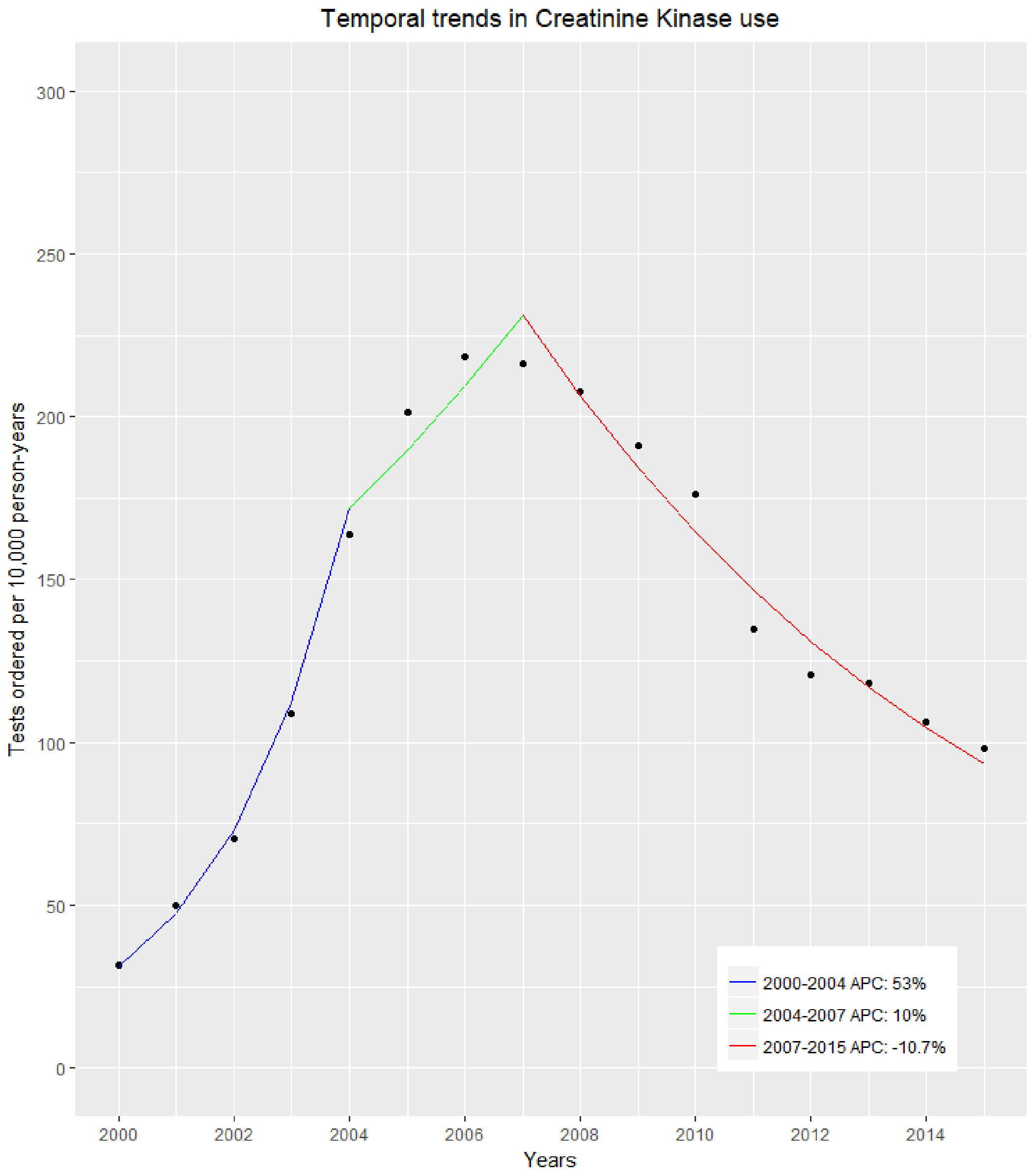
Temporal trends in Creatinine Kinase use
Tests ordered per 10,000 person-years
300
250
200
150
100
50
0
2000
2002
2004
2006
2008
2010
2012
2014
Years
2000-2004 APC: 53%
2004-2007 APC: 10%
2007-2015 APC: -10.7%

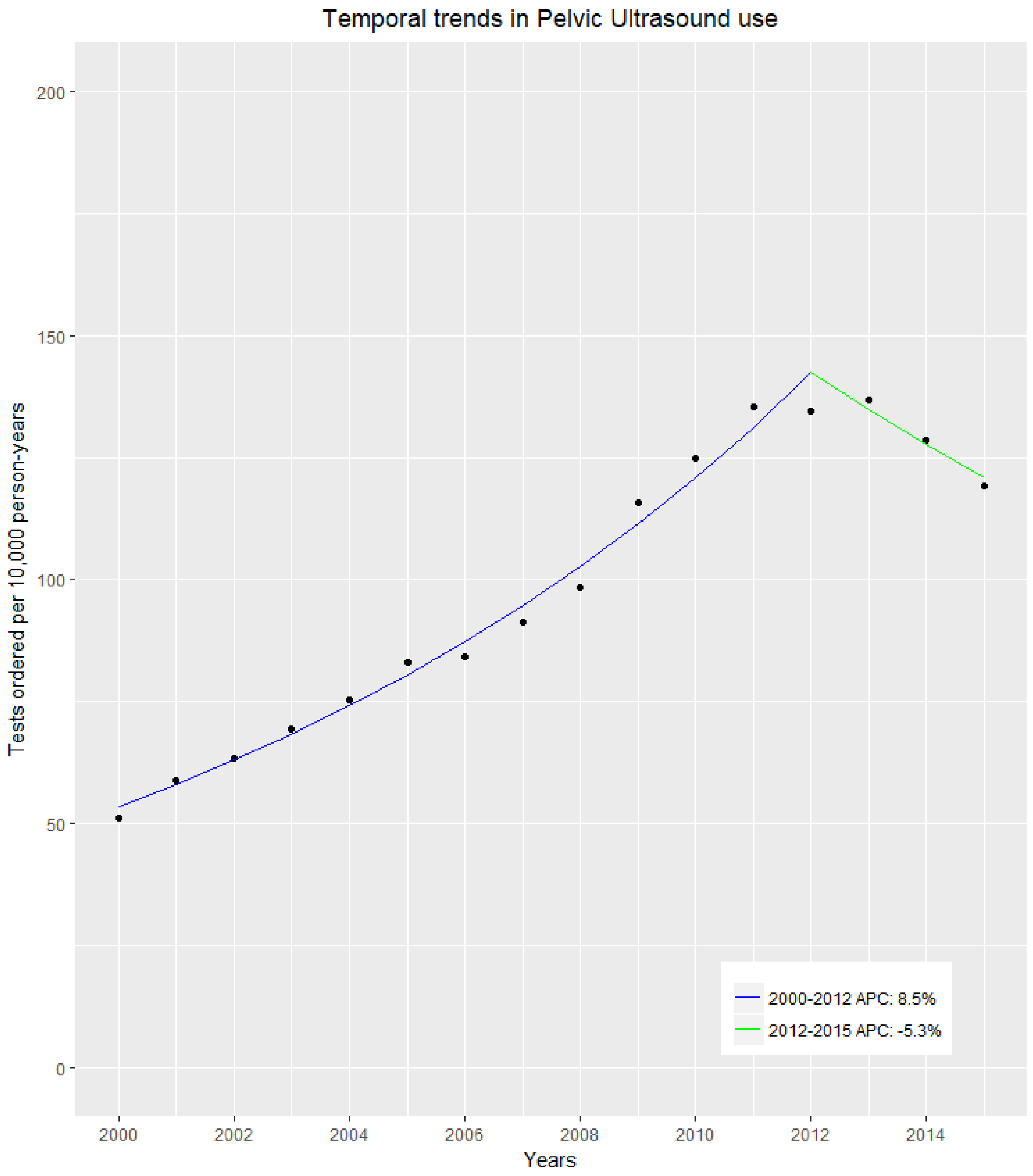

Temporal trends in Pelvic Ultrasound use
Tests ordered per 10,000 person-years
200
150
100
50
0
2000
2002
2004
2006
2008
2010
2012
2014
Years
2000-2012 APC: 8.5%
2012-2015 APC: -5.3%

Pattern: A fall, an increase and then a fall

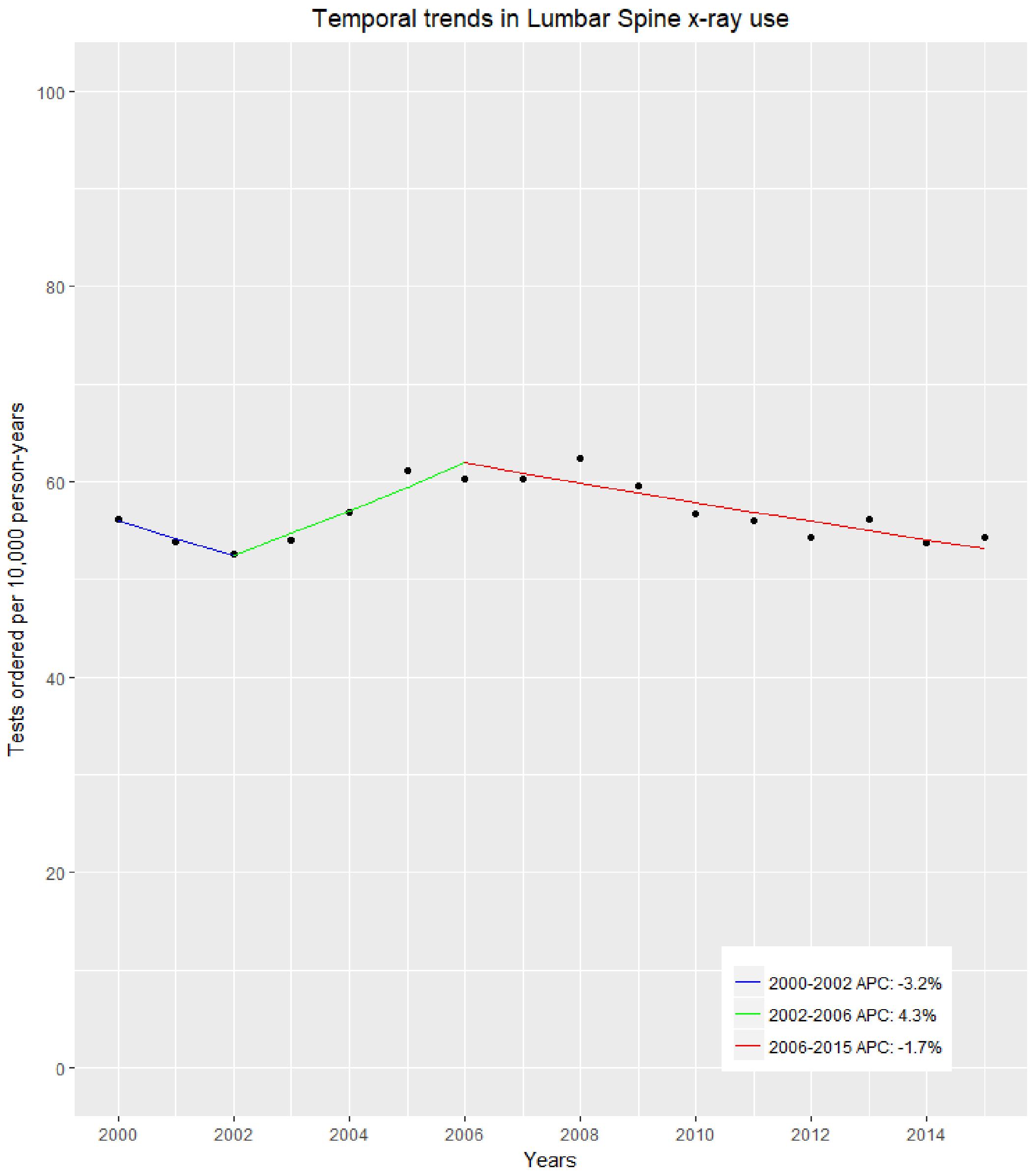
Temporal trends in Lumbar Spine x-ray use
Tests ordered per 10,000 person-years
100
80
60
40
20
0
2000
2002
2004
2006
2008
2010
2012
2014
Years
2000-2002 APC: -3.2%
2002-2006 APC: 4.3%
2006-2015 APC: -1.7%

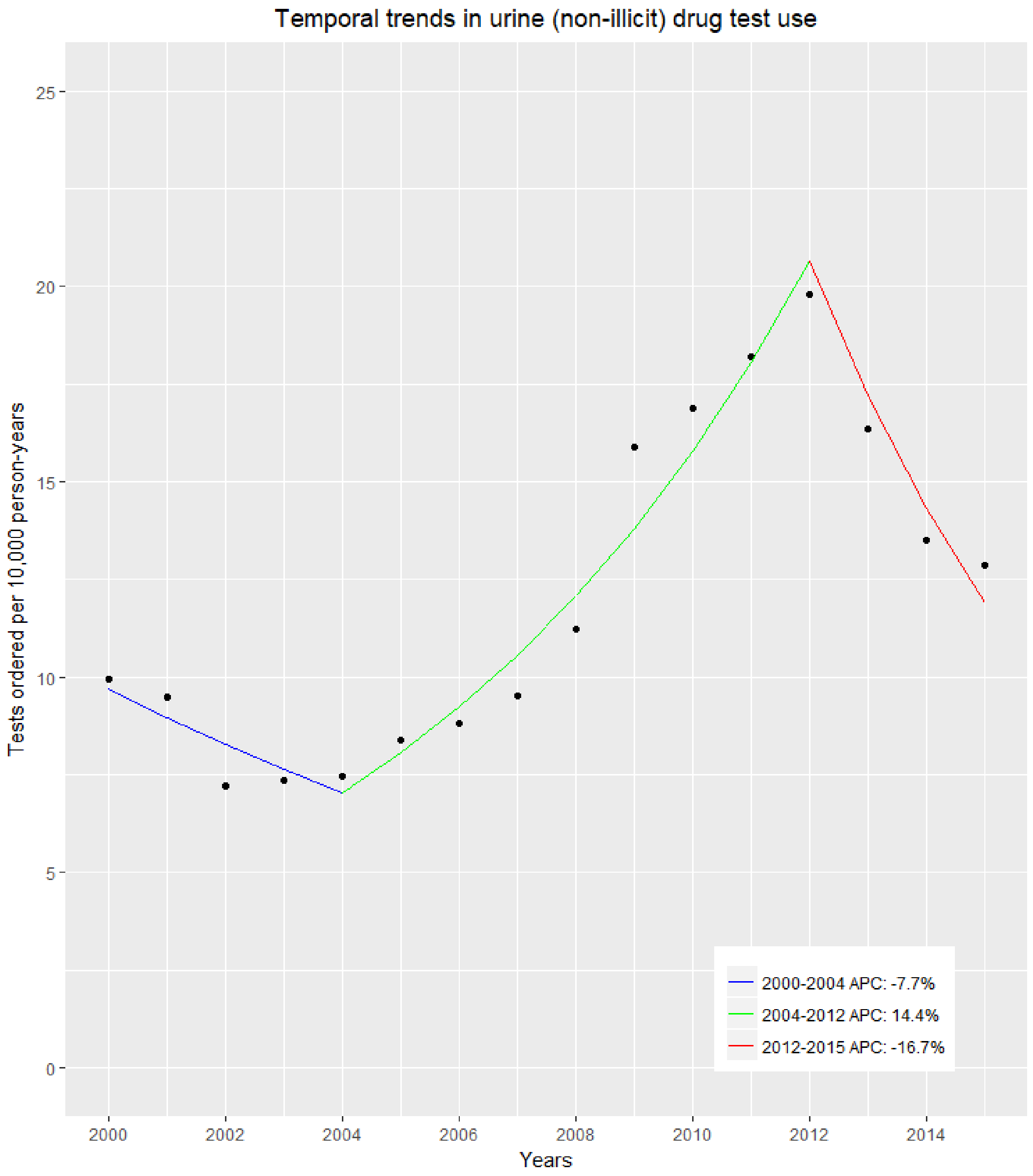

Temporal trends in urine (non-illicit) drug test use
Tests ordered per 10,000 person-years
Years
0
5
10
15
20
25
2000
2002
2004
2006
2008
2010
2012
2014
2000-2004 APC: -7.7%
2004-2012 APC: 14.4%
2012-2015 APC: -16.7%

Pattern: Exceptions

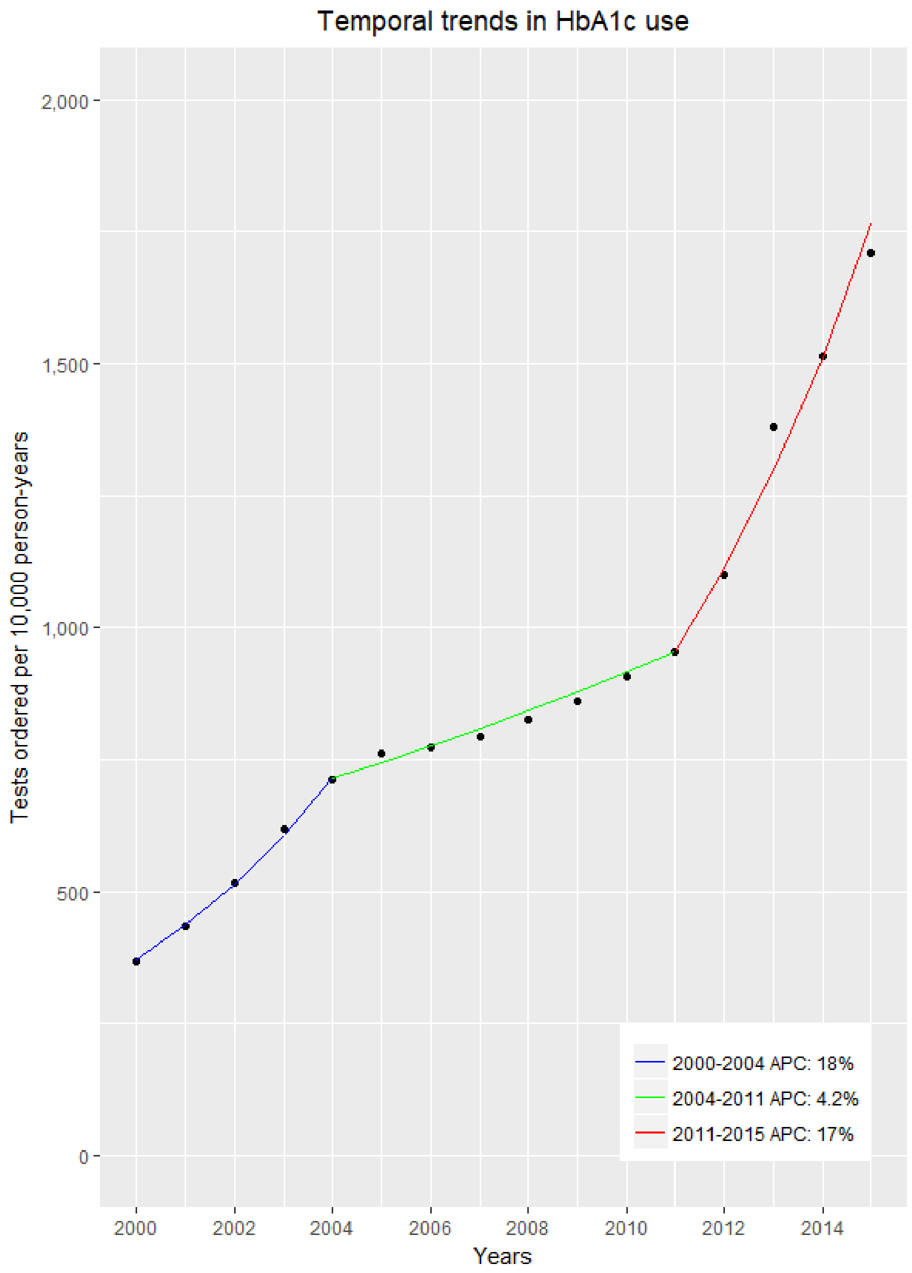

Temporal trends in HbA1c use
Tests ordered per 10,000 person-years
2,000
1,500
1,000
500
0
2000
2002
2004
2006
2008
2010
2012
2014
Years
2000-2004 APC: 18%
2004-2011 APC: 4.2%
2011-2015 APC: 17%

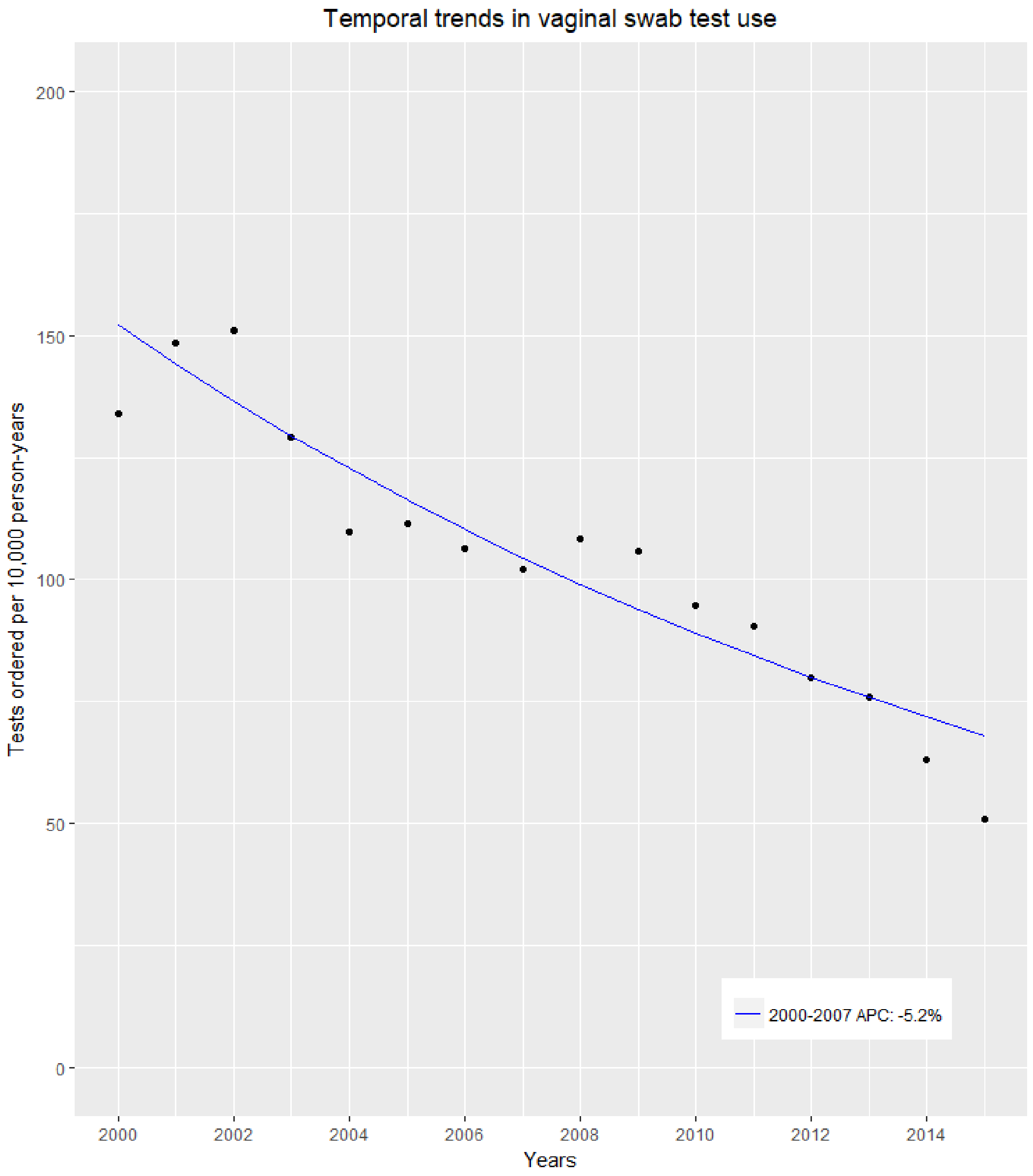

Temporal trends in vaginal swab test use
Tests ordered per 10,000 person-years
200
150
100
50
0
2000
2002
2004
2006
2008
2010
2012
2014
Years
2000-2007 APC: -5.2%
